# Defining myocardial fiber bundle architecture in atrial digital twins

**DOI:** 10.1016/j.compbiomed.2025.109774

**Published:** 2025-02-12

**Authors:** Roberto Piersanti, Ryan Bradley, Syed Yusuf Ali, Alfio Quarteroni, Luca Dede’, Natalia A. Trayanova

**Affiliations:** a MOX - Laboratory of Modeling and Scientific Computing, Dipartimento di Matematica, Politecnico di Milano, Milano, Italy; b ADVANCE - Alliance for Cardiovascular Diagnostic and Treatment Innovation, Johns Hopkins University, Baltimore, USA; c Research Computing, Lehigh University, Bethlehem, PA, USA; d Department of Biomedical Engineering, Johns Hopkins University, Baltimore, USA; e Mathematics Institute, École Polytechnique Fédérale de Lausanne, Lausanne, Switzerland

**Keywords:** Cardiac digital twin, Computational modeling, Cardiac fiber architecture, Numerical simulations, Precision medicine

## Abstract

A key component in developing atrial digital twins (ADT) - virtual representations of patients’ atria — is the accurate prescription of myocardial fibers which are essential for the tissue characterization. Due to the difficulty of reconstructing atrial fibers from medical imaging, a widely used strategy for fiber generation in ADT relies on mathematical models. Existing methodologies utilize semi-automatic approaches, are tailored to specific morphologies, and lack rigorous validation against imaging fiber data. In this study, we introduce a novel atrial Laplace–Dirichlet-Rule-Based Method (LDRBM) for prescribing highly detailed myofiber orientations and providing robust regional annotation in bi-atrial morphologies of any complexity. The robustness of our approach is verified in eight extremely detailed bi-atrial geometries, derived from a sub-millimeter Diffusion-Tensor-Magnetic-Resonance Imaging (DTMRI) human atrial fiber dataset. We validate the LDRBM by quantitatively recreating each of the DTMRI fiber architectures: a comprehensive comparison with DTMRI ground truth data is conducted, investigating differences between electrophysiology (EP) simulations provided by either LDRBM and DTMRI fibers. Finally, we demonstrate that the novel LDRBM outperforms current state-of-the-art (LDRBMs and Universal Atrial Coordinates) fiber models, confirming the exceptional accuracy of our methodology and the critical importance of incorporating detailed fiber orientations in EP simulations. Ultimately, this work represents a fundamental step towards the development of physics-based digital twins of the human atria, establishing a new standard for prescribing fibers in ADT.

## 1. Introduction

Atrial fibrillation (AF), characterized by uncoordinated cardiac activation, is the most common sustained electrical dysfunction of the heart [1,2], and is associated with prominent morbidity and mortality worldwide [3–5]. Despite significant technological and medical advancements, current clinical treatments for AF remain suboptimal [6,7] due to a limited understanding of the complex atrial anatomical substrates which directly sustain AF [8,9]. This is partly because treatments are not personalized to individual patients [10]. Recently, the development of atrial digital twins (ADT) – virtual representations of patients’ atria that integrate computational models with patient specific anatomical and functional data – has provided important insight in the mechanisms underlying AF [11–15]. With recent advancements in high-performance computing, ADT are currently in the early stage of clinical translation [10–18]. ADT are beginning to play a pivotal role in personalized risk assessment, showing promising capabilities in predicting optimal ablation targets for AF [11,15,16,18].

A crucial aspect of developing ADT revolves around accurately representing the arrangement of myocardial fibers, also known as myofibers, which are essential for the tissue characterization [19–22]. Aggregations of myofibers dictates how the electric potential propagates within the muscle [23–25], exhibiting a propagation velocity three-four times faster along the fiber direction than along its orthogonal plane [26]. Moreover, also the muscle mechanical contraction, induced by electrical activation, heavily relies on the fiber architecture [27–30]. Therefore, it is imperative to incorporate the most accurate fiber information possible into ADT. Extremely precise fiber architecture in personalized ADT would enhance the accuracy of these modeling efforts intended for clinical translation.

Over the years, many histo-anatomical studies have explored the fiber arrangement in the atria, revealing a highly intricate texture musculature [31–36]. Atrial fibers are characterized by the presence of multiple overlapping bundles crossing and running along different directions throughout the cardiac chambers [35–37]. Nowadays, comprehensive myofiber information can be obtained through advanced imaging modalities, such as diffusion tensor magnetic resonance imaging (DTMRI) [37,38], micro-computed tomography [39], shear wave imaging [40], or optical mapping [41]. These techniques have proven to be valuable in determining myofiber patterns in ex-vivo hearts [37,38]. However, in-vivo fiber identification [42–44] are still constrained by a relatively coarse spatial resolution [45]. To date, the most comprehensive resource of information regarding the human atrial myofiber structure is an ex-vivo sub-millimeter resolution DTMRI fiber dataset [37]. Nonetheless, this imaging technique required approximately 50 hours for scanning each atrium [37]. For these considerations, contemporary fiber-imaging techniques are practically unusable in the construction of patient-specific ADT, which are models built from geometrical data acquired in-vivo.

In the past decade, multiscale ADT have achieved a high level of biophysical modeling details, incorporating precision in atrial anatomy, tissue characterization, and fibrosis distribution [10,11,46–53]. However, due to the difficulties of acquiring patient-specific fiber data, different methodologies have been proposed to prescribe realistic myocardial fibers for ADT [20,48,51,54]. These are classified in two main groups: atlas-based-methods (ABMs) [47,51,55–59] and rule-based-methods (RBMs) [20,48,60–65]. ABMs are based on establishing a mapping between the patient’s geometry and an oversimplified atlas morphology, previously reconstructed using imaging data or histological information [57,58]. Exploiting this mapping procedure, myofibers are directly transferred from the atlas onto the specific geometry. Nevertheless, ABMs strongly rely on the original data upon which the atlas were constructed, and they are tailored for specific morphologies without considering the highly variability of the atrial anatomy. Conversely, RBMs prescribe fiber orientations through mathematical rules inferred from histological-imaging observations, requiring information solely about the cardiac geometry [20,61]. Classical atrial RBMs rely on manual [48,65] or semi-automatic [61,64] approaches. The former require a considerable amount of hands-on intervention, by introducing several landmarks, seed-points and auxiliaries lines [60,64]. Modern RBMs, known as Laplace–Dirichlet RBMs (LDRBMs) [20,66] have been specifically developed for the atrial chambers [20,67–69]. In LDRBMs, both the fiber fields and atrial regions are determined by solving suitable Laplace boundary-value problems [20]. These methods showed very promising results by representing atrial fibers in diverse morphologies [50,52,68], extending their applicability to encompass geometries at the scale of the whole heart [70–72]. However, they often neglect important fiber bundles and primarily treat the left (LA) and right (RA) atrium as two separate entities, frequently assuming homogeneous fiber transmurality or a simplified monolayer structure [20]. Additionally, LDRBMs lack rigorous validation against the same geometry used for acquiring imaging fiber data.

Motivated by the unresolved issues formally described, here we present a novel atrial LDRBM for prescribing myofiber orientations and providing annotation of anatomical regions in both atria. We begin by introducing the novel atrial LDRBM (Section 2.1), capable of modeling a highly biophysically detailed fiber architecture in volumetric bi-atrial morphologies of any complexity with a highly automated procedure. Building on the foundation of the first atrial LDRBM [20,73], and incorporating key improvements for both LA [68] and RA [71], this method leverages several inter/intra-atrial distances, by solving suitable Laplace–Dirichlet problems, effectively decomposing the bi-atrial anatomy into characteristic anatomical bundle-regions. Solution gradients of these distances establish a local orthonormal coordinate axis system in each bundle. The latter is then utilized to construct and appropriately modulate a volumetric two-layer myofiber field. The method requires the definition of common boundary sets (e.g., endocardium, epicardium, atrioventricular valves and veins rings) and only four landmark points. The assignment of boundary labels is accomplished through an automatic pipeline, ensuring precision, reproducibility and high usability.

To demonstrate the robustness of our approach, we replicate the fiber orientation in eight highly intricate bi-atrial geometries, derived from the original DTMRI human atrial fiber dataset presented in [37]. We establish a systematic measurement procedure (Section 3.2), exploiting the LDRBM reference axis system, to quantify the local myocardial fiber angle across the atrial wall. Then, we validate the novel LDRBM by quantitatively recreating the eight DTMRI fiber architectures. A comprehensive comparison between LDRBM fiber reconstructions and DTMRI ground truth data is conducted, analyzing differences in fiber orientations (Section 3.3). Then, numerical electrophysiology (EP) simulations of the electrical wave propagation, provided by both LDRBM and DTMRI fibers, are evaluated on the same geometries (Section 3.4). Finally, the results obtained by our novel bi-atrial LDRBM, both for the fiber directions and EP simulations, are compared with three state-of-the-art atrial fiber generation models [20,59,67] against ground truth DTMRI fibers (Section 3.5).

## 2. Methods

In this section, we provide a detailed description of the novel bi-atrial LDRBM for the generation of atrial myofibers (Section 2.1).

### 2.1. The bi-atrial Laplace-Dirichlet rules-based method

**Fig. 1.**
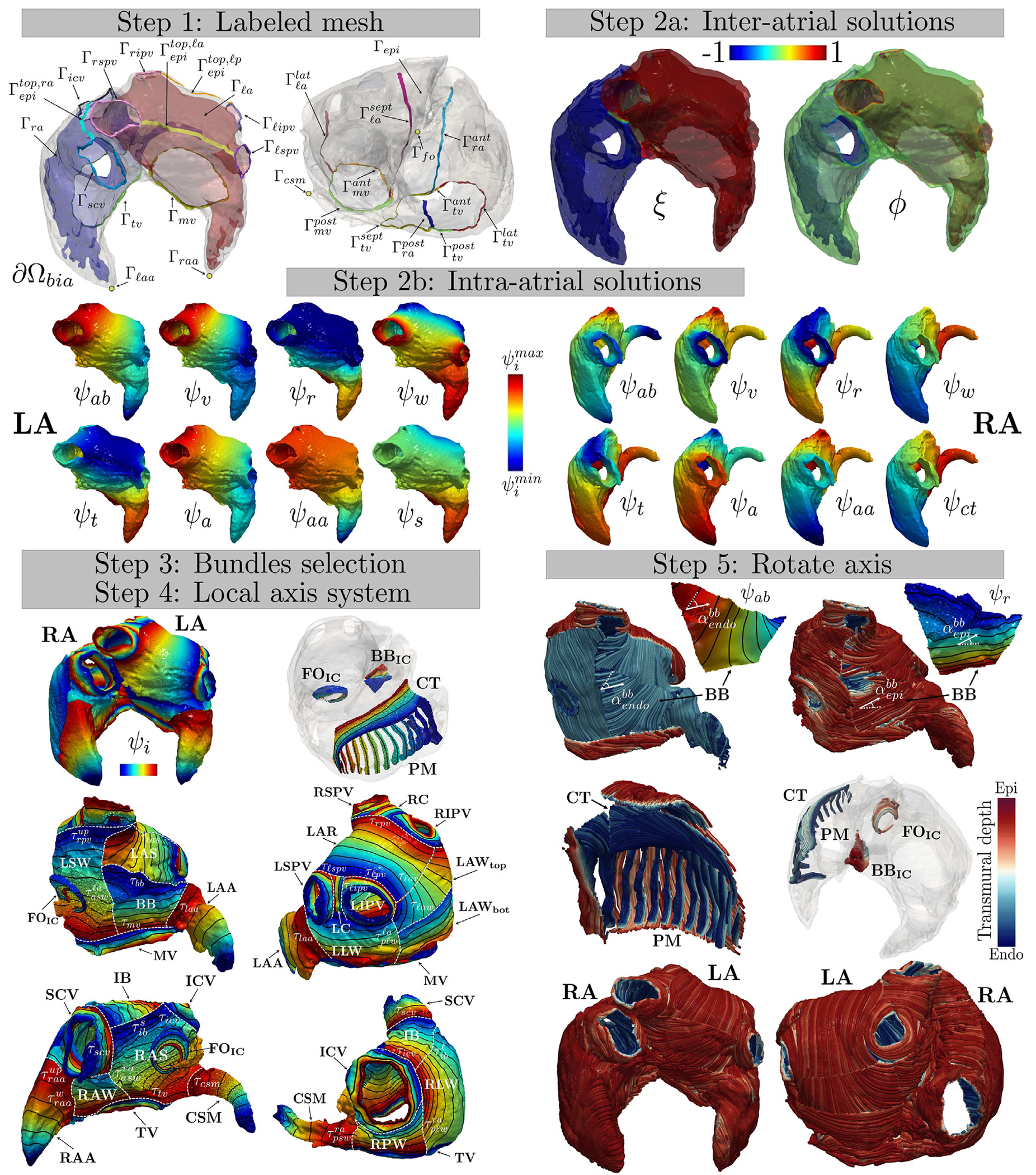
Schematic representation of the atrial LDRBM in a real bi-atrial geometry (derived from the DTMRI human atrial fiber dataset [37]). Step 1: Labeling the mesh to identify partitions of the atrial boundary $\partial \Omega_{\mathrm{bia}}$; Steps 2a–2b: Solving Laplace–Dirichlet problems to define inter-atrial (ξ and ϕ, Steps 2a) and intra-atrial ($\psi_{i}$, with i=ab,v,r,w,t,a,aa,s,ct, Steps 2b) distances; Step 3: Dividing the atrial domain into several anatomical subregions, referred to as bundles; Step 4: Constructing an orthonormal coordinate axis system using the gradients of the inter/intra-atrial distances. The “flat” fiber direction consists of vectors placed along the isochrones lines of the specific intra-atrial distance $\psi_{i}$ chosen for that bundle. Step 5: Rotating the reference frame in each bundle to define the myofiber orientations.

The proposed bi-atrial LDRBM is defined by the following steps which are hereby reported:

1. **Labeled mesh:** provide a labeled mesh of the atrial domain $\Omega_{\mathrm{bia}}$ to identify specific partitions of the atrial boundary $\partial \Omega_{\mathrm{bia}}$, see Step 1 in Fig. 1;
2. **Laplace solutions:** define several inter-atrial and intra-atrial distances by solving specific Laplace–Dirichlet boundary value problems, see Steps 2a–2b in Fig. 1;
3. **Bundles selection:** divide the atrial domain $\Omega_{\mathrm{bia}}$ in several anatomical subregions, named bundles, by establishing their dimensions according to the rules reported in Algorithm 1–3, see Step 3 in Fig. 1. During this step the gradients of the inter/intra-atrial distances are used to build a *transmural* γ and *normal* k directions;
4. **Local coordinate axis system:** construct an orthonormal coordinate axis system for each point of the atrial domain. This system comprises unit vectors representing the “flat” myofiber field, including the *transmural* ${\overset{ˆ}{e}}_{t}$, *normal* ${\overset{ˆ}{e}}_{n}$, and *longitudinal* ${\overset{ˆ}{e}}_{l}$ direction (orthogonal to the former ones), see Step 4 in Fig. 1;
5. **Rotate axis:** rotate the reference frame with the purpose of defining the myofiber orientations, composed by the *fiber* f, the *sheet-normal* n and the *sheet* s directions, see Step 5 in Fig. 1. Rotations are chosen in order to match histology observations and/or DTMRI measurements.

In what follows, we fully detail the five steps of the bi-atrial LDRBM. We refer to Fig. 1 for a schematic representation of the method in a real geometry (retrieved from the DTMRI fiber dataset [37]).

**1. Labeled mesh:** provide a labeled mesh of the bi-atrial computational domain $\Omega_{\mathrm{bia}}$ to define the following boundary partitions $\partial \Omega_{\mathrm{bia}}$ (see Step 1 in Fig. 1)

$$\partial \Omega_{\mathrm{bia}}=\Gamma_{\ell a}\cup \Gamma_{\mathrm{ra}}\cup \Gamma_{\mathrm{epi}}\cup \Gamma_{\mathrm{rpv}}\cup \Gamma_{\ell pv}\cup \Gamma_{\mathrm{scv}}\cup \Gamma_{\mathrm{icv}}\cup \Gamma_{\mathrm{mv}}\cup \Gamma_{\mathrm{tv}}\cup \Gamma_{\ell aa}\cup \Gamma_{\mathrm{raa}}\cup \Gamma_{\mathrm{fo}}\cup \Gamma_{\mathrm{csm}},$$

where $\Gamma_{\ell a},\Gamma_{\mathrm{ra}}$ are the LA and RA endocardium, $\Gamma_{\mathrm{epi}}$ the atrial epicardium, $\Gamma_{\mathrm{rpv}},\Gamma_{\ell pv}$ the right (RPV) and left (LPV) pulmonary vein rings, $\Gamma_{\mathrm{scv}},\Gamma_{\mathrm{icv}}$ the superior (SCV) and inferior (ICV) caval vein rings, $\Gamma_{\mathrm{mv}},\Gamma_{\mathrm{tv}}$ the mitral (MV) and tricuspid (TV) valve rings, $\Gamma_{\mathrm{raa}},\Gamma_{\ell aa}$ the left (LAA) and right (RAA) atrial appendage apices, $\Gamma_{\mathrm{fo}}$ the fossa ovalis (FO) center, and $\Gamma_{\mathrm{csm}}$ the coronary sinus muscle (CSM) apex. In particular, $\Gamma_{\ell a}\left(\Gamma_{\mathrm{ra}}\right)$ is divided into the septal $\Gamma_{\ell a}^{\mathrm{sept}}$ (anterior $\Gamma_{\mathrm{ra}}^{\mathrm{ant}}$) band, the lateral $\Gamma_{\ell a}^{\mathrm{lat}}$ (posterior $\Gamma_{\mathrm{ra}}^{\mathrm{post}}$) band, and the remaining endocardial part $\Gamma_{\ell a}^{\mathrm{endo}}$ $\left(\Gamma_{\mathrm{ra}}^{\mathrm{endo}}\right)$ such that $\Gamma_{\ell a}=\Gamma_{\ell a}^{\mathrm{endo}}\cup \Gamma_{\ell a}^{\mathrm{sept}}\cup \Gamma_{\ell a}^{\mathrm{lat}}\left(\Gamma_{\mathrm{ra}}=\Gamma_{\mathrm{ra}}^{\mathrm{endo}}\cup \Gamma_{\mathrm{ra}}^{\mathrm{ant}}\cup \Gamma_{\mathrm{ra}}^{\text{post}}\right)$. Moreover, $\Gamma_{\mathrm{epi}}$ is splitted into $\Gamma_{\mathrm{epi}}=\Gamma_{\mathrm{epi}}^{-}\cup \Gamma_{\mathrm{epi}}^{top,\ell a}\cup \Gamma_{\mathrm{epi}}^{top,lp}\cup \Gamma_{\mathrm{epi}}^{top,ra}$, where $\Gamma_{\mathrm{epi}}^{top,\ell a}/\Gamma_{\mathrm{epi}}^{top,lp}\left(\Gamma_{\mathrm{epi}}^{top,ra}\right)$ is a boundary label connecting the upper region of anterior/posterior LPV (SCV) to anterior/posterior RPV (ICV) rings, and $\Gamma_{\mathrm{epi}}^{-}$ is the remaining epicardial surface. Furthermore, $\Gamma_{*pv}$ (with *= ℓ,r) encloses the left/right superior pulmonary vein ring (LSPV/RSPV) $\Gamma_{*spv}$ and the left/right inferior pulmonary vein ring (LIPV/RIPV) $\Gamma_{*ipv}$, such that $\Gamma_{*pv}=\Gamma_{*spv}\cup \Gamma_{*ipv}$. Finally, for LA $\Gamma_{\mathrm{mv}}=\Gamma_{\mathrm{mv}}^{\mathrm{ant}}\cup \Gamma_{\mathrm{mv}}^{\mathrm{post}}$, where $\Gamma_{\mathrm{mv}}^{\mathrm{ant}}$ and $\Gamma_{\mathrm{mv}}^{\mathrm{post}}$ are the ring sections facing the anterior and posterior wall, respectively; whereas, for RA $\Gamma_{\mathrm{tv}}=\Gamma_{\mathrm{tv}}^{\mathrm{ant}}\cup \Gamma_{\mathrm{tv}}^{\mathrm{post}}\cup \Gamma_{\mathrm{tv}}^{\mathrm{sept}}\cup \Gamma_{\mathrm{tv}}^{\mathrm{lat}}$, where $\Gamma_{\mathrm{tv}}^{\mathrm{ant}},\Gamma_{\mathrm{tv}}^{\mathrm{post}},\Gamma_{\mathrm{tv}}^{\text{sept}}$, and $\Gamma_{\mathrm{tv}}^{\mathrm{lat}}$ are the ring portions related to the anterior, posterior, septal and lateral wall, respectively, see Step 1 in Fig. 1. A detailed description about the labeling procedure on a generic bi-atrial geometry is given in Appendix A.

**2. Laplace solutions:** define several inter/intra-atrial distances obtained by solving Laplace problems with proper Dirichlet boundary conditions on the atrial boundaries (see Steps 2a–2b in Fig. 1) in the form

(1) $$\left\{\begin{array}{ll} -\Delta \chi =0 & \text{in}\;\Omega_{\mathrm{bia}}\\ \chi =\chi_{a} & \text{on}\;\Gamma_{a}\\ \chi =\chi_{b} & \text{on}\;\Gamma_{b}\\ \nabla \chi \cdot n=0 & \text{on}\;\Gamma_{n} \end{array}\right.,$$

for a generic unknown χ and suitable boundary data $\chi_{a},\chi_{b}\in R$ set on generic partitions of the atrial boundary $\Gamma_{a},\Gamma_{b},\Gamma_{n}$, with $\Gamma_{a}\cup \Gamma_{b}\cup$ $\Gamma_{n}=\partial \Omega_{\mathrm{bia}}$ and $\Gamma_{n}=\partial \Omega_{\mathrm{bia}}/\left(\Gamma_{a}\cup \Gamma_{b}\right)$. The values $\chi_{a},\chi_{b}$ are set in order to evaluate specific inter/intra-atrial distances between boundary partitions $\Gamma_{a},\Gamma_{b}$. Refer to Table 1 for the specific choices in problem (1) made by the bi-atrial LDRBM. Specifically, the inter-atrial distances ξ (computed to differentiate between LA and RA, where positive values of ξ identify LA and negative ones RA) and ϕ (evaluated to identify transmural regions from endocardium to epicardium) are introduced, see Step 2a in Fig. 1. To define a consistent transmural distance, two auxiliaries Laplace solutions $\phi_{\ell a}=1-\phi$ and $\phi_{\mathrm{ra}}=1+\phi$ are introduced for LA and RA, respectively. Furthermore, several intra-atrial distances $\psi_{i}$ (with =ab,v,r,w,t,a,aa,s,ct) are computed, see Step 2b in Fig. 1. Explicitly, $\psi_{ab}$ is a solution of the problem (1) with different boundary data prescribed on LPV, RPV, MV, LAA for LA and IVC, SVC, TV, RAA, and CSM for RA; $\psi_{v}$ represents the distance among the pulmonary veins for LA and between the caval veins for RA; $\psi_{r}$ stands for the distance between MV/TV ring and the union of the pulmonary/caval veins and the top epicardial bands of LA/RA; $\psi_{w}$ is the distance from the superior pulmonary veins and the anterior ring of MV to the inferior pulmonary veins and the posterior ring of MV for LA, and between the septal and the lateral ring of TV, passing from the top epicardial band for RA; $\psi_{t}$ is the distance between MV/TV ring and the top epicardial bands of LA/RA; $\psi_{a}$ represent the distance from the septal/anterior to lateral/posterior wall of LA/RA; $\psi_{aa}$ stands for the distance from FO to LAA for LA and from the posterior wall to RAA for RA. Finally, $\psi_{s}$ and $\psi_{ct}$ are computed solely for LA and RA, respectively: $\psi_{s}$ is solved by prescribing suitable boundary conditions for the anterior and posterior parts of MV ring, the anterior and posterior epicardial bands, and the lateral and septal endocardial bands; $\psi_{ct}$ is the distance from the anterior, posterior and septal part of TV ring to the lateral one.

**3. Bundles selection:** define the atrial bundles and their dimensions throughout the domain $\Omega_{\mathrm{bia}}$, see Step 3 in Fig. 1. With this aim, the bi-atrial LDRBM first selects the inter-atrial connections (IC), following the rules reported in Algorithm 1 (**compute_BIA_**) and then compute LA and RA bundles, exploiting the rules reported in Algorithms 2 (**compute_LA_**) and 3 (**compute_RA_**). Algorithms 1–3 identify the principal anatomical atrial regions: for IC, the bachmann’s bundle $\left({BB}_{IC}\right)$, the fossa ovalis (${FO}_{IC}$), and the coronary sinus $\left({CS}_{IC}\right)$ inter-atrial connections; for LA, mitral valve (MV), left and right inferior and superior pulmonary veins (LIPV, RIPV, LSPV, RSPV), left (LC) and right (RC) carina, left atrial appendage (LAA), left anterior septum (LAS), left lateral wall (LLW), left atrial septum (LSW), left top (${LAW}_{\text{top}}$) and bottom (${LAW}_{\text{bot}}$) atrial posterior wall, left atrial roof (LAR), and bachmann’s bundle (BB); for RA, tricuspid valve (TV), inferior (ICV) and superior (SCV) caval veins, right atrial appendage (RAA), coronary sinus musculature (CSM), right atrial septum (RAS), right lateral wall (RLW), right anterior wall (RAW), right posterior wall (RPW), inter-caval bundle (IB), crista terminalis (CT), and pectinate muscles (PM), see Step 3 in Fig. 1.

To specify the bundle dimensions, the threshold parameters $\tau_{i}$ are introduced: for IC, $\tau_{\mathrm{bb}}^{ic,r},\tau_{\mathrm{bb}}^{ic,\ell },\tau_{\mathrm{bb}}^{ic},\tau_{\mathrm{fo}}^{ic,r},\tau_{\mathrm{fo}}^{ic,\ell },\tau_{\mathrm{fo}}^{ic},\tau_{\mathrm{fo}}^{ic,in},\tau_{cs}^{ic,r},\tau_{cs}^{ic,\ell },\tau_{cs}^{ic}$ referring to ${BB}_{IC},{FO}_{IC}$, and ${CS}_{IC}$ connections; for LA, $\tau_{\mathrm{mv}},\tau_{\ell pv},\tau_{\ell pv}^{up},\tau_{\ell ipv},\tau_{\ell spv},\tau_{\mathrm{rpv}},\tau_{\mathrm{rpv}}^{up},\tau_{\mathrm{ripv}},\tau_{\mathrm{rspv}},\tau_{\ell aa},\tau_{\ell as},\tau_{p\ell w}^{\ell a},\tau_{p\ell w}^{\ell a,up},\tau_{a\ell w}^{\ell a},\tau_{\mathrm{psw}}^{\ell a},\tau_{\mathrm{psw}}^{\ell a,up}$, $\tau_{asw}^{\ell a},\tau_{\ell ar},\tau_{\ell aw},\tau_{\mathrm{bb}}$ referring to MV, LIPV, LSPV, RIPV, RSPV, LAA, LAS, LLW, LSW, LAR, LAW, BB, respectively; for RA, $\tau_{\mathrm{tv}},\tau_{\mathrm{tv}}^{aa},\tau_{\mathrm{icv}},\tau_{\mathrm{icv}}^{up},\tau_{\mathrm{scv}}$, $\tau_{\mathrm{scv}}^{up},\tau_{\mathrm{raa}},\tau_{\mathrm{raa}}^{w},\tau_{\mathrm{raa}}^{up},\tau_{\mathrm{csm}},\tau_{\mathrm{csm}}^{v},\tau_{ras},\tau_{asw}^{\mathrm{ra}},\tau_{asw}^{ra,up},\tau_{a\ell w}^{\mathrm{ra}},\tau_{\mathrm{psw}}^{\mathrm{ra}},\tau_{\mathrm{psw}}^{ra,up},\tau_{p\ell w}^{\mathrm{ra}}$, $\tau_{p\ell w}^{ra,up},\tau_{ib}^{s},\tau_{ib}^{\ell },\tau_{ct}^{+},\tau_{ct}^{-}$, and $\tau_{ct}^{\phi }$ referring to TV, ICV, SCV, RAA, CSM, RAS, RAW, RPW, IB, CT, respectively. Finally, the atrial LDRBM allows to embed PMs in RLW, which requires to specify the parameters ${pm}_{tk}$, ${\mathrm{pm}}_{rg},{pm}_{\text{end}}$, and $N_{pm}$ related to the thickness, range interval, final position and the numbers of PM, respectively, see Step 3 in Fig. 1.

**Algorithm 1:** compute_BIA_: bundles selection for bi-atrial geometry

Let $\tau_{*},\alpha_{\text{endo}}^{*},\alpha_{\mathrm{epi}}^{*}$, be the threshold parameter, the epicardial and the endocardial angles related to IC bundles. Moreover, let ϕ,ξ be the inter-atrial and $\psi_{*}$ the intra-atrial distances.
**if** BB_IC_ = true **and** $\xi \in \left[\tau_{\mathrm{bb}}^{ic,r},\tau_{\mathrm{bb}}^{ic,\ell }\right]$ **and** $\psi_{v}^{\mathrm{ra}}\leq \tau_{\mathrm{bb}}^{ic}$ **then**
$\mathrm{set}\left(\nabla \xi ,\nabla \phi ,\alpha_{\mathrm{epi}}^{bb,ic},\alpha_{\mathrm{epi}}^{bb,ic}\right)⟶{BB}_{IC}$
$\mathrm{flip}\left({\hat{e}}_{l},{\hat{e}}_{t}\right)$
**else if** FO_IC_ = true **and** $\xi \in \left[\tau_{\mathrm{fo}}^{ic,r},\tau_{\mathrm{fo}}^{ic,\ell }\right]$ **and** $\psi_{v}^{\mathrm{ra}}>\tau_{\mathrm{bb}}^{ic}$ **and** $\psi_{aa}^{\ell a}\in \left[\tau_{\mathrm{fo}}^{ic},\tau_{\mathrm{fo}}^{ic,in}\right]$ **then**
$\mathrm{set}\left(\nabla \xi ,\nabla \Psi_{aa}^{\ell a},\alpha_{\mathrm{epi}}^{fo,ic},\alpha_{\mathrm{epi}}^{fo,ic}\right)⟶{FO}_{IC}$
**else if** CS_IC_ = true **and** $\xi \in \left[\tau_{cs}^{ic,r},\tau_{cs}^{ic,\ell }\right]$ **and** $\psi_{v}^{\mathrm{ra}}>\tau_{\mathrm{bb}}^{ic}$ **and** $\psi_{ab}^{\mathrm{ra}}\leq \tau_{cs}^{ic}$ **then**
$\mathrm{set}\left(\nabla \xi ,\nabla \phi ,0,0\right)\to {\text{CS}}_{\text{IC}}$
$\mathrm{flip}\left({\overset{ˆ}{e}}_{l},{\hat{e}}_{t}\right)$
**else if** ξ>0 **then**
**compute_LA_**
**else**
**compute_RA_**

Note: we use $\psi_{i}^{\mathrm{ra}}$ and $\psi_{i}^{\ell a}$ to distinguish LA and RA distances. Moreover, the function $\mathrm{flip}\left({\hat{e}}_{l},{\hat{e}}_{t}\right)$ flips the longitudinal ${\hat{e}}_{l}$ and the transmural ${\overset{ˆ}{e}}_{t}$ directions after the **axis** function (3) evaluation (see Step 4).

During the bundles selection procedure, the LDRBM defines, for each atrial bundle, a unique transmural γ and normal k directions, by taking the gradient of a specific inter-atrial (∇ϕ,∇ξ) or intra-atrial distance $\left(\nabla \psi_{i}\right.$, with $\left.i=ab,v,r,w,t,a,aa,ct,s\right)$. The method also establishes, for each bundle, two rotation angles, denoted as $\alpha_{\text{endo}}$ for the sub-endocardial layer and $\alpha_{\mathrm{epi}}$ for the sub-epicardial one, that are used in Step 5 to properly rotate the myofiber field. This selection process is performed, through Algorithms 1–3, using the following function

(2) $$\left[\gamma ,k,\alpha_{\text{endo}},\alpha_{\text{epi}}\right]=\mathrm{set}\left(\nabla \varphi ,\nabla \vartheta ,\alpha_{\text{endo}}^{j},\alpha_{\mathrm{epi}}^{j}\right)=\left\{\begin{array}{ll} \gamma & =\nabla \varphi \\ k & =\nabla \vartheta \\ \alpha_{\text{endo}} & =\alpha_{\text{endo}}^{j}\\ \alpha_{\mathrm{epi}} & =\alpha_{\mathrm{epi}}^{j} \end{array}\right.,$$

where φ and ϑ represent generic inter/intra-atrial distances, while $\alpha_{\text{endo}}^{j}$ and $\alpha_{\mathrm{epi}}^{j}$ are the prescribed fiber rotation angles for the generic j-th bundle.

The complete bundles selection procedure for the atrial LDRBM is fully detailed in Algorithms 1–3, see also Step 3 in Fig. 1.

**4. Local coordinate axis system:** build for each point of the atrial domain $\Omega_{\mathrm{bia}}$ an orthonormal local coordinate axial system Q

(3) $$Q=\left[{\hat{e}}_{l},{\hat{e}}_{n},{\hat{e}}_{t}\right]=\mathrm{axis}(\gamma ,k)=\left\{\begin{array}{ll} {\overset{ˆ}{e}}_{t} & =\frac{\gamma }{\left\|\gamma \right\|}\\ {\hat{e}}_{n} & =\frac{k-\left(k\cdot {\hat{e}}_{t}\right){\hat{e}}_{t}}{\left\|k-\left(k\cdot {\hat{e}}_{t}\right){\hat{e}}_{t}\right\|}\\ {\hat{e}}_{l} & ={\hat{e}}_{n}\times {\hat{e}}_{t} \end{array}\right.,$$

composed by the unit longitudinal ${\overset{ˆ}{e}}_{l}$, transmural ${\overset{ˆ}{e}}_{t}$ and normal ${\hat{e}}_{n}$ directions, which represent the “flat” myofibers field. It is important to emphasize that each atrial bundle features a distinct local coordinate axial system Q, which nevertheless remains consistent within the bundle itself. By construction, the ${\hat{e}}_{l}$ fields (i.e. the “flat” fiber directions) consists of vectors placed along the isochrones lines of the specific intra-atrial distance $\psi_{i}$ chosen for that bundle, see Step 4 in Fig. 1.

**Algorithm 2:** compute_LA_: bundles selection for LA

Let $\tau_{*},\alpha_{\text{endo}}^{*},\alpha_{\mathrm{epi}}^{*}$ be the threshold parameter, the epicardial and the endocardial angles related to LA bundles. Moreover, let $\phi_{\ell a}=1-\phi$ (with ϕ the transmural distance) and $\psi_{*}$ the LA intra-atrial distances.
**if** $\psi_{r}\geq \tau_{\mathrm{mv}}\;\text{then}\;\mathrm{set}\left(\nabla \phi ,\nabla \psi_{r},\alpha_{\mathrm{endo}}^{\mathrm{mv}},\alpha_{\mathrm{epi}}^{\mathrm{mv}}\right)⟶MV$
**else if** $\psi_{v}\geq \tau_{\mathrm{rpv}}\;\text{and}\;\psi_{r}\leq \tau_{\mathrm{rpv}}^{up}$ **then**
**if** $\psi_{w}\leq \tau_{\mathrm{ripv}}\;\text{then}\;\mathrm{set}\left(\nabla \phi ,\nabla \psi_{v},\alpha_{\mathrm{endo}}^{\mathrm{ripv}},\alpha_{\mathrm{epi}}^{\mathrm{ripv}}\right)⟶RIPV$
**else if** $\psi_{w}\geq \tau_{\mathrm{rspv}}\;\text{then}\;\mathrm{set}\left(\nabla \phi ,\nabla \psi_{v},\alpha_{\text{endo}}^{\mathrm{rspv}},\alpha_{\mathrm{epi}}^{\mathrm{rspv}}\right)⟶RSPV$
**else** $\mathrm{set}\left(\nabla \phi ,\nabla \psi_{w},\alpha_{\mathrm{endo}}^{rc},\alpha_{\mathrm{epi}}^{rc}\right)⟶RC$
**else if** $\psi_{v}\leq \tau_{\ell pv}\;\text{and}\;\psi_{r}\leq \tau_{\ell pv}^{up}$ **then**
**if** $\psi_{w}\leq \tau_{\ell ipv}\;\text{then}\;\mathrm{set}\left(\nabla \phi ,\nabla \psi_{v},\alpha_{\mathrm{endo}}^{\ell ipv},\alpha_{\mathrm{epi}}^{\ell ipv}\right)⟶\text{LIPV}$
**else if** $\psi_{w}\geq \tau_{\ell spv}\;\text{then}\;\mathrm{set}\left(\nabla \phi ,\nabla \psi_{v},\alpha_{\mathrm{endo}}^{\ell spv},\alpha_{\mathrm{epi}}^{\ell spv}\right)⟶\text{LSPV}$
**else** $\mathrm{set}\left(\nabla \phi ,\nabla \psi_{w},\alpha_{\mathrm{endo}}^{\ell c},\alpha_{\mathrm{epi}}^{\ell c}\right)⟶LC$
**else if** $\psi_{aa}\leq \tau_{\ell aa}\;\text{then}\;\mathrm{set}\left(\nabla \phi ,\nabla \psi_{aa},\alpha_{\mathrm{endo}}^{\ell aa},\alpha_{\mathrm{epi}}^{\ell aa}\right)⟶\text{LAA}$
**else if** $\psi_{s}\leq \tau_{\ell as}$ **then**
**if** $\psi_{a}\leq \tau_{p\ell w}^{\ell a}\;\text{and}\;\psi_{t}\geq \tau_{p\ell w}^{\ell a,up}$ **then**
$\mathrm{set}\left(\nabla \phi ,\nabla \psi_{r},\alpha_{\mathrm{endo}}^{\ell w},\alpha_{\mathrm{epi}}^{\ell w}\right)⟶\text{LLW}$
**else if** $\psi_{a}\leq \tau_{p\ell w}^{\ell a}\;\text{and}\;\psi_{t}<\tau_{p\ell w}^{\ell a,up}$ **then**
$\mathrm{set}\left(\nabla \phi ,\nabla \psi_{ab},\alpha_{\mathrm{endo}}^{\ell ar},\alpha_{\mathrm{epi}}^{\ell ar}\right)⟶\text{LAR}$
**else if** $\psi_{a}\geq \tau_{\mathrm{psw}}^{\ell a}\;\text{and}\;\psi_{t}\geq \tau_{\mathrm{psw}}^{\ell a,up}$ **then**
$\mathrm{set}\left(\nabla \phi ,\nabla \psi_{r},\alpha_{\mathrm{endo}}^{sw},\alpha_{\mathrm{epi}}^{sw}\right)⟶\text{LSW}$
**else if** $\psi_{a}\geq \tau_{\mathrm{psw}}^{\ell a}\;\text{and}\;\psi_{t}<\tau_{\mathrm{psw}}^{\ell a,up}$ **then**
$\mathrm{set}\left(\nabla \phi ,\nabla \psi_{ab},\alpha_{\mathrm{endo}}^{\ell ar},\alpha_{\mathrm{epi}}^{\ell ar}\right)⟶LAR$
**else if** $\psi_{w}>0\;\text{then}\;\mathrm{set}\left(\nabla \phi ,\nabla \psi_{ab},\alpha_{\mathrm{endo}}^{\ell ar},\alpha_{\mathrm{epi}}^{\ell ar}\right)⟶\text{LAR}$
**else if** $\psi_{t}\leq \tau_{\ell ar}\;\text{then}\;\mathrm{set}\left(\nabla \phi ,\nabla \psi_{ab},\alpha_{\mathrm{endo}}^{\ell ar},\alpha_{\mathrm{epi}}^{\ell ar}\right)⟶LAR$
**else if** $\psi_{r}\leq \tau_{\ell aw}$ **then**
$\mathrm{set}\left(\nabla \phi ,\nabla \psi_{v}\left(\phi_{\ell a}<0\right)+\nabla \psi_{ab}\left(\phi_{\ell a}\geq 0\right),\alpha_{\text{endo}}^{\ell aw,t},\alpha_{\mathrm{epi}}^{\ell aw,t}\right)⟶{LAW}_{\text{top}}$
**else**
$\mathrm{set}\left(\nabla \phi ,\nabla \psi_{v}\left(\phi_{\ell a}<0\right)+\nabla \psi_{r}\left(\phi_{\ell a}\geq 0\right),\alpha_{\mathrm{endo}}^{\ell aw,b},\alpha_{\mathrm{epi}}^{\ell aw,b}\right)⟶{LAW}_{bot}$
**else if** $\psi_{a}\geq \tau_{asw}^{\ell a}\;\text{then}\;\mathrm{set}\left(\nabla \phi ,\nabla \psi_{r},\alpha_{\text{endo}}^{sw},\alpha_{\mathrm{epi}}^{sw}\right)⟶\text{LSW}$
**else if** $\psi_{a}\leq \tau_{\text{aℓw}}^{\ell a}\;\text{then}\;\mathrm{set}\left(\nabla \phi ,\nabla \psi_{r},\alpha_{\text{endo}}^{\ell w},\alpha_{\mathrm{epi}}^{\ell w}\right)⟶LLW$
**else if** $\psi_{r}\geq \tau_{\mathrm{bb}}$ **then**
$\mathrm{set}\left(\nabla \phi ,\nabla \psi_{ab}\left(\phi_{\ell a}<0\right)+\nabla \psi_{r}\left(\phi_{\ell a}\geq 0\right),\alpha_{\mathrm{endo}}^{\mathrm{bb}},\alpha_{\mathrm{epi}}^{\mathrm{bb}}\right)⟶BB$
**else** $\mathrm{set}\left(\nabla \phi ,\nabla \psi_{ab},\alpha_{\mathrm{endo}}^{\ell as},\alpha_{\mathrm{epi}}^{\ell as}\right)⟶\text{LAS}$

Note: in ${LAW}_{\text{top}},{LAW}_{\text{bot}}$ and BB, we define the normal direction as $k=\nabla \psi_{i}\left(\phi_{\ell a}<0\right)+\nabla \psi_{j}\left(\phi_{\ell a}\geq 0\right)$. This implies that $k=\nabla \psi_{i}$ for $\phi_{\ell a}<0$ (sub-endocardium) and $k=\nabla \psi_{j}$ for $\phi_{\ell a}\geq 0$ (sub-epicardium).

**5. Rotate axis:** rotate the reference frame Q, defined at the previous step for each point of the atrial domain $\Omega_{\mathrm{bia}}$, with the purpose of defining the myofiber orientations, see Step 5 in Fig. 1. This is performed by rotating the longitudinal direction ${\hat{e}}_{l}$ around ${\hat{e}}_{t}$ by means of a suitable angle α

(4) $$[f,n,s]=\mathrm{orient}(Q,\alpha )=\left[{\hat{e}}_{l},{\hat{e}}_{n},{\hat{e}}_{t}\right]R_{{\hat{e}}_{t}}(\alpha ),$$

where $R_{{\hat{e}}_{t}}(\alpha )$ and α are given by

(5) $$R_{{\hat{e}}_{t}}(\alpha )=\left[\begin{array}{ccc} \cos(\alpha ) & -\sin(\alpha ) & 0\\ \sin(\alpha ) & \cos(\alpha ) & 0\\ 0 & 0 & 1 \end{array}\right],\;\alpha =\left\{\begin{array}{cc} \alpha_{\text{endo}} & \text{for}|\phi |>0\\ \alpha_{\text{epi}} & \text{for}|\phi |\leq 0 \end{array}\right.,$$

with $\alpha_{\text{endo}},\alpha_{\text{epi}}$ the rotation angles, on the sub-endocardial (|ϕ|>0) and sub-epicardial (|ϕ|≤0) layers, prescribed in the **set** function (2). The resulting three unit vectors correspond to the final fiber f, sheet s and sheet-normal n directions. From a computational perspective, for each nodal point in the atrial domain, we employ the transmural Laplace solution ϕ, representing the distance from the endocardium to the epicardium, to distinguish between sub-endocardial (ϕ>0) and sub-epicardial (ϕ≤0) layers. In this way a volumetric transmural bilayer (with a sub-endocardial and sub-epicardial) structure is prescribed in each atrial bundle, see Step 5 in Fig. 1. Rotation angles are chosen in order to match histology observations and/or DTMRI measurements.

**Algorithm 3:** compute_RA_: bundles selection for RA

Let $\tau_{*}$, $\alpha_{\mathrm{endo}}^{*},$ $\alpha_{\mathrm{epi}}^{*}$ be the threshold parameter, the epicardial and the endocardial angles related to RA bundles. Moreover, let $N_{\mathrm{pm}}$, ${\mathrm{pm}}_{*}$ the parameters referring to PM bundle. Finally, let $\phi_{\mathrm{ra}}=1+\phi$ (with ϕ the transmural distance) and $\psi_{*}$ the RA intra-atrial distances.
**if** $\psi_{r}\geq \tau_{\mathrm{tv}}$ **and** $\psi_{ab}\geq \tau_{\mathrm{tv}}^{aa}$ **then** $\mathrm{set}\left(\nabla \phi ,\nabla \psi_{r},\alpha_{\mathrm{endo}}^{\mathrm{tv}},\alpha_{\mathrm{epi}}^{\mathrm{tv}}\right)\to \text{TV}$
**else if** $\psi_{v}\leq \tau_{\mathrm{scv}}$ **and** $\psi_{r}\leq \tau_{\mathrm{scv}}^{up}$ **then** $\mathrm{set}\left(\nabla \phi ,\nabla \psi_{v},\alpha_{\mathrm{endo}}^{\mathrm{scv}},\alpha_{\mathrm{epi}}^{\mathrm{scv}}\right)\to \text{SCV}$
**else if** $\psi_{v}\geq \tau_{\mathrm{icv}}$ **and** $\psi_{r}\leq \tau_{\mathrm{icv}}^{up}$ **then** $\mathrm{set}\left(\nabla \phi ,\nabla \psi_{v},\alpha_{\mathrm{endo}}^{\mathrm{icv}},\alpha_{\mathrm{epi}}^{\mathrm{icv}}\right)\to \text{ICV}$
**else if** $\psi_{aa}\leq \tau_{\mathrm{raa}}$ **and** $\psi_{w}\leq \tau_{\mathrm{raa}}^{w}$ **and** $\psi_{t}\geq \tau_{\mathrm{raa}}^{up}$ **then**
$\mathrm{set}\left(\nabla \phi ,\nabla \psi_{aa},\alpha_{\mathrm{endo}}^{\mathrm{raa}},\alpha_{\mathrm{epi}}^{\mathrm{raa}}\right)\to \text{RAA}$
**else if** CSM = true **and** $\psi_{ab}\leq \tau_{\mathrm{csm}}$ **and** $\psi_{v}\geq \tau_{\mathrm{csm}}^{v}$ **then**
$\mathrm{set}\left(\nabla \phi ,\nabla \psi_{ab},\alpha_{\mathrm{endo}}^{\mathrm{csm}},\alpha_{\mathrm{epi}}^{\mathrm{csm}}\right)\to \text{CSM}$
**else if** $\psi_{w}\geq \tau_{ras}$ **then**
**if** $\psi_{a}\leq \tau_{\mathrm{psw}}^{\mathrm{ra}}$ **and** $\psi_{t}\geq \tau_{\mathrm{psw}}^{ra,up}$ **then**
$\mathrm{set}\left(\nabla \phi ,\nabla \psi_{r},\alpha_{\mathrm{endo}}^{pw},\alpha_{\mathrm{epi}}^{p\omega }\right)\to \text{RPW}$
**else if** $\psi_{a}\geq \tau_{asw}^{\mathrm{ra}}$ **and** $\psi_{t}\geq \tau_{asw}^{ra,up}$ **then**
$\mathrm{set}\left(\nabla \phi ,\nabla \psi_{r},\alpha_{\mathrm{endo}}^{raw},\alpha_{\mathrm{epi}}^{raw}\right)\to \text{RAW}$
**else if** $\psi_{t}\leq \tau_{ib}^{S}$ **then** $\mathrm{set}\left(\nabla \phi ,\nabla \psi_{v},\alpha_{\mathrm{endo}}^{ib},\alpha_{\mathrm{epi}}^{ib}\right)\to \text{IB}$
**else** $\mathrm{set}\left(\nabla \phi ,\nabla \psi_{t},\alpha_{\mathrm{endo}}^{ib},\alpha_{\mathrm{epi}}^{ib}\right)\to \text{RAS}$
**else if** $\psi_{a}\leq \tau_{p\ell w}^{\mathrm{ra}}$ **and** $\psi_{t}\geq \tau_{p\ell w}^{ra,up}$ **then**
$\mathrm{set}\left(\nabla \phi ,\nabla \psi_{r},\alpha_{\mathrm{endo}}^{rpw},\alpha_{\mathrm{epi}}^{rpw}\right)\to \text{RPW}$
**else if** $\psi_{a}\geq \tau_{a\ell w}^{\mathrm{ra}}$ **then** $\mathrm{set}\left(\nabla \phi ,\nabla \psi_{r},\alpha_{\mathrm{endo}}^{raw},\alpha_{\mathrm{epi}}^{raw}\right)\to \text{RAW}$
**else if** $\psi_{t}\leq \tau_{ib}^{\ell }$ **then** $\mathrm{set}\left(\nabla \phi ,\nabla \psi_{v},\alpha_{\mathrm{endo}}^{ib},\alpha_{\mathrm{epi}}^{ib}\right)\to \text{IB}$
**else if** $\psi_{ct}\in \left[\tau_{ct}^{-},\tau_{ct}^{+}\right]$ **and** $\phi_{\mathrm{ra}}\leq \tau_{ct}^{\phi }$ **then**
$\mathrm{set}\left(\nabla \phi ,\nabla \psi_{ct},\alpha_{\text{endo}}^{ct},\alpha_{\text{endo}}^{ct}\right)\to \text{CT}$
**else if** $\psi_{ct}>\tau_{ct}^{+}$ **then** $\mathrm{set}\left(\nabla \phi ,\nabla \psi_{ab},\alpha_{\mathrm{endo}}^{r\ell w},\alpha_{\mathrm{epi}}^{r\ell w}\right)\to \text{RLW}$
**else**
**if** PM = true **then**
**if** $\phi_{\mathrm{ra}}>\tau_{ct}^{\phi }$ **then** $\mathrm{set}\left(\nabla \phi ,\nabla \psi_{ab},\alpha_{\mathrm{endo}}^{r\ell w},\alpha_{\mathrm{epi}}^{r\ell w}\right)\to \text{RLW}$
**for** $\text{n}=1:N_{pm}$ **do**
{
${\text{PM}}_{i}=\tau_{\mathrm{raa}}+(n-1)\left({\text{pm}}_{tk}+{\text{pm}}_{rg}\right)$
${\text{PM}}_{f}={\text{PM}}_{i}+{\text{pm}}_{tk}$
${\text{PM}}_{s}={\text{PM}}_{f}+{\text{pm}}_{rg}$
**if** $\psi_{\mathrm{raa}}\in \left({\text{PM}}_{f},{\text{PM}}_{s}\right)$ **or** $\psi_{\mathrm{raa}}>pm_{end}$ **or** $\psi_{\mathrm{raa}}<\tau_{\mathrm{raa}}$ **then**
$\mathrm{set}\left(\nabla \phi ,\nabla \psi_{ab},\alpha_{\mathrm{endo}}^{r\ell w},\alpha_{\mathrm{epi}}^{r\ell w}\right)\to \mathrm{RLW}$
**else if** $\psi_{\mathrm{raa}}\leq {\text{pm}}_{end}$ **and** $\psi_{\mathrm{raa}}\geq {\text{PM}}_{i}$ **and** $\psi_{\mathrm{raa}}\leq {\text{PM}}_{f}$ **then**
$\mathrm{set}\left(\nabla \phi ,\nabla \psi_{\text{raa}},\alpha_{\text{endo}}^{pm},\alpha_{\text{endo}}^{pm}\right)\to \text{PM}$
}
**else** $\mathrm{set}\left(\nabla \phi ,\nabla \psi_{ab},\alpha_{\mathrm{endo}}^{r\ell w},\alpha_{\mathrm{epi}}^{r\ell w}\right)\to \text{RLW}$

## 3. Results

This section is dedicated to present numerical results both for the fiber generation and EP simulations, employing the LDRBM discussed in Section 2.1. We organize this section as follows. Section 3.1 describes the common settings for all the simulations. Section 3.2 presents the measurement procedure, exploiting the LDRBM axis system, to assess the local myocardial fiber angle in bi-atrial geometries embedded with DTMRI data [37]. Section 3.3 illustrates the realization of digital twin atrial fiber architectures using the LDRBM to quantitatively recreate the DTMRI myofiber bundle structures. A comprehensive comparison between LDRBM fiber reconstructions and DTMRI ground truth data is conducted. Section 3.4 showcases EP simulations induced by both LDRBM and DTMRI fibers. Section 3.5 provides a comparison of the proposed LDRBM (presented in Section 2.1) with state-of-the-art atrial fiber models (i.e., the Universal Atrial Coordinates ABM [59] and two other LDRBMs [20,67]) against DTMRI data: we compare the fiber orientations and we analyze their discrepancies in terms of EP activation times.

### 3.1. Simulation settings

All the simulations are performed on real bi-atrial geometries processed from the original ex-vivo DTMRI fiber-geometry dataset established in [37]. This includes eight segmented geometrical models of the human atria, embedding volumetric fiber orientations at a submillimeter resolution, thus providing an unprecedented level of information on both human atrial structure and fibers (see [37] for furhter details). Being an extremely detailed models of the human atria, it demonstrates the applicability robustness of the proposed bi-atrial LDRBM to arbitrary patient-specific scenarios.

To build the computational mesh associated with the bi-atrial geometries, we use the Vascular Modeling Toolkit software vmtk (http://www.vmtk.org) by exploiting the semi-automatic cardiac meshing tools [74] in combination with the software meshmixer (http://www.meshmixer.com). The mesh generation process begins with a pre-processing step in meshmixer, focusing on minimal cleaning and smoothing the atrial surfaces: LA, RA endocardium, and epicardium. This step aims to meticulously separate them while preserving their morphological structures to the fullest extent possible. Then, the surface labeling and tetrahedral volumetric Finite Element (FE) mesh generation is performed in vmtk. The labeling procedure carried out in this work, for the atrial LDRBM (see Step 1 in Section 2.1), is fully detailed in Appendix A. Finally, volumetric DTMRI fibers, embedded in the orginal bi-atrial dataset, were assigned to each nodal point of the labeled computational domains by means of linear projection using vmtk.

For representing EP activity in the atrial tissue, we employ the Eikonal-diffusion model [75,76] (detailed in Appendix B). The numerical approximation of the eikonal model, requires the following physical data: the velocity parameter $c_{f}$ and the conductivities along the myofiber directions $\sigma_{f},\sigma_{s}$ and $\sigma_{n}$. We set $c_{f}=100s^{-1/2},\sigma_{f}=$ $1\times 10^{-4}m^{2}s^{-1}$, and $\sigma_{s}=\sigma_{n}=0.16\times 10^{-4}m^{2}s^{-1}$, in order to achieve the conduction velocities of $1ms^{-1}$ in the fiber direction f and $0.4ms^{-1}$ along the sheet s and normal n directions [20,48,64,77,78]. Finally, to initiate the EP signal propagation, a spherical stimulus, with radius $2\times 10^{-3}m$, is applied at time t=0s in the Sino-Atrial-Node (SAN), which lies in the musculature of CT at the anterolateral junction with SCV [79]. Regarding the mesh element size h and the time step Δt, related to the space and time discretizations of the pseudo-time eikonal equation (see Appendix B), we used continuous FE of order 1 on tetrahedral meshes with an average element size of $h=6\times 10^{-4}m$ and the Backward Difference Formulae (BDF) approximation of order 2 with a time step of $\Delta t=10^{-3}s$. We used this setting values for all the simulations reported in Sections 3.4 and 3.5.

The novel atrial LDRBM (see Section 2.1), the measuring procedure (see Section 3.2) and the Eikonal-diffusion model (see Appendix B) have been implemented and solved using life^x^ [80–83] (https://lifex.gitlab.io), an in-house high-performance C++ FE library focused on cardiac applications based on deal.II FE core [84] (https://www.dealii.org).

For the analyzed DTMRI geometries, the entire bi-atrial LDRBM pipeline (see Section 2.1) requires approximately 130 s on average, using a computational domain of about 600 K degrees of freedom and performed on a standard laptop equipped with a 6 core Intel-R Core-TM i7-9750H processor (2.60 GHz) and 32 GB of RAM. The statistical data analysis performed to estimate the variability of both the regional bundle dimension parameters (see Section 3.2.1) and the atrial fiber angles (see Section 3.2.2) were performed in Matlab (https://www.mathworks.com) and ParaView (https://www.paraview.org). We employed the CircStat toolbox [85] to carry out the circular fiber angle statistics, and the CircHist toolbox (https://github.com/zifredder/CircHist) to perform the polar angle histograms (see Appendix C.1 for further details). Finally, to visualize the results we used ParaView.

All the numerical EP simulations were executed on the cluster iHeart (Lenovo SR950 8 × 24-Core Intel Xeon Platinum 8160, 2100 MHz and 1.7 TB RAM) at MOX, Dipartimento di Matematica, Politecnico di Milano.

### 3.2. Measurement procedure for atrial fiber orientations

**Fig. 2.**
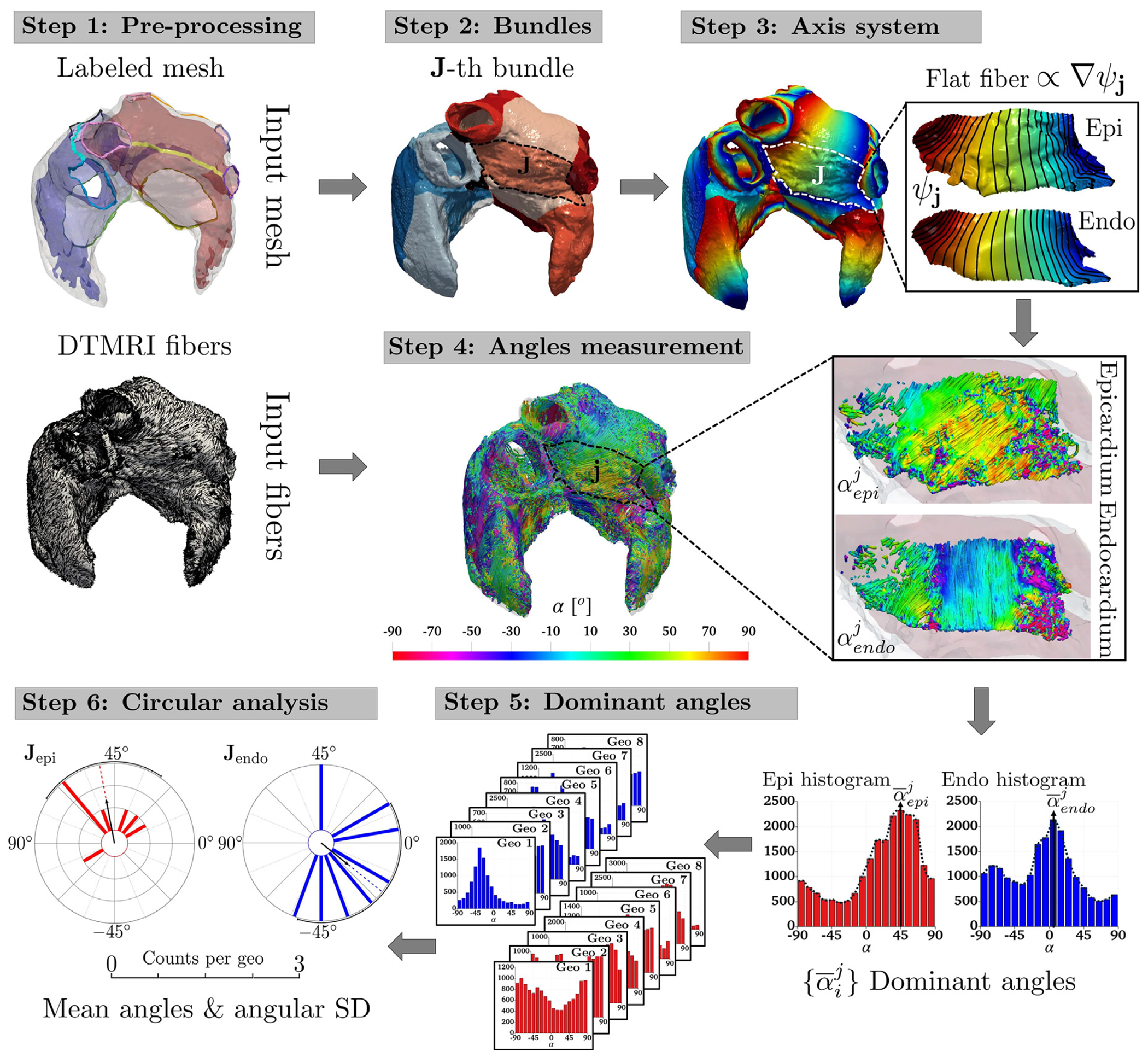
Schematic representation of the measurement procedure exploiting the LDRBM reference axis system to asses the local myocardial fiber angle in each atrial bundles across eight human bi-atrial geometries embedded with DTMRI fiber data [37].

We present hereafter the systematic measurement procedure used to quantify the local myocardial fiber angle in each atrial bundle applied to the eight geometries of the DTMRI fiber dataset [37]. This exploits both the LDRBM bundle subdivisions and the related local coordinate axis system (see Section 2.1) and it is characterized by the following steps (refer to Fig. 2 for a schematic representation of the measurement procedure):

1. **Pre-processing:** Provide a labeled bi-atrial computational mesh and a related DTMRI fiber field. The labeling procedure is performed according to the LDRBM (see Step 1 in Section 2.1), while the DTMRI fibers are linearly projected onto the labeled mesh;
2. **Bundles:** Subdivide the atrial domain into characteristic anatomical regions, named bundles, employing the LDRBM bundle subdivisions (see Step 3 in Section 2.1);
3. **Axis system:** Build a local coordinate axis system $Q=\left[{\overset{ˆ}{e}}_{l},{\hat{e}}_{n},{\hat{e}}_{t}\right]$ composed of the LDRBM flat myofiber vectors (see Step 4 in Section 2.1);
4. **Angles measurement:** Embed the DTMRI fiber data $f_{\text{DTMRI}}^{m}$ within the local coordinate axis system Q, using the following Gram–Schmidt process:
$$\{\begin{array}{l} s_{\text{DTMRI}}=e_{t}\\ f_{\text{DTMRI}}=\frac{f_{\text{DTMRI}}^{m}-\left(f_{\text{DTMRI}}^{m}\cdot e_{t}\right)e_{t}}{∥f_{\text{DTMRI}}^{m}-\left(f_{\text{DTMRI}}^{m}\cdot e_{t}\right)e_{t}∥}\\ n_{\text{DTMRI}}=e_{t}\times f_{\text{MRI}} \end{array},$$
  such that, starting from the experimentally derived DTMRI fiber field $f_{\text{DTMRI}}^{m}$, three associated myofiber directions $f_{\text{DTMRI}},n_{\text{DTMRI}}$, and $s_{\text{DTMRI}}$ are retrieved and embedded in the same space spanned by the LDRBM axis system. Finally, for every node of the computational domain, measure the DTMRI fiber orientation angles $\alpha_{i}^{j}\in (-\pi /2,\pi /2]$, in each bundle, relative to the LDRBM axis system (where j refers to the generic j-th bundle and i indicates the sub-epicardial i=epi and sub-endocardial i=endo layers). This is performed using the function $\alpha_{i}^{j}=arcos\left(f_{DTMRI}\cdot e_{\ell }\right)$ corrected to account for the directional invariance of the fibers. We employ the transmural Laplace solution ϕ (see Step 2 in Section 2.1), to distinguish between sub-endocardial (ϕ>0) and sub-epicardial (ϕ≤0) layers. In this way a volumetric transmural bilayer, is identified in each atrial bundle, see also Step 5 in Section 2.1;
5. **Dominant angles:** Compute histograms, for each bundle, of the measured DTMRI angles $\alpha_{i}^{j}$ and identify the dominant angle ${\overset{‾}{\alpha }}_{i}^{j}$ by selecting the modal values of the distributions;
6. **Circular analysis:** Perform a circular statistical analysis [85–87] (detailed in Appendix C.1) to quantitatively assess the fiber angle variability across the entire dataset.

For the human atrial DTMRI fiber dataset [37], Step 2 is outlined in Section 3.2.1, while Steps 4–6 are described in Section 3.2.2.

#### 3.2.1. Classification of atrial bundles

**Fig. 3.**
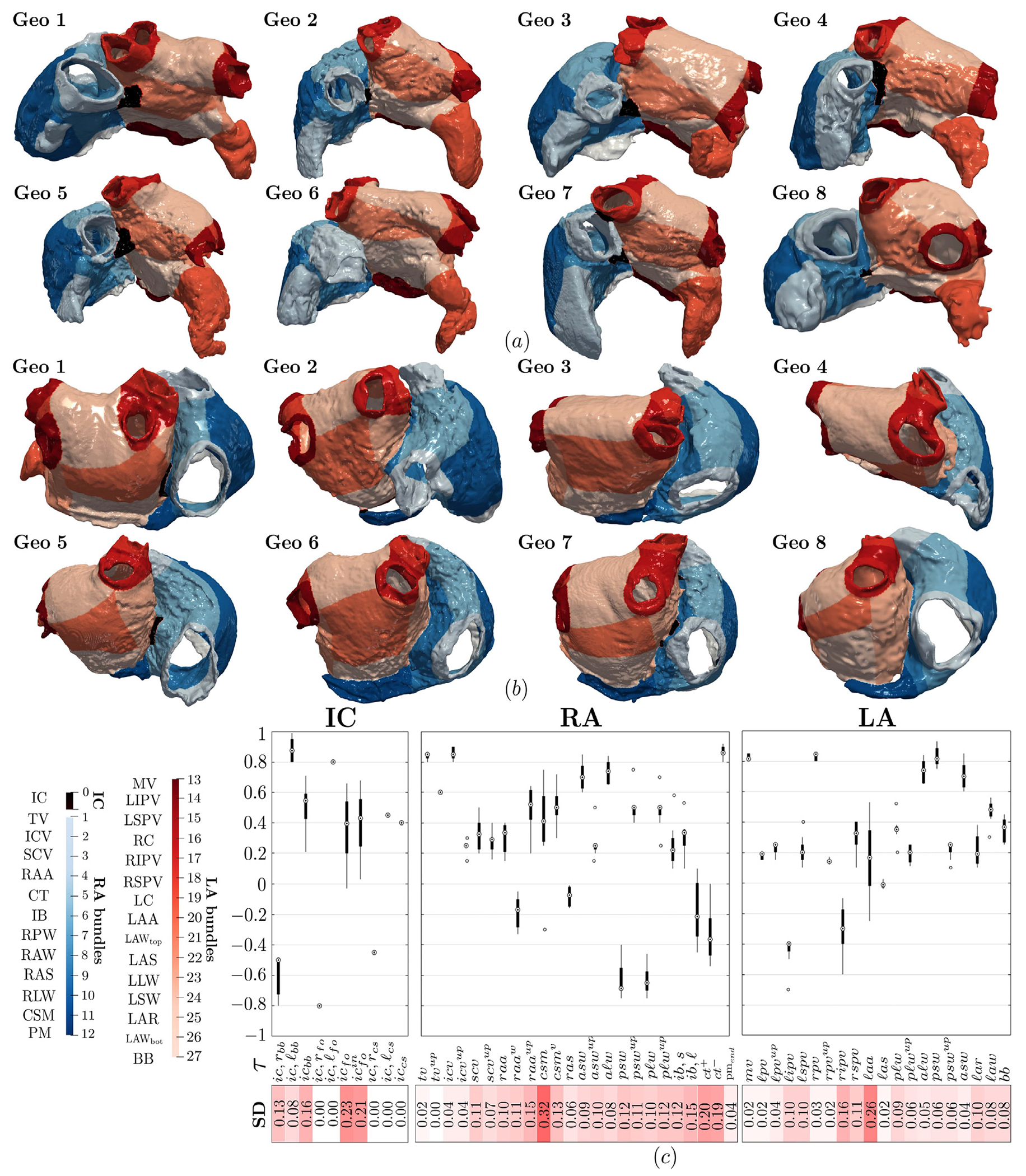
Bundles subdivision performed by the bi-atrial LDRBM for the DTMRI dataset geometries: (a) anterior view; (b) posterior view; (c) box-plots showing the bundle parameter variation for the left atrium (LA), right atrium (RA) and inter-atrial connections (IC); standard deviation (SD) values of the bundle parameters, with SD color-coded on a scale from minimal (0) to maximal (0.32) values (see also Fig. C.1 and Tables C.4–C.6).

**Fig. C.1.**
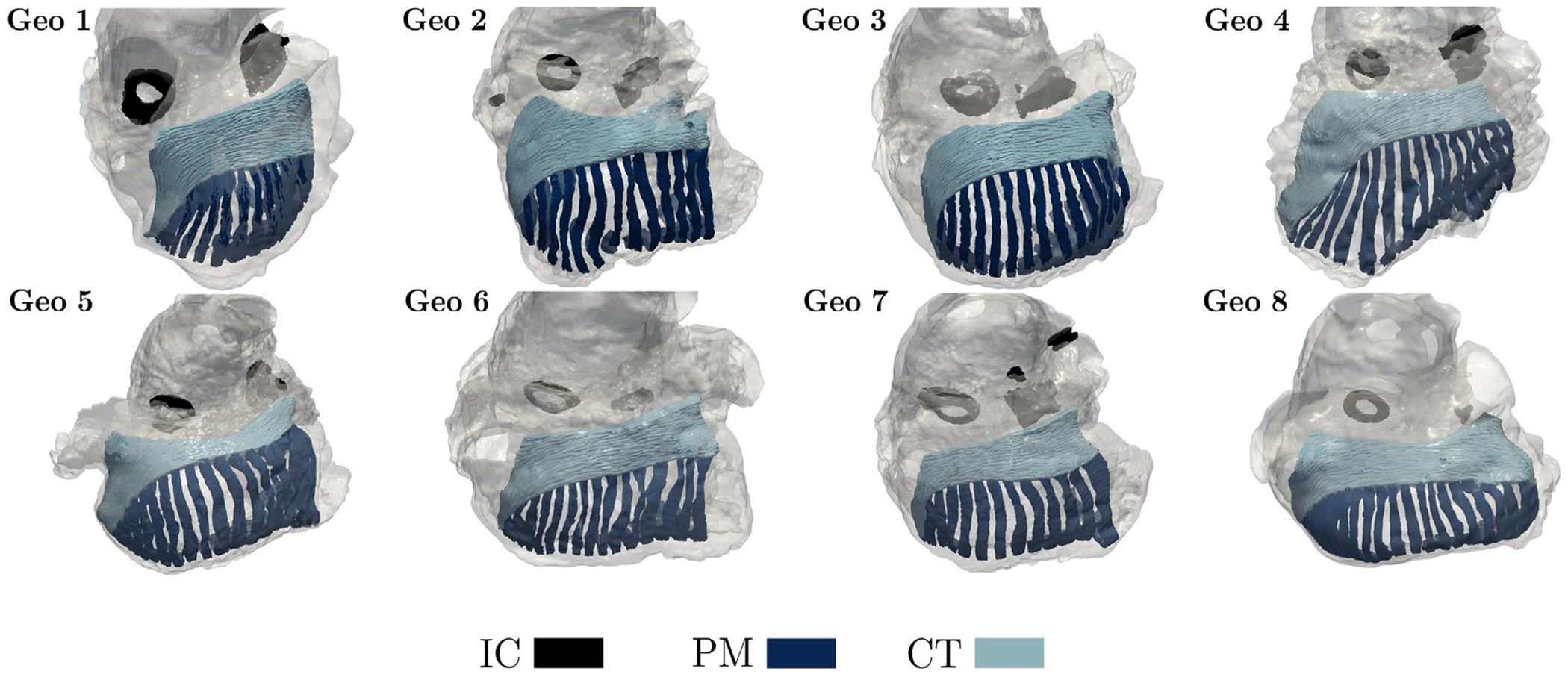
IC (black), PM (dark blue) and CT (light blue) bundles extraction performed by the bi-atrial LDRBM for DTMRI geometries. IC = Interatrial-Connections, PM = Pectinate Muscles, CT = Crista Terminalis.

The partitioning of the atria into their characteristic anatomical sub-regions is carried out according to Step 2 of the measurement procedure (see Section 3.2). This consists in applying the bundle subdivision of the novel bi-atrial LDRBM, across the eight DTMRI dataset geometries. Explicitly, according to the rules defined in Algorithm 1–3 (see Step 3 in Section 2.1), the LDRBM first extracts the inter-atrial connections (IC) and then selects the LA and RA bundles. The following anatomical areas, illustrated in Fig. 3(a,b) (see also Fig. C.1), are identified:

- IC: the Bachmann’s bundle connection (BB_IC_) in the central anterior region of the atria; the fossa ovalis rim connection (FO_IC_) across the atrial septum; the coronary sinus connection (CS_IC_) in the posterior wall (extracted only within Geo 2, with other geometries presenting an almost fused CS structure into LA); connections between RA and the sleeves of the right pulmonary vein in Geo 7.
- LA: the mitral valve (MV) vestibular region; the venous portion collecting left and right inferior and superior pulmonary veins (LIPV, LSPV, RIPV, RSPV); the right (RC) and left (LC) carina; the atrial appendage (LAA); the anterior septum (LAS) and Bachmann’s bundle (BB) in the anterior wall; the lateral wall (LLW) and atrial septum (LSW) in the lateral and septal regions of LA, respectively; the top (LAW_top_) and bottom (LAW_bot_) posterior wall; the atrial roof (LAR).
- RA: the tricuspid valve (TV) vestibular region; the venous portion composed of the inferior (ICV) and superior (SCV) caval veins; the roof wall between the orifices of caval veins, named the inter-caval bundle (IB); the coronary sinus musculature (CSM), joined to the adjacent LAW_bot_ and MV regions (extracted in all geometries except Geo 4); the atrial appendage (RAA); the posterior wall (RPW) and anterior wall (RAW) below ICV and SCV, respectively; the atrial septum (RAS); the crista terminalis (CT), which is clearly detectable on the sub-endocardium and extends from SCV to ICV curving to the right of ICV; a series of bundles known as pectinate muscles (PM) that fan out from CT towards TV; the lateral wall (RLW), overlapping CT and PM structures.

These anatomical regions (IC, LA and RA bundles) are clearly identified in all the eight morphologies by the LDRBM bundle subdivision procedure, see Step 3 in Section 2.1 and Algorithms 1–3. The input values of the parameters *τ_i_*, used to define the bundle dimensions throughout the atrial domain across the DTMRI dataset geometries, are listed in Tables C.4–C.6 of Appendix C. Fig. 3(c) reports the distribution (as box plots) of the bundle subdivision parameters *τ_i_* across the DTMRI dataset. Higher parameter standard deviation (SD) values for these parameters are observed in LAA, CSM, CT, and FO_IC_, indicating greater anatomical variability, while the remaining bundles exhibit lower SD revealing reduced morphological variation. Additional details are provided in appendix Fig. C.1 and Tables C.4–C.6.

#### 3.2.2. Assessment of atrial fiber orientations

**Fig. C.2.**
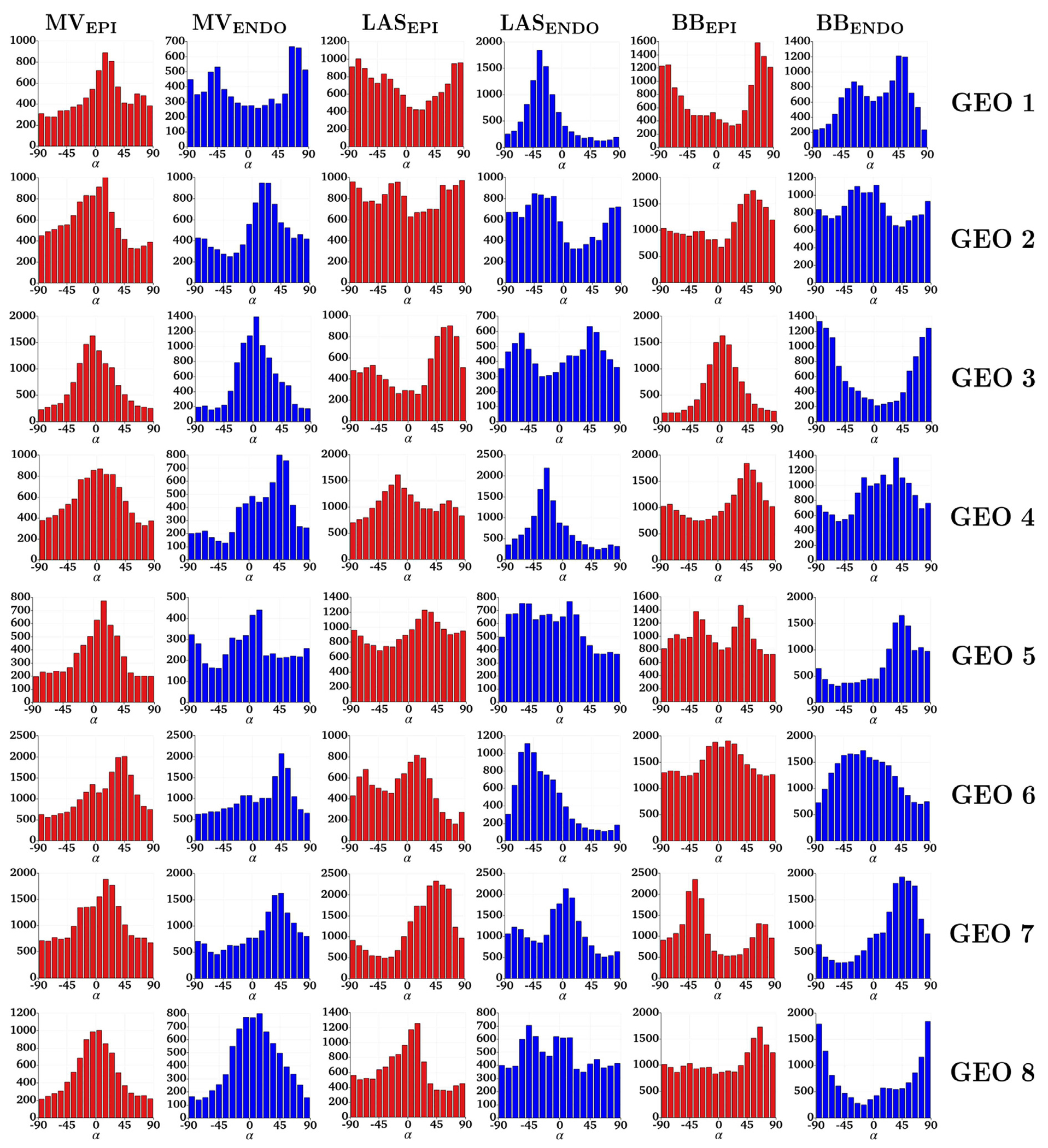
Histograms displaying the measured alpha angle (with respect to LDRBM coordinate axis system) across the eight DTMRI geometries in LA bundles (MV, LAS, BB) in the sub-epicardial (red) and sub-endocardial (blue) layers.

**Fig. C.7.**
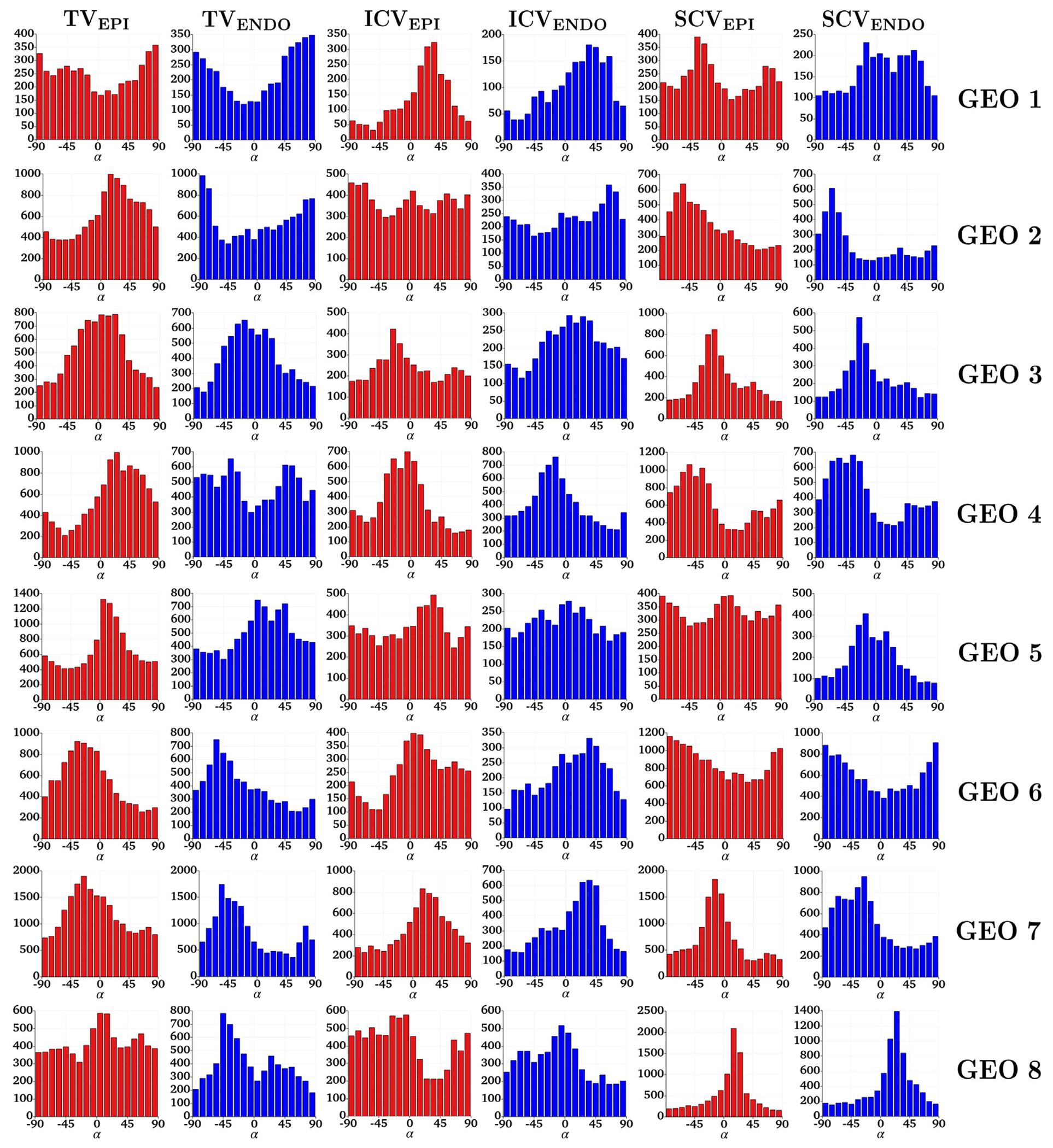
Histograms displaying the measured alpha angle (with respect to LDRBM coordinate axis system) across the eight DTMRI geometries in RA bundles (TV, ICV, SCV) in the sub-epicardial (red) and sub-endocardial (blue) layers.

**Fig. C.11.**
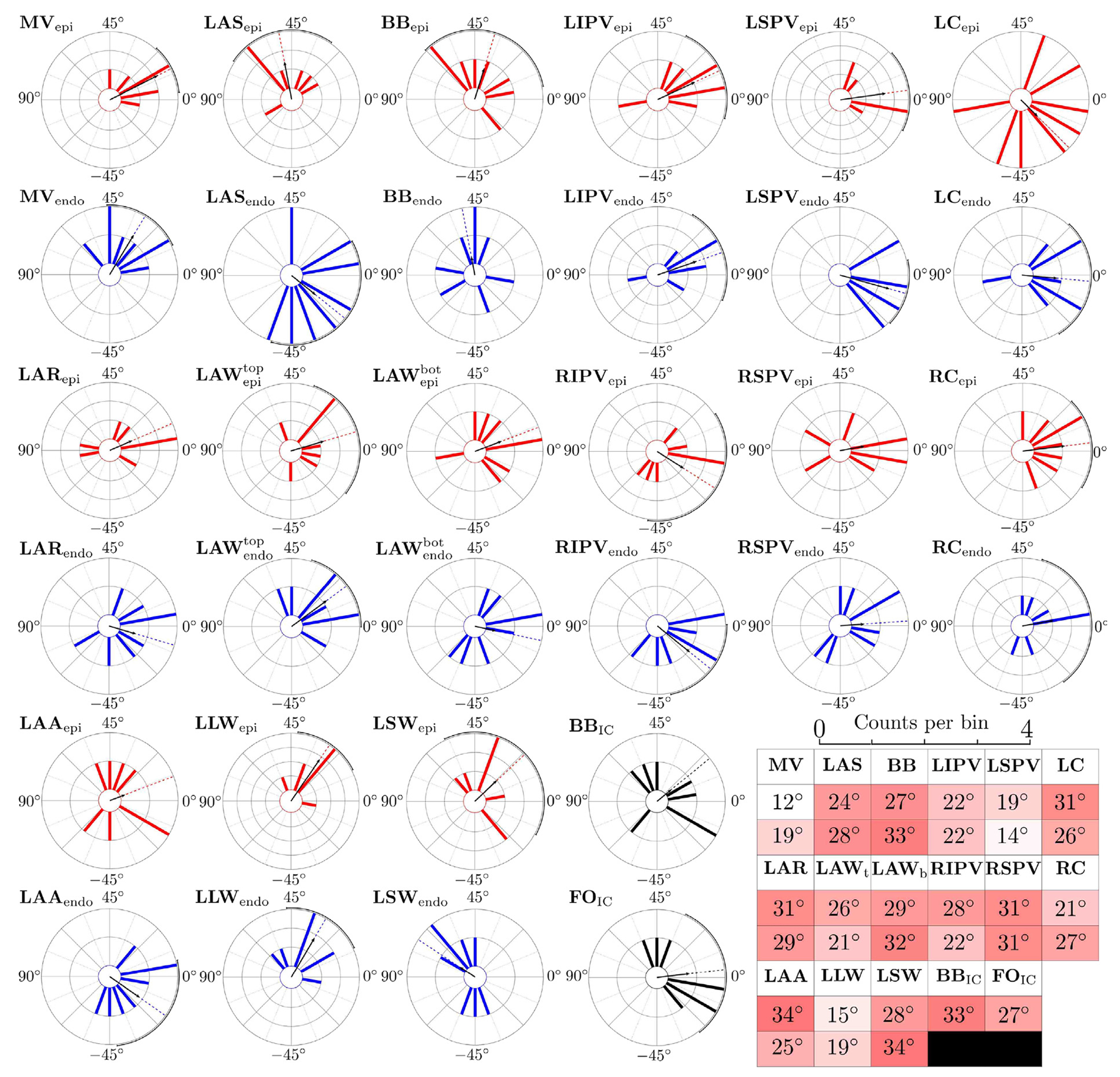
Circular histograms of dominant angle within LA bundles: sub-endocardium (blue), sub-epicardium (red) and inter-atrial connections (black) across eight DTMRI dataset geometries. Mean angle represented by dotted line. Resultant mean vector depicted in black, with length indicative of angular variance. Corresponding SD values for each bundle shown in table at bottom right corner, with SD color-coded on a scale from minimal (12°) to maximal (37°).

The quantitative characterization of fiber angles across the atrial wall is revealed according to Steps 4–6 of the measuring procedure (presented at the beginning of Section 3.2). Hereafter, we summarize the main findings for the DTMRI dataset [37]. Comprehensive details are available in Appendix C. Specifically, the histogram distributions of the measured fiber angles related to each atrial bundle are illustrated in Figs. C.2–C.6 for LA and in Figs. C.7–C.10 for RA. Moreover, the identified dominant fiber angles are reported in Tables 7 and 8. Finally, the result of the circular data analysis [85–87] (detailed in Appendix C.1) applied to the dominant fiber angles, is reported in Figs. C.11 and C.12. Resultant mean angles and the corresponding angular SD values are listed in Tables 7 and 8.

**Fig. 4.**
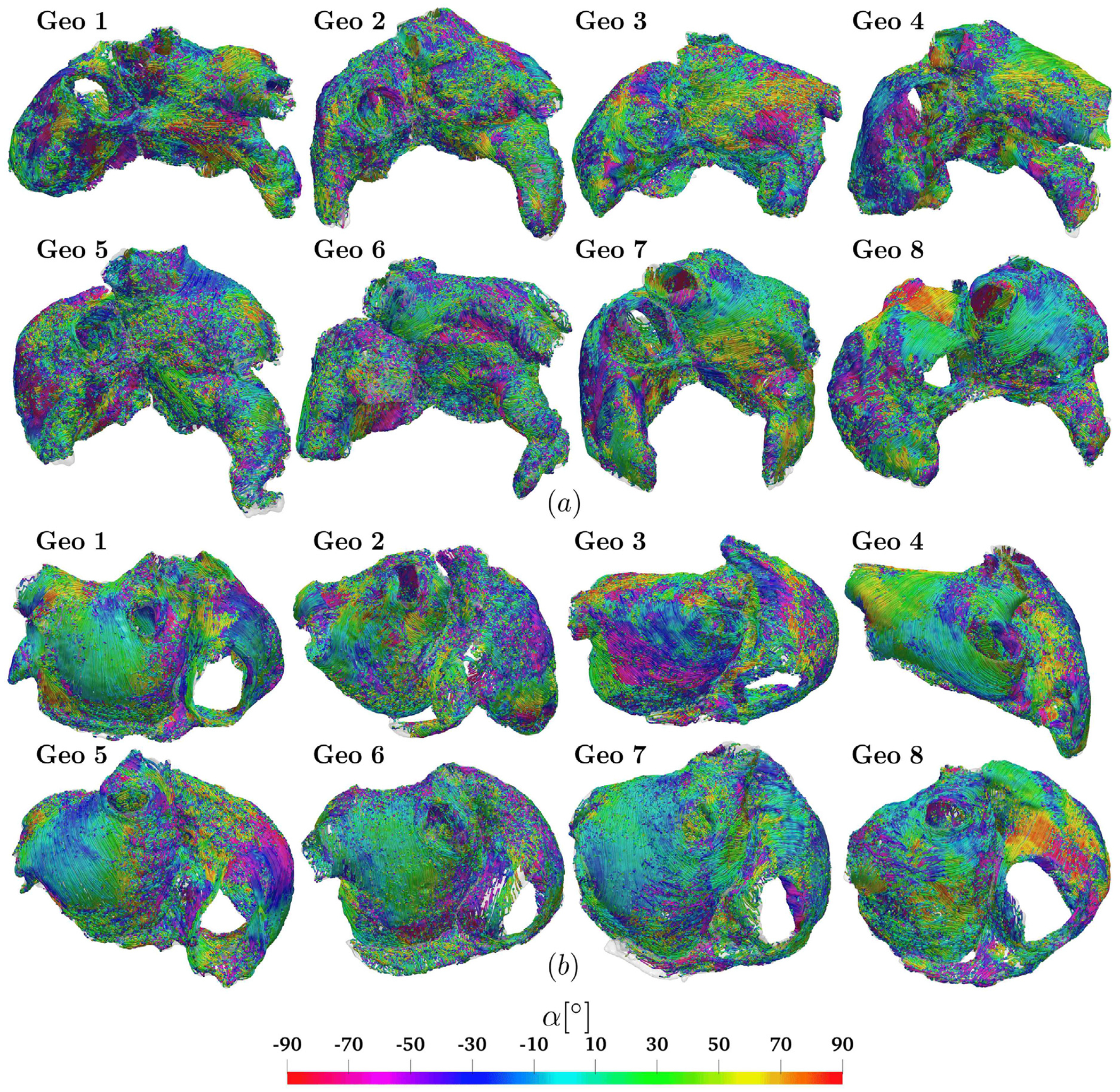
Measuring procedure applied to the eight geometries of DTMRI fiber dataset: streamlines represent DTMRI fiber directions, showing the measured angle *α* relative to LDRBM axis system. Anterior (a) and posterior (b) views.

Fig. 4 displays the global result of the measured fiber angles applied to the eight DTMRI geometries: streamlines show the fiber directions and the related angle α relative to the LDRBM local coordinate axis system. Fiber angle distribution reveals a significant transmural heterogeneity from the sub-endocardium (ENDO) to the sub-epicardium (EPI), across almost every atrial bundles, with fibers intersecting and traveling in different directions. Despite variations in exact orientations among specimens, the primary features of atrial fiber architecture are mostly preserved across the entire dataset, see Fig. 4.

Following [34,36], we describe the myofiber bundle structures as *circumferential* if the fiber orientations are roughly parallel to the MV/TV orifice, *longitudinal* if approximately perpendicular to MV/TV, and *oblique* if otherwise oriented relative to MV/TV.

Concerning LA, MV exhibits circumferential fibers in both EPI and ENDO layers across all the dataset. LIPV and LSPV display longitudinal-circular arrangements, except for Geo 3 which has predominantly oblique orientations. RIPV and RSPV show larger variability: RIPV has circumferential and oblique configurations, while RSPV includes also crossing fibers. LC and RC exhibit significant variability, especially in LC_EPI_, whereas LC_ENDO_ and RC have a longitudinal pattern. LAR reveals longitudinal orientations in six of the eight specimens. Geo 2–3 are unique, containing a mixture of oblique directions, especially in LAR_EPI_. LAW (both LAW_top_ and LAW_bot_) fibers were found to be posterior-to-anterior, with LAW_bot_ showing more pronounced transmural variation compared to LAW_top_. A pattern of overlapping fibers is consistently observed in the LA anterior wall: BB_IC_ presents circumferential fibers in half the dataset (Geo 1, 3, 7, 8) and oblique orientations in the remaining (Geo 2, 4, 5, 6); BB has oblique fibers more consistently in EPI compared to ENDO; LAS contains oblique EPI fibers in five specimens (Geo 2, 3, 4, 5, 7), transitioning to a longitudinal structure in ENDO; LAA shows a bimodal distribution of angles, with crossing directions from EPI to ENDO, significant variability in LAA_EPI_, and a preserved structure in LAA_ENDO_; LLW and LSW present well-established circumferential fibers, with LSW_ENDO_ featuring both oblique and longitudinal fibers; FO_IC_ fibers circularly run around its center in five of the eight specimens.

Regarding RA, TV presents circumferential fibers in EPI, similar to MV, but with a pronounced transmural variation to oblique in ENDO. SCV features consistent oblique directions. Conversely, ICV fibers are longitudinal-circular only in ENDO and oblique in EPI. IB_EPI_ shows circumferential orientations in four geometries (Geo 3, 4, 5, 8), longitudinal in two (Geo 2, 7), and oblique in the remaining (Geo 1, 6); whereas IB_ENDO_ has circumferential fibers in five specimens (Geo 1, 2, 4, 6, 8) and perpendicular/oblique in the others (Geo 3, 5, 7). RAA has fibers encircling the appendage, with more variability in ENDO compared to EPI. CSM features a well-preserved circumferential structure. RAS shows longitudinal orientations in ENDO, while EPI reveals significant variability with circumferential (Geo 3, 6), longitudinal (Geo 4, 7), and oblique (Geo 1, 2, 5, 8) directions. RPW unveils considerable transmurality, transitioning from longitudinal (EPI) to oblique (ENDO) fibers. RAW is characterized by longitudinal (Geo 4, 5, 6, 7) and oblique (Geo 1, 2, 3, 8) orientations. RLW exhibits varying oblique directions and includes the distinct CT and PM structures. CT is primarily oriented longitudinally extending from SCV to ICV and curving to the right of ICV (see Fig. C.15). According to the rules outlined in Algorithm 3, the LDRBM identified 17 ± 3 PM (see Table C.6) which run nearly perpendicularly to CT and follow the endocardial trabeculated structure of RA (see Fig. C.15).

### 3.3. Fiber generation

To verify the reliability of the bi-atrial LDRBM, we completely deploy the novel fiber generation model (see Section 2.1), to reconstruct all the eight DTMRI human atrial myofiber architectures. Furthermore, we compare the generated LDRBM fiber fields to the ground truth DTMRI data, investigating the differences in fiber orientations, across the entire DTMRI dataset, within both the volumetric sub-epicardial and sub-endocardial layers.

For the LDRBM bundle subdivision, we consider the parameters (reported in Tables C.4–C.6) used for the regional classification presented in Section 3.2.1. Additionally, the input angular values (listed in Tables 7 and 8) are chosen based on the observed dominant angles within the sub-epicardial and sub-endocardial layers) retrieved by the measurement procedure described in Section 3.2.2.

**Fig. 5.**
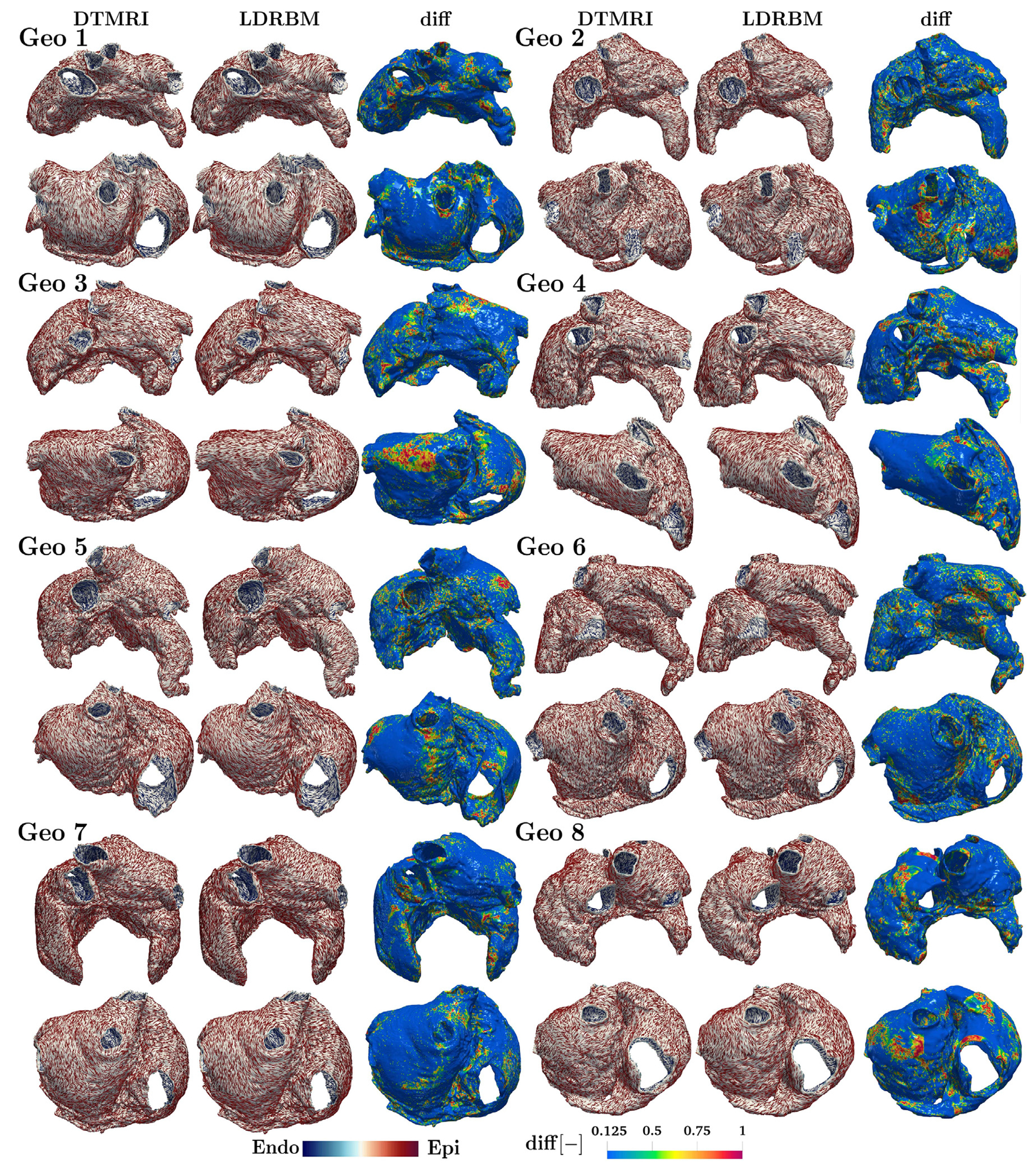
Comparison between the atrial LDRBM fibers and the DTMRI data, across eight DTMRI geometries. Glyph-rendered fiber vector fields are reported for each geometry (Geo 1–8), displayed in anterior (top) and posterior (bottom) views. The function diff, computed as $\mathrm{diff}(x)=1-\left|f_{\text{DTMRI}}(x)\cdot f_{\text{LDRBM}}(x)\right|$, highlights the differences between LDRBM and DTMRI fibers, see also Figs. C.13–C.14.

**Fig. C.13.**
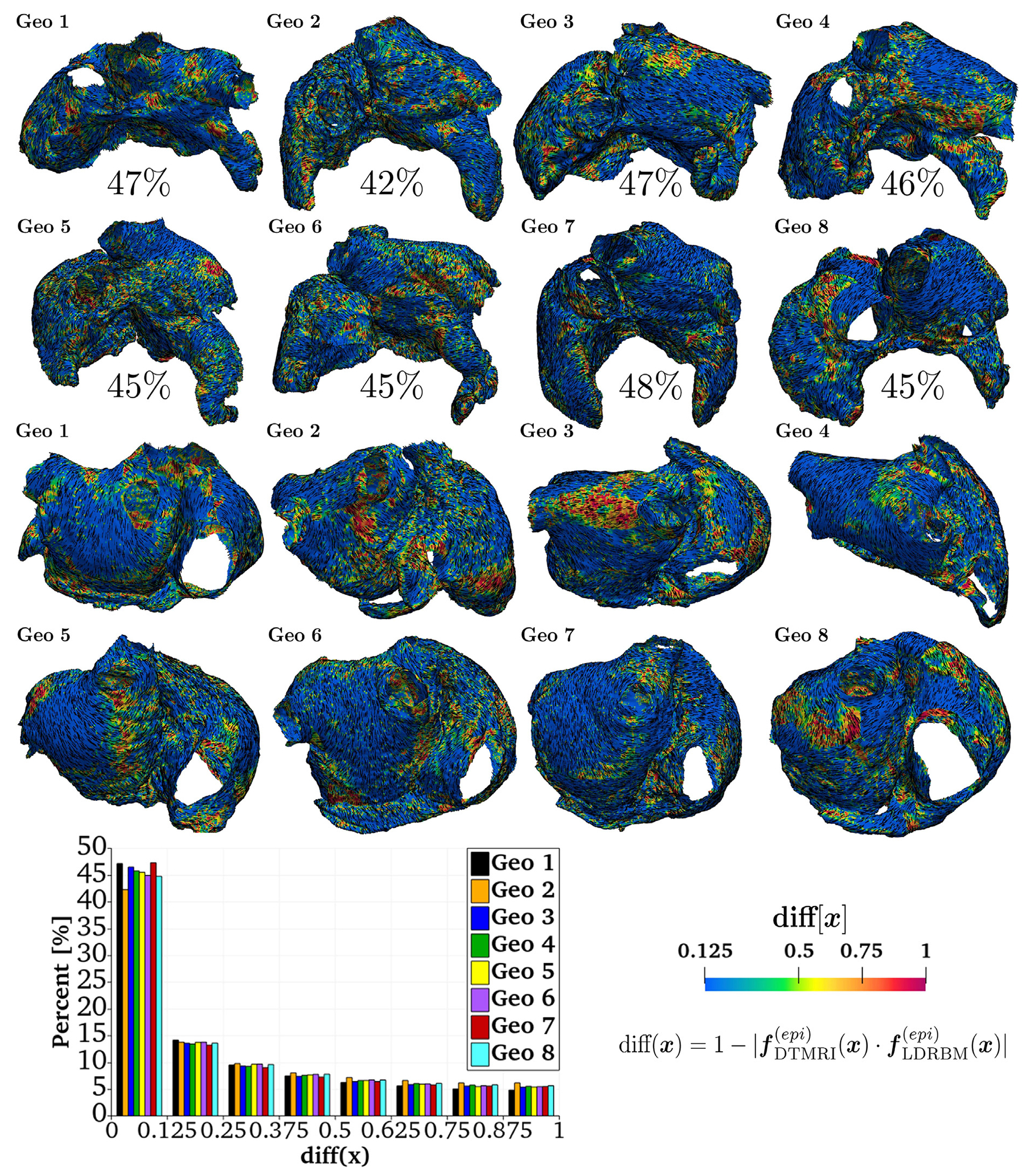
DTMRI sub-epicardial fiber architecture recreated by the LDRBM assigning in each bundle the dominant angle retrieved by the LDRBM-DTMRI measuring procedure. Glyph-rendered LDRBM fiber vector fields are reported (in black) for each geometry (Geo 1–8), displayed in anterior (top) and posterior (bottom) views. The function diff, computed as $\text{diff}(x)=1-\left|f_{\text{DTMRI}}^{\text{epi}}(x)\cdot f_{\text{LDRBM}}^{\text{epi}}(x)\right|.$ highlights the differences between LDRBM and DTMRI fibers. Histograms showing the fiber orientation agreement distribution of the diff function between LDRBM and DTMRI fibers with values per histogram bin of 0.125. The percentages close to diff ~ 0 indicate the amount of fibers in good agreement with respect to the total sub-epicardial fiber orientations.

Fig. 5 (second and fifth columns) shows the fiber orientations reconstructed using the bi-atrial LDRBM for all the eight DTMRI geometries (see also Figs. C.13–C.15). The LDRBM captures the complex fiber arrangement in almost all the principal anatomical atrial regions, generally reproducing the DTMRI fiber orientations (first and fourth columns of Fig. 5) with visible differences only in limited areas.

Fig. 5 (third and sixth columns) compares the generated LDRBM fibers with the ground truth DTMRI data, showing the fiber orientation differences evaluated using the function

(6) $$\mathrm{diff}(x)=1-\left|f_{\text{DTMRI}}(x)\cdot f_{\text{LDRBM}}(x)\right|,$$

where $f_{\text{DTMRI}}(x)$ and $f_{\text{LDRBM}}(x)$ are the vector fiber fields associated with DTMRI data and LDRBM, respectively. If $f_{\text{DTMRI}}$ and $f_{\text{LDRBM}}$ are parallel, diff =0, otherwise, when orthogonal, diff =1.

**Fig. C.14.**
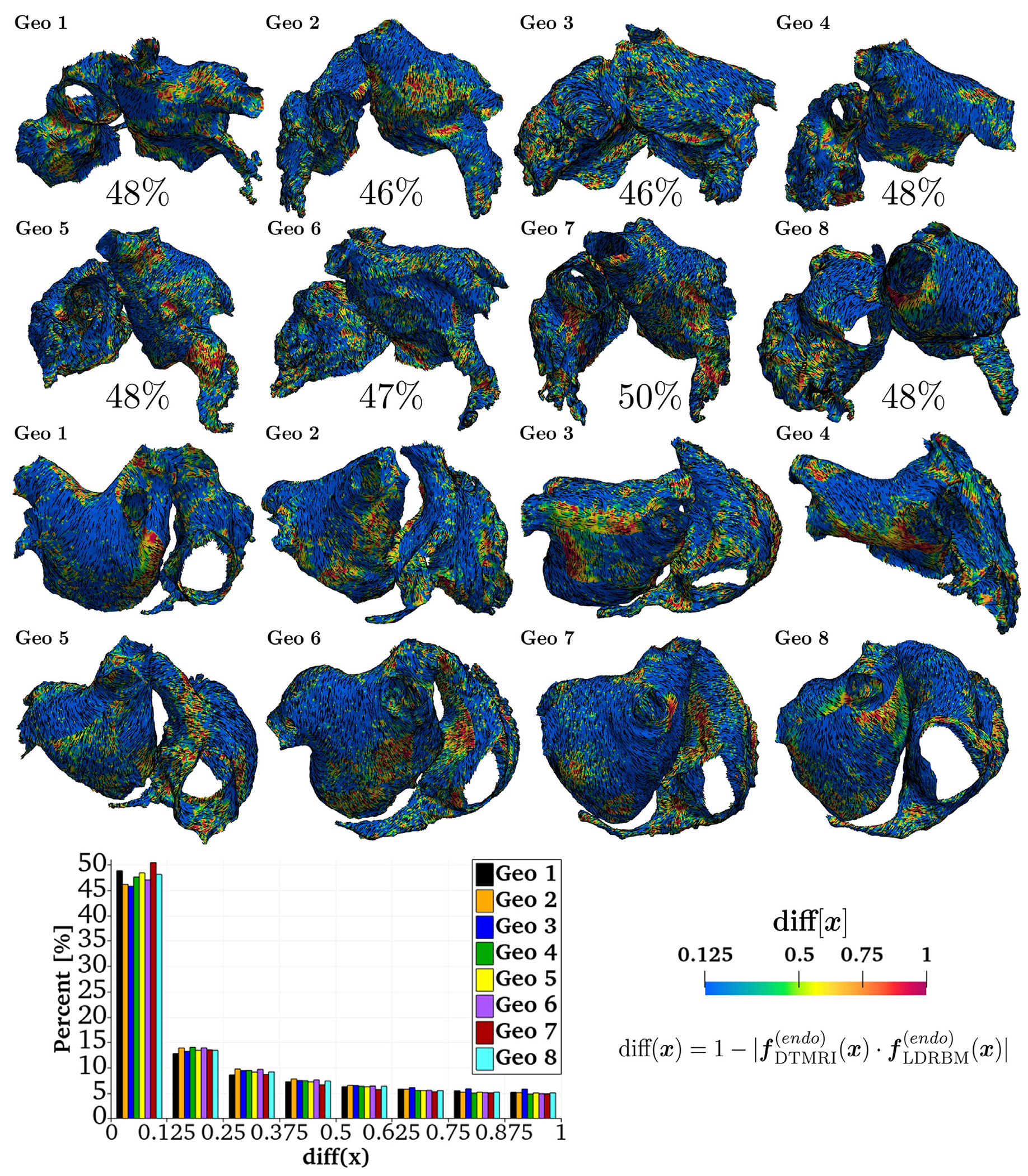
DTMRI sub-endocardial fiber architecture recreated by the LDRBM assigning in each bundle the dominant angle retrieved by the LDRBM-DTMRI measuring procedure. Glyph-rendered LDRBM fiber vector fields are reported (in black) for each geometry (Geo 1–8), displayed in anterior (top) and posterior (bottom) views. The function diff, computed as $\text{diff}(x)=1-\left|f_{\text{DTMRI}}^{\text{endo}}(x)\cdot f_{\text{LDRBM}}^{\text{endo}}(x)\right|,$ highlights the differences between LDRBM and DTMRI fibers. Histograms showing the fiber orientation agreement distribution of the diff function between LDRBM and DTMRI fibers with values per histogram bin of 0.125. The percentages close to diff ~ 0 indicate the amount of fibers in good agreement with respect to the total sub-endocardial fiber orientations.

**Fig. C.15.**
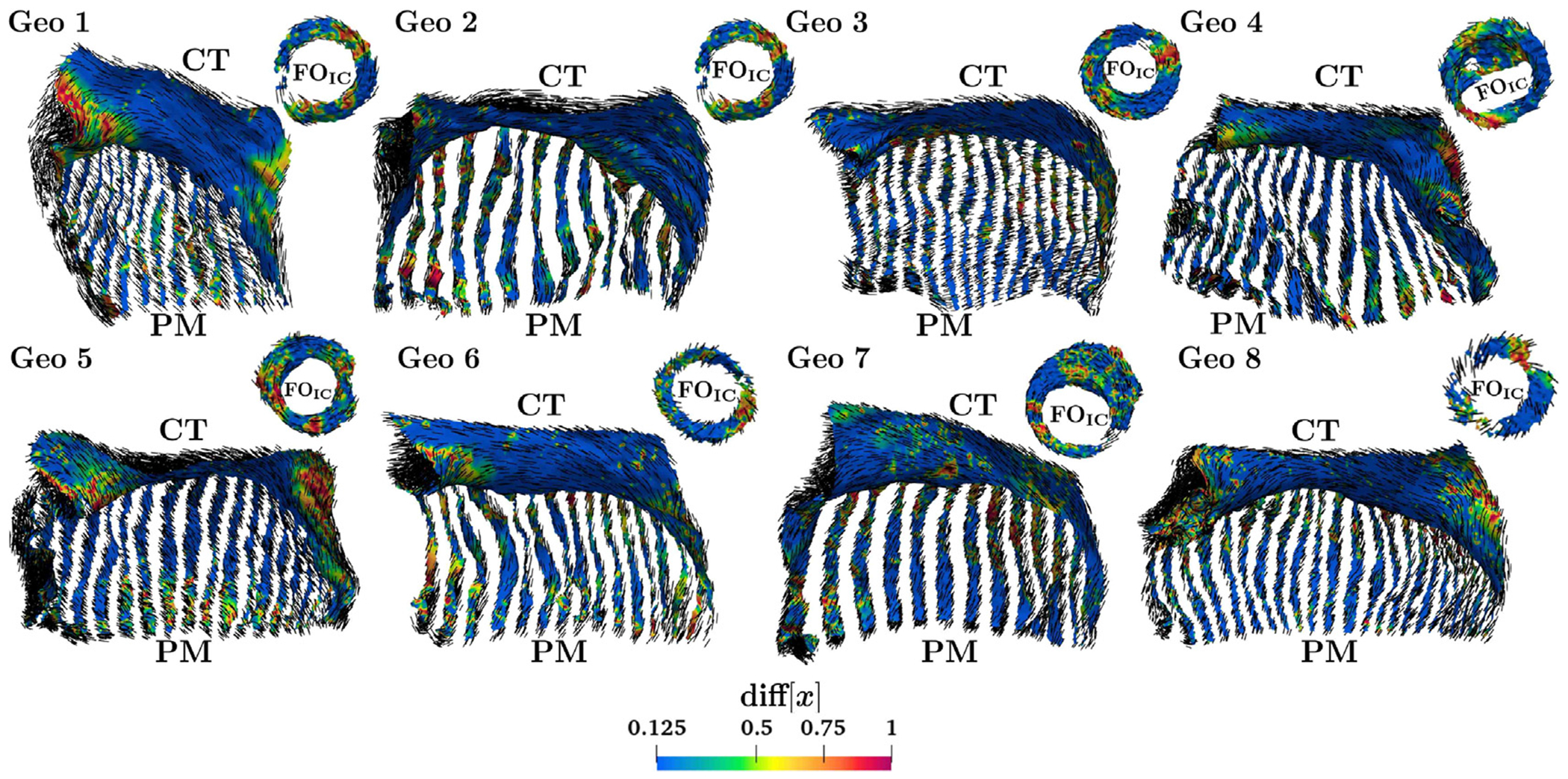
DTMRI fiber architecture recreated by the LDRBM for the Crista Terminalis (CT), Pectinate Muscles (PM) in RA and the Fossa Ovalis (FO_IC_) inter-atrial connection. Glyph-rendered LDRBM fiber vector fields are reported (in black) for each geometry (Geo 1–8). The function diff, computed as $\text{diff}(x)=1-\left|f_{\text{DTMRI}}(x)\cdot f_{\text{LDRBM}}(x)\right|,$ highlights the differences between LDRBM and DTMRI fibers: values close to diff ~ 0 indicate the amount of fibers in good agreement with respect to the DTMRI fiber orientations.

To further quantify the amount of matching between DTMRI and LDRBM fibers, we analyzed the distribution of diff(***x***) function values across the different morphologies (see Figs. C.13–C.15). This analysis was performed in the sub-endocardial and sub-epicardial volumetric layers, as identified by the transmurality function defined in Eq. (5). The overall percentage of fibers in good agreement with DTMRI, relative to the total fiber orientations, was 46 ± 1% across all geometries, ranging from 43% (Geo 2) to 48% (Geo 7). Furthermore, in the sub-epicardium, the agreement percentage was 46 ± 2%, with values ranging from 42% (Geo 2) to 48% (Geo 7) (see Fig. C.13). In the sub-endocardium, the agreement percentage was 48±1%, ranging from 46% (Geo 2–3) to 50% (Geo 7) (see Fig. C.14–C.15).

### 3.4. Electrophysiology simulations

To assess the impact of using LDRBM and DTMRI fiber architectures on electric signal propagation, we perform two types of EP simulations employing the Eikonal-diffusion model (detailed in Appendix B) for each DTMRI geometry: one with DTMRI fiber data and the other with LDRBM fibers. To quantify the deviations in activation times (AT), resulting from the different fiber architectures, we employ the following quantitative indicators evaluated on the same computational domain, using either LDRBM or DTMRI fibers:

- the total activation time (TAT), defined as
$${TAT}_{i}=\underset{x}{\max}\left[u^{i}(x)\right],\;i=\text{DTMRI, LDRBM,}$$
  where $u^{DTMRI}(x)$ and $u^{LDRBM}(x)$ are the numerical local AT retrieved by EP simulations endowed with DTMRI and LDRBM fibers, respectively;
- the error between TAT of LDRBM and DTMRI, as
$${err}_{\text{TAT}}=\left|{TAT}_{LDRBM}-{TAT}_{\text{DTMRI}}\right|;$$
- the maximal local AT error between LDRBM and DTMRI, namely $\underset{x}{max}\;\left[{err}_{AT}(x)\right]$ where ${err}_{AT}(x)$ is defined as
$${err}_{AT}(x)=\left|u^{LDRBM}(x)-u^{DTMRI}(x)\right|;$$
- the volumetric compatibility index error ${Vol}_{>10\%}$, indicating the percentage of atrial volume where the local AT exceeds 10% of error
(7) $${\text{Vol}}_{>10\text{\%}}[\text{\%}]=\frac{N_{\mathrm{tot}}-N_{<10\text{\%}}}{N_{\mathrm{tot}}}100,$$
  where $N_{\text{tot}}$ and $N_{<10\%}$ are the total number of EP solution degree of freedom (d.o.f) and the number of d.o.f where AT do not exceed 10% of error, respectively.

**Fig. 6.**
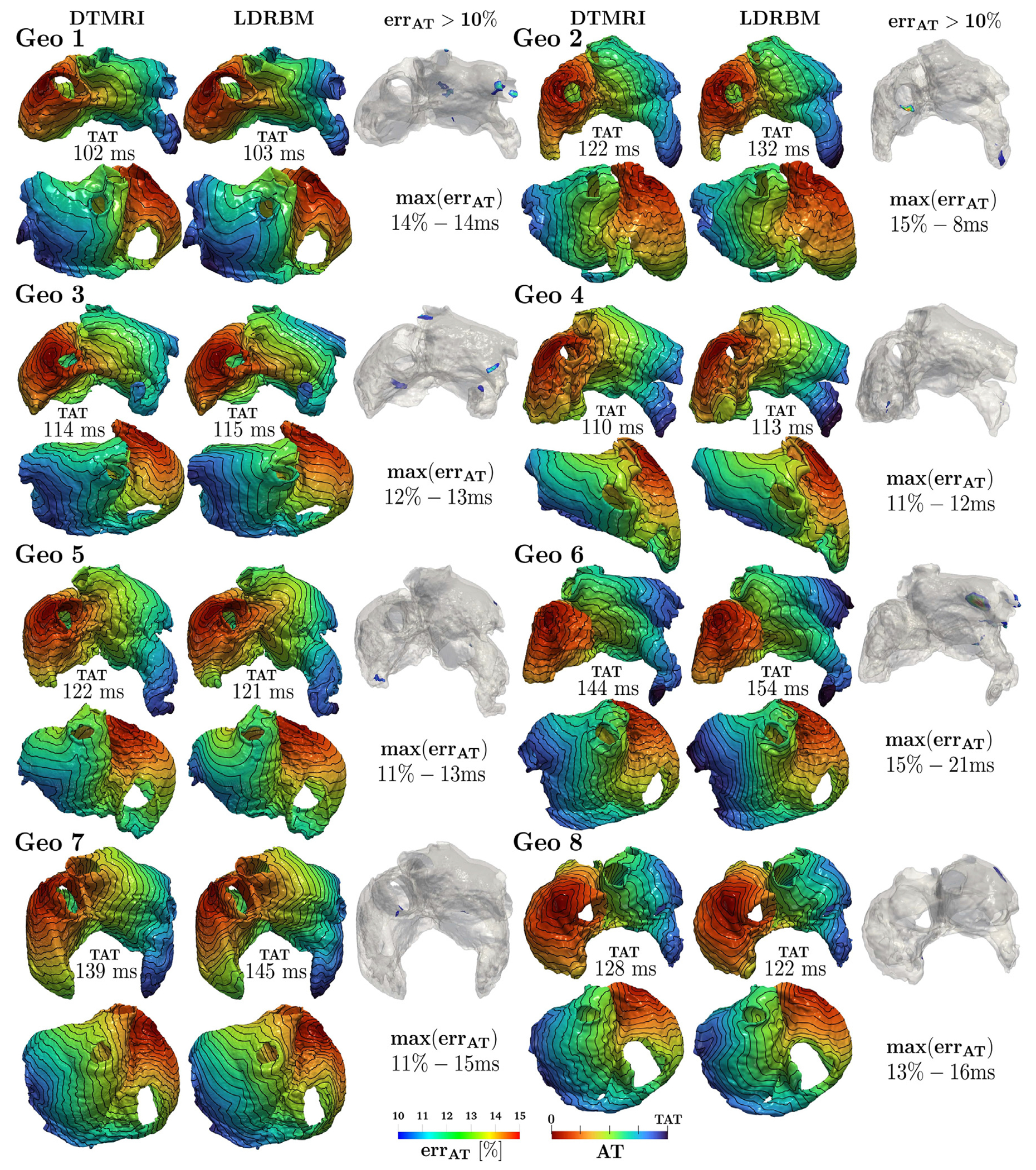
Comparison between the activation times (AT) derived from the numerical EP simulations endowed with LDRBM and DTMRI fibers, across the DTMRI dataset. Each geometry’s anterior and posterior AT views are displayed, with isochrones spaced 10 ms apart. TAT is the total activation time obtained with DTMRI and LDRBM fibers; max(err_AT_) denotes the maximal local AT (relative–absolute) error, while err_AT_ > 10% highlights volumetric regions where AT relative difference exceeds 10% of error. Further details are reported in Table 2. See also appendix Fig. C.16 which illustrates the complete AT map differences between EP simulations using LDRBM and DTMRI fibers across the DTMRI dataset.

**Fig. C.16.**
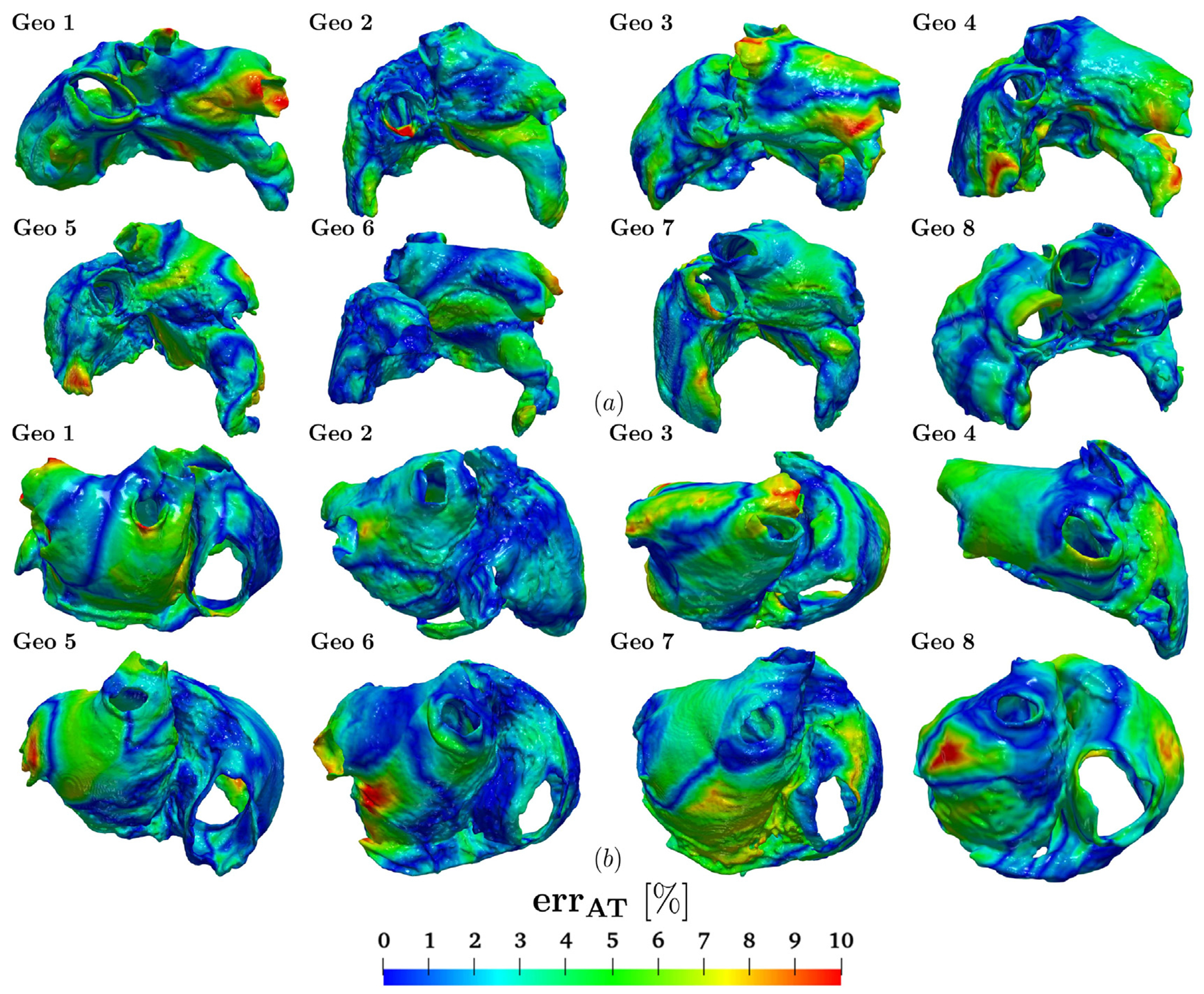
Comparison between the activation times (AT) derived from the numerical EP simulations endowed with LDRBM and DTMRI fibers, across the DTMRI dataset. err_AT_ highlights the relative differences in the local AT maps. Further details are reported in Table 2, see also Fig. 6.

Fig. 6 reports the comparison between AT coming from EP simulations obtained on the same computational domain with either LDRBM and DTMRI fibers, across all the dataset (see also Table 2 and appendix Fig. C.16). The simulations predict total activation times ${TAT}_{i}(i=$ DTMRI, LDRBM) that are perfectly compatible, with an absolute error ranging from 1 ms to 10 ms, corresponding to a relative one between 1% to 9% and a mean absolute/relative error, across all the eight geometries, of 5±4ms/4±3%. The local activation maps feature also a very similar morphology with marginal discrepancies only in limited regions. The latter is confirmed by the absolute/relative maximal local AT error from 8ms/11% to 21ms/15%, with a mean value of 14±4ms/13±2%. Bundle-regions where the relative local AT error exceeds the 10% are almost equally observed in LA and RA: 5 LA (RPV, LPV, MV, LAW, LAA) and 4 RA (SCV, TV, RAA, SCV) bundles (see Fig. 6 and also appendix Fig. C.16). However, the volumetric compatibility index error ${Vol}_{>10\%}$ is well below the 1% across the entire dataset. This indicates that activation maps arising from LDRBM and DTMRI fibers are compatible within 99% of the entire bi-atrial volume for all the specimens.

### 3.5. Comparison with state-of-the-art atrial fiber models

**Fig. C.17.**
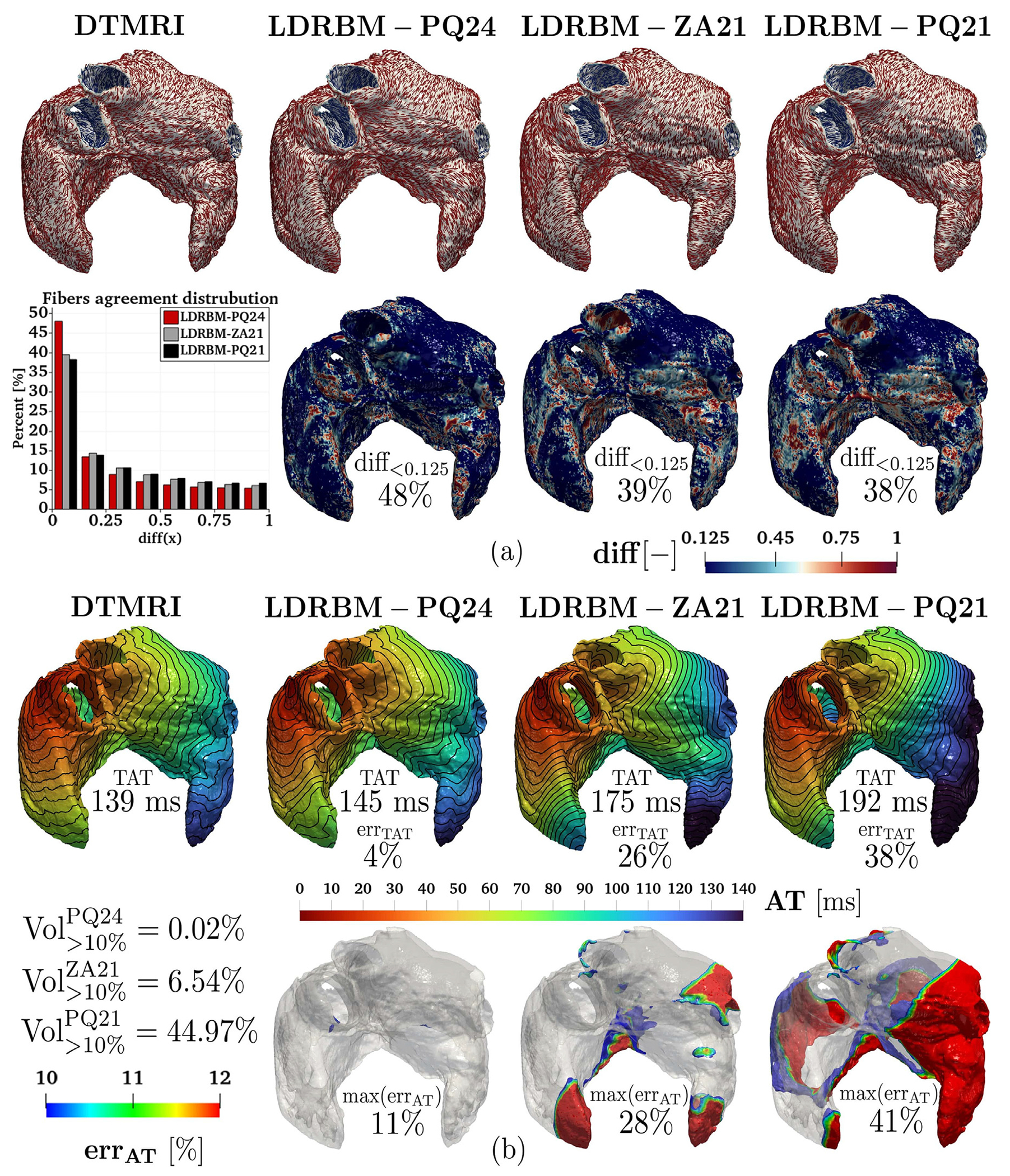
Comparative analysis of different LDRBM models (LDRBM-PQ24, LDRBM-ZA21 [52,67] and LDRBM-PQ21 [20]) against DTMRI fibers (Geo 7 analyzed): (a-top) Glyph-rendered representations showcase the fiber architectures among models; (a-bottom) fiber disparities relative to DTMRI data, calculated as $\text{diff}(x)=1-\left|f_{\text{DTMRI}}(x)\cdot f_{i}(x)\right|,$ with i=LDRBM-PQ24 on the left, i=LDRBM-ZA21 in the center, and i=LDRBM−PQ21 on the right; (b-top) Activation maps (with isochrones spaced 10 ms apart) from EP simulations produced using different fiber models TAT is the total activation time and $err_{TAT}$ denotes the TAT relative error; (b-bottom) $err_{AT}$ highlights volumetric regions where local AT relative difference exceeds 10% error and $\max\left(err_{AT}\right)$ signifies the maximal relative local AT error, while $Vol_{\left(>10\text{\%}\right)}^{i}$ (with i = PQ24, ZA21 and PQ21) indicates the percentage of atrial volume where the local AT exceeds 10% error.

The results obtained by our novel bi-atrial LDRBM, both for the fiber directions and the EP simulations, were compared with other fiber generation models. Specifically, we considered the Universal Atrial Coordinates (UAC) ABM, originally proposed in [59] and then extended to account for volumetric meshes in [51,88]. Additionally, we examined the first atrial LDRBM introduced in [20], which was subsequently refined in [52,67] with the inclusion of a distinction between epicardial and endocardial layers. To differentiate between the LDRBM, we name hereafter the first LDRBM as LDRBM-PQ21 [20], the refined version LDRBM-ZA21 [67], while the one presented in this work as LDRBM-PQ24. All the comparisons are performed on the same mesh, coming from Geo 7. The UAC input fibers is the one originally derived by the authors in [59], corresponding to the morphology Geo 7 of the DTMRI dataset [37] (see also https://zenodo.org/records/3764917). We refer the reader to [20,51,52,67,88] for further details about UAC, LDRBM-PQ21 and LDRBM-ZA21 fiber models, respectively. Additional information and details are reported in the appendix Figs. C.17 and C.18.

**Fig. 7.**
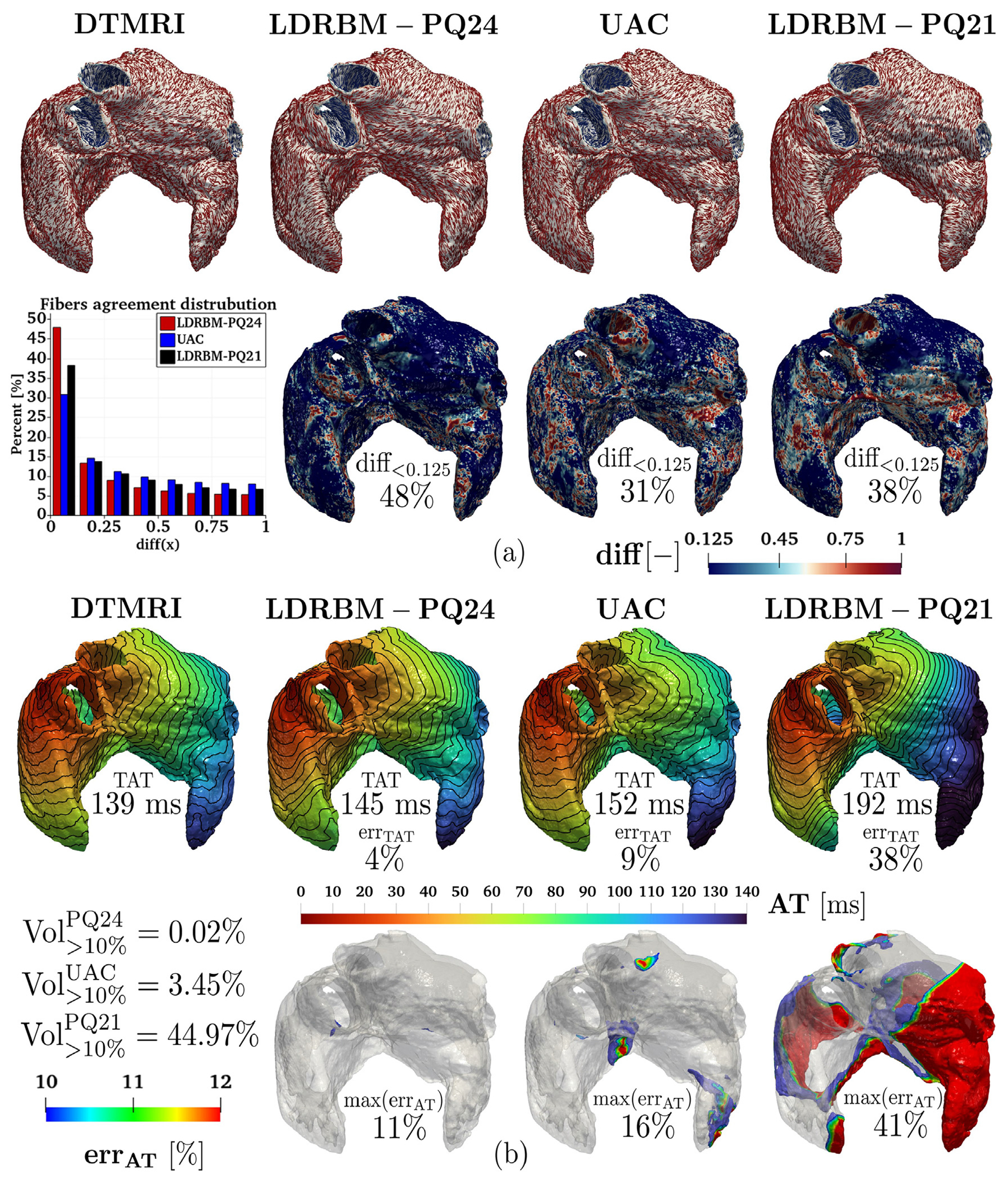
Comparative analysis of different fiber models (LDRBM-PQ24, UAC [59] and LDRBM-PQ21 [20]) against DTMRI fibers (Geo 7 analyzed): (a-top) Glyph-rendered representations showcase the fiber architectures among models; (a-bottom) fiber disparities relative to DTMRI data, calculated as $\mathrm{diff}(x)=1-\left|f_{\text{DTMRI}}(x)\cdot f_{i}(x)\right|$, with i=LDRBM-PQ24 on the left, i=UAC in the center, and i=LDRBM-PQ21 on the right; (b-top) Activation maps (with isochrones spaced 10 ms apart) from EP simulations produced using different fiber models TAT is the total activation time and err_TAT_ denotes the TAT relative error; (b-bottom) err_AT_ highlights volumetric regions where local AT relative difference exceeds 10% error and $max\left({err}_{AT}\right)$ signifies the maximal relative local AT error, while ${Vol}_{\left(>10\%\right)}^{i}$ (withi=PQ24,UAC and PQ21) indicates the percentage of atrial volume where the local AT exceeds 10% error.

Fig. 7(a) showcases the fiber comparison results. The architecture generated by LDRBM-PQ24 is in excellent agreement with DTMRI data, reproducing nearly the same fiber orientations in all the different atrial bundles. In contrast, both UAC and LDRBM-PQ21 exhibit several discrepancies compared to DTMRI fibers. The latter arise mostly in LSPV, RSPV, LAA, RAA, RAS and LAS for UAC, and in LAA, RAA, RAS, LAS, BB, CT and PM for LDRBM-PQ21. Furthermore, both LA and RA septal junctions and inter-atrial connections are completely misrepresented in LDRBM-PQ21.

The fiber discrepancies are quantified by evaluating for each methodology the function (6) with respect to the DTMRI atrial fiber architecture, see Fig. 7(a). The percentage of fibers in good agreement with respect to DTMRI data are 48%, 31% and 38% for LDRBM-PQ24, UAC and LDRBM-PQ21, respectively (see Figs. 7(a) and C.18(c)).

To quantify the AT discrepancies predicted by the different fiber architectures (LDRBM-PQ24, UAC, LDRBM-PQ21 and DTMRI), we perform four EP simulations using the Eikonal-diffusion model (detailed in Appendix B). We evaluate the TAT/AT errors (with respect to DTMRI) and the volumetric compatibility index (7), following the same analysis presented in Section 3.4.

Fig. 7(b) reports the comparison among the different EP simulations predicted by DTMRI, LDRBM-PQ24, UAC, and LDRBM-PQ21 fibers. The results are also resumed in Table 3. Both AT and the propagation morphology between the simulations with LDRBM-PQ24 and DTMRI fibers are in very good agreement, with discrepancies exceeding 14 ms (i.e., 10% of error with respect to AT produced by DTMRI fibers) only in restricted zones of SCV and TV, see Fig. 7(b). These correspond to 0.02% of the total myocardial volume. Conversely, AT predicted by UAC shows higher discrepancies in several LA and RA bundles (RAA, LAR, TV, RAS, LAS and MV), corresponding to 3.45% of the atrial volume. Finally, the simulation with LDRBM-PQ21 produces remarkably different values for both TAT (with 38% error with respect to DTMRI fibers) and local AT, with differences exceeding 10% error extending over half of the LA and RA volume, see Fig. 7(b) and Table 3.

Appendix Fig. C.17 illustrates the EP/fiber comparison analysis, focusing exclusively on the three LDRBMs: LDRBM-PQ21 [20], LDRBM-ZA21 [67], and the newly developed LDRBM-PQ24. LDRBM-ZA21 demonstrates improved fiber orientation accuracy compared to LDRBM-PQ21, achieving 39% alignment with DTMRI data. However, LDRBM-PQ24 outperforms LDRBM-ZA21, benefiting from enhanced transmurality that enables distinct fiber orientations in the sub-endocardial and sub-epicardial layers, see Fig. C.17(a). Despite the improvements with respect to LDRBM-PQ21, AT predicted by EP simulations using LDRBM-ZA21 fibers exhibit up to 26% error compared to DTMRI-derived fibers. This discrepancy is particularly notable in several left and right atrial bundles (RAA, LAA, LPV, TV, RAS, LAS, and MV), affecting 6.54% of the atrial volume, see Fig. C.17(b).

## 4. Discussion

In this work, we presented a novel LDRBM modeling approach (see Section 2.1) for prescribing myofiber orientations and providing robust regional annotation in bi-atrial morphologies of any complexity through a highly automated framework. The robustness of our method was verified using eight highly detailed bi-atrial geometries, processed from the original DTMRI human atrial fiber dataset presented in [37]. Furthermore, we developed a systematic measurement procedure, leveraging the LDRBM reference axis system, to assess local myocardial fiber angles across the atrial wall in geometries embedded with experimental DTMRI data (see Section 3.2). According to this measurement procedure, we established a rule for determining the dominant fiber angle within the sub-endocardial and sub-epicardial layers of each atrial bundle. Next, we validated the atrial LDRBM by quantitatively reproducing all the eight DTMRI fiber architectures (see Section 3.3). Moreover, we demonstrated that numerical EP simulations of electrical wave propagation, using both LDRBM and DTMRI fibers on the same geometries, exhibit excellent agreement (see Section 3.4). Finally, by comparing our modeling approach with state-of-the-art atrial fiber generation models [20,59,67] against ground-truth DTMRI fibers, we proved that the bi-atrial LDRBM outperforms current methodologies used for prescribing myofiber orientations in ADT (see Section 3.5). Therefore, our novel rule-based modeling approach establishes a new standard to prescribe fibers in ADT, with the potential to significantly enhance their precision.

Compared to previous atrial fiber computational approaches [48,51,55,56,58–60,62–65] our methodology, specifically designed for bi-atrial anatomies, preserves the distinctive simplicity of the LDRBM framework [20], unlike many of the existing challenging-to-implement methodologies [51,60,64]. Our method requires the definition of Laplace problems with suitable Dirichlet boundary conditions prescribed on common boundary sets, such as endocardium, epicardium, atrioventricular valves and veins rings. A dedicated pipeline for boundary label assignment, tailored specifically for the proposed bi-atrial LDRBM, has been developed (see Appendix A). The latter ensures that the boundary label assignment is accomplished through a streamlined pipeline, ensuring precision, reproducibility, and high usability. The only user interaction involves selecting four landmarks (LAA, RAA, FO and CSM apices), which are straightforward to identify on the atrial geometry, as well as setting the threshold for anatomical bundle subdivisions. These features are consistent with the methods described in [20,52,67,68]. We underline that for the orifice labeling in DTMRI geometries, where morphologies are highly complex, we performed manual tagging of the valve and vein orifices. However, we emphasize that this orifice-valve manual labeling is not essential for the applicability of bi-atrial LDRBM. Our pipeline is fully compatible with other orifice tagging processes described in the literature, including those by Piersanti et al. [20] and Zheng-Azzolin et al. [52,67]. Notably, the method in [67] uses local curvature analysis combined with a Poisson problem to automatically extract valve and vein orifices. Adding this capability to our labeling pipeline will enable fully automated orifice-boundaries identification. As for the threshold parameters used to define bundle dimensions across the atrial geometry, we observed that only four of these parameters – those corresponding to LAA, CSM, CT and FO_IC_ (see Fig. 3) – exhibit significant variability. Therefore, only these thresholds typically require adjustment to accommodate patient-specific geometries. Nevertheless, we are actively working on techniques to automate the estimation of the entire set of parameters for patient-specific geometries.

Our fiber model not only preserves the natural flexibility and morphological adaptability of LDRBMs proposed so far [20,52,67,68,71], but also significantly improves them by accounting for an heterogeneous bundle-specific fiber architecture. By leveraging on newly introduced inter/intra-atrial distances, consistently defined for LA and RA (see Section 2.1), our model extensively refine the bi-atrial regional classification. With dedicated fiber definitions in each bundle for both sub-epicardial and sub-endocardial layers, the bi-atrial LDRBM constructs and modulates a volumetric two-layer myofiber field, resulting in an exceptionally detailed fiber architecture with unprecedented results relative to the existing literature. In comparison to existing atrial LDRBMs [20,52,67,68], the proposed bi-atrial LDRBM was specifically designed to account for bi-atrial geometry, while also incorporating the capability to prescribe specific inter-atrial connection bundles, such as the fossa ovalis. The LDRBMs described in Piersanti et al. [20] and Rossi et al. [68] were tailored for standalone RA and/or LA geometries. Conversely, Azzolin and Zheng et al. [52,67] utilized a different rule-based methodology based on Wachter et al. [63], employing a boolean mesh operations to define inter-atrial connections and to model RA pectinate muscles. Our methodology distinguishes itself by treating bi-atrial morphologies – including also RA pectinate muscles and inter-atrial connections – adopting entirely a Laplace–Dirichlet framework. Additionally, our bi-atrial LDRBM substantially improves the bundle identification in both RA and LA, nearly doubling the number of atrial bundles identified in prior studies [20,52,67,68]. Specifically, our approach identifies 15 atrial bundles in LA and 12 in RA, as compared to 4 (for LA) and 7 (for RA) bundles identified by Piersanti et al. [20]. Furthermore, while Azzolin et al. [52] is built upon the LDRBM proposed by Piersanti et al. [20] and updated in Zheng et al. [67], which improved the identification of the left and right atrial appendages and introduced a distinction between epicardial and endocardial layers, our LDRBM extends also these advancements. Not only it enables the differentiation of epicardial and endocardial volumetric sub-layers, but also incorporates specific fiber orientations for each layer. This includes setting different dominant angles for fibers in each endocardial and epicardial bundle layer, further enhancing the granularity and accuracy of our model. Finally, for the first time, we demonstrated that a RBM is capable of reproducing the DTMRI atrial muscular architectures. We showed that numerical EP tissue activations predicted by LDRBM fibers are almost identical to the one produced by the ground truth fibers (see Sections 3.3–3.4). This work is pioneering in validating an atrial fiber model against orientations obtained from DTMRI data.

The atrial wall has been qualitatively observed in previous studies, including histo-anatomical ex-vivo observations [34–36] and DTMRI tractography analysis [37], to exhibit a bilayer bundle structure characterized by fibers crossing and running in various directions. However, quantitative measurement of myocardial architecture properties, such as fiber angles, requires the definition of a coordinate system (or systems) in the atria that is reproducible across subjects with various atrial morphologies. This has been previously proposed for the ventricles [89,90], but it is particularly challenging for the atria due to their complex shape [57]. Here, we established a systematic measurement procedure expoiting the LDRBM reference system to quantitatively characterize the DTMRI architectures, uncovering the local atrial fiber angle and enabling the identification of the dominant angle within the sub-endocardial and sub-epicardial layers of each atrial bundle (see Section 3.2). Compared to the previous attempt proposed in [59], which measured the DTMRI human atrial fiber dataset [37] globally on highly smoothed endocardial and epicardial surfaces, our analysis allowed us to see through the entire atrial wall. This enabled to perform quantitative measurement and characterization of the local transmural fiber distribution throughout all the atrial bundles (see Section 3.2.2). We exploited the semi-automatic LDRBM regional classification algorithm to identify 27 distinct bundles from DTMRI data (see Section 3.2.1). Then, we measured the local atrial fiber angles within the LDRBM axis system. The results demonstrates a bilaminar (sub-epicardial and sub-endocardial) architecture with fiber orientations revealing a significant transmural heterogeneity across almost every bundles. Despite variations among specimens, the primary features of the fibers are mostly preserved across the atrial dataset (see Section 3.2.2).

We constructed a set of eight distinct fiber architectures, along with a mean fiber configuration, blending together the bi-atrial LDRBM and DTMRI measurements. Specifically, we completely deployed the LDRBM fiber generation pipeline (see Section 2.1), leveraging on DTMRI-LDRBM measurement information (Section 3.2), to reconstruct all the eight DTMRI human atrial myofiber architectures. We verified that LDRBM fiber-replicas accurately capture the complex arrangement in nearly all anatomical regions, generally reproducing the same fiber orientations with visible differences only in limited areas. The overall percentage of LDRBM fibers in good agreement with DTMRI data (relative to the total fiber orientations) was 46 ± 2% in the sub-epicardium and 48±1% in the sub-endocardium (see Section 3.3). While our LDRBM successfully reproduces the global DTMRI fiber architecture, achieving higher agreement across the remaining fiber field is challenging, primarily due to the high level of local fiber disarray observed in DTMRI data for certain atrial bundles (e.g., LAA, RAA, CSM, and the bottom LAW) and a limitation in the current treatment of fiber transmurality in our bi-atrial LDRBM. The fiber disarray may result from noise in the measurements, particularly in morphologically complex regions such as the LAA/RAA or the CSM, which is nearly fused with surrounding structures. Additionally, a possible fiber remodeling due to pathophysiological conditions in some regions, such as the LAR, could contribute to these misalignments. Furthermore, our bi-atrial LDRBM prescribes two distinct but constant fiber orientations for each bundle in the sub-endocardial and sub-epicardial layers, based on the dominant angles observed in DTMRI data. We are actively working on addressing these limitations by developing methods to estimate and model local fiber disarray and improve the transmural treatment of fibers for each specific bundle.

EP simulations using both LDRBM and DTMRI fibers predicted a highly compatible AT, with a mean TAT error of 5 ± 4 ms. The activation morphology patterns were nearly indistinguishable, with a maximal local AT error averaging 14 ± 4 ms (see Section 3.4). The volumetric compatibility index error, Vol_>10%_ < 1%, indicates 99% compatibility between EP simulations using LDRBM and DTMRI fibers (see Table 2). It is important to note that our EP simulations account for variations in atrial wall thickness. Unlike other existing fiber pipelines [20,48,57], our methodology allows the development of ADT that incorporates fiber architecture with specific transmural fiber orientations for each bundle in thickness-variable simulations. The significance of wall thickness and fibrosis distribution in arrhythmia mechanisms has been demonstrated in [91]. Variations in fiber modeling related to thickness may also impact EP activation patterns. Therefore, incorporating bundle-specific fiber transmurality into ADT could be crucial in studying rhythm disorders [25,92–96].

Compared to a recent work [68] that examined various LA fiber methodologies, this study is the first to compare state-of-the-art RBM [20,67] and ABM [59] on patient-specific bi-atrial anatomy against DTMRI ground truth fibers. We proved that our LDRBM outperforms current state-of-the-art atrial fiber generation models (namely UAC [59], PQ21 [20] and ZA21 [67]) in representing DTMRI fiber data. The percentage of fibers in good agreement was 48% for LDRBM, compared to 31% for UAC, 38% for PQ21 and 39% for ZA21 (see Section 3.5). Additionally, when comparing EP simulations, we found that AT and propagation morphology using LDRBM and DTMRI fibers are highly consistent (with 0.02% of total atrial volume discrepancy). Conversely, UAC, PQ21 and ZA21 exhibited larger differences (3.45%, 44.97% and 6.45%, respectively). These results underscore the exceptional accuracy of LDRBM and highlight the critical role of incorporating biophysically detailed fiber orientations in EP simulations. In contrast to [59,97], claiming that the choice of fiber fields has minimal impact in sinus rithm pacing, we discovered that AT differences can range from to 24 ms to 78 ms when comparing different fiber models to the ground truth fiber data (see Section 3.5). In fibrillatory dynamics, the fiber field can have a dramatic effect on predicting reentrant regions, as noted in [22,51]. However, significant gaps remain in our understanding of fiber directions and their role in the genesis and progression of arrhythmias.

In conclusion, we believe that this work stands out as a unique instance in the literature where atrial fiber architectures are modeled with an exceptional level of biophysical detail. To the best of our knowledge, this is the pioneering work of validating a rule-based atrial model against fiber orientations obtained from DTMRI data. However, we underline that the proposed bi-atrial LDRBM is not inherently patient-specific, primarily because atrial fiber orientations cannot currently be acquired in-vivo as part of standard clinical practice. Should this capability become available in the future, our LDRBM framework is already designed to accommodate such information, as it establishes a clear rule for determining the dominant fiber angle within each atrial bundle. Additionally, our approach offers a robust fiber-framework for the rapid development of personalized model cohorts accounting for detailed fiber anatomy and facilitates bi-atrial EP simulations. Ultimately, this study marks a significant advancement in building physics-based ADT, conceivably enhancing their precision for personalized risk assessment and potentially leading to better diagnostic and therapeutic strategies for cardiac diseases.

## Figures and Tables

**Table 1:** Boundary data chosen in the Laplace problem (1) for the inter-atrial distances (BIA) ξ,ϕ and the intra-atrial distances (LA/RA) $\psi_{i}$ with i=ab,v,r,w,t,a,aa,s,ct. The values $\chi_{a},\chi_{b}$ are set in order to evaluate specific inter/intra-atrial distances between boundary partitions $\Gamma_{a}$ and $\Gamma_{b}$. Refer to Step 2 for further details.

Type	χ	$\chi_{a}$	$\Gamma_{a}$	$\chi_{b}$	$\Gamma_{b}$
BIA	ξ	1	$\Gamma_{\ell a}$	−1	$\Gamma_{\mathrm{ra}}$
	ϕ	1 −1	$\Gamma_{\ell a}$ $\Gamma_{\mathrm{ra}}$	0	$\Gamma_{\mathrm{epi}}$
LA	$\Psi_{ab}$	2 0	$\Gamma_{\mathrm{rpv}}$ $\Gamma_{\ell pv}$	1 −1	$\Gamma_{\mathrm{mv}}$ $\Gamma_{\ell aa}$
	$\Psi_{v}$	1	$\Gamma_{\mathrm{rpv}}$	0	$\Gamma_{\ell pv}$
	$\Psi_{r}$	1	$\Gamma_{\mathrm{mv}}$	0	$\Gamma_{\ell pv}\cup \Gamma_{\mathrm{rpv}}\cup \Gamma_{\mathrm{epi}}^{top,\ell a}\cup \Gamma_{\mathrm{epi}}^{top,\ell p}$
	$\Psi_{w}$	1	$\Gamma_{rspu}\cup \Gamma_{\ell spv}\cup \Gamma_{\mathrm{mv}}^{\mathrm{ant}}$	−1	$\Gamma_{\text{ripu}}\cup \Gamma_{\text{eipv}}\cup \Gamma_{\mathrm{mv}}^{\text{post}}$
	$\Psi_{t}$	1	$\Gamma_{\mathrm{mv}}$	0	$\Gamma_{\mathrm{epi}}^{\text{top},\ell a}\cup \Gamma_{\mathrm{epi}}^{\text{top,}\ell_{p}}$
	$\Psi_{a}$	1	$\Gamma_{\ell a}^{\text{sept}}$	0	$\Gamma_{\ell a}^{\text{lat}}$
	$\Psi_{aa}$	1	$\Gamma_{\mathrm{fo}}$	−2	$\Gamma_{\ell aa}$
	$\Psi_{s}$	1 0	$\Gamma_{mv}^{\mathrm{ant}}$ $\Gamma_{\mathrm{epi}}^{top,\ell a}\cup \Gamma_{\ell a}^{\mathrm{sept}}\cup \Gamma_{\ell a}^{\mathrm{lat}}$	−0.5 −1	$\Gamma_{epi}^{top,\ell p}$ $\Gamma_{\mathrm{mv}}^{\mathrm{post}}$
RA	$\Psi_{ab}$	2 0	$\Gamma_{\mathrm{icv}}$ $\Gamma_{\mathrm{scv}}$	1 −2	$\Gamma_{\mathrm{tv}}$ $\Gamma_{\mathrm{raa}}\cup \Gamma_{\mathrm{csm}}$
	$\Psi_{v}$	1	$\Gamma_{ivc}$	0	$\Gamma_{\text{svc}}$
	$\Psi_{r}$	1	$\Gamma_{\mathrm{tv}}$	0	$\Gamma_{ivc}\cup \Gamma_{svc}\cup \Gamma_{\mathrm{epi}}^{top,ra}$
	$\Psi_{w}$	1 −1	$\Gamma_{tb}^{\mathrm{sept}}$ $\Gamma_{\mathrm{tv}}^{\text{lat}}$	0	$\Gamma_{\mathrm{epi}}^{\text{top},a}$
	$\Psi_{t}$	1	$\Gamma_{\mathrm{tv}}$	0	$\Gamma_{\mathrm{epi}}^{\text{top},a}$
	$\Psi_{a}$	1 −1	$\Gamma_{\mathrm{ra}}^{\mathrm{ant}}\cup \Gamma_{svc}$ $\Gamma_{\mathrm{ra}}^{\mathrm{post}}\cup \Gamma_{ive}$	2	$\Gamma_{\text{raa}}$
	$\Psi_{aa}$	1 −2	$\Gamma_{ivc}\cup \Gamma_{\mathrm{ra}}^{\mathrm{post}}$ $\Gamma_{\mathrm{raa}}$	0	$\Gamma_{svc}$
	$\Psi_{ct}$	1	$\Gamma_{\mathrm{ra}}^{\text{sept}}\cup \Gamma_{\mathrm{ra}}^{\text{ant}}\cup \Gamma_{\mathrm{ra}}^{\text{post}}$	−1	$\Gamma_{\mathrm{ra}}^{\mathrm{lat}}$

**Table 2:** Differences in EP simulations between LDRBM and DTMRI fiber architectures across eight DTMRI geometries: ${\mathrm{TAT}}_{i}$ is the total activation time (TAT) obtained with DTMRI (i= DTMRI) and LDRBM (i= LDRBM) fibers; err_TAT_ denotes the TAT absolute (in ms) and relative (in %) error; max $\left({err}_{AT}\right.$) signifies the maximal absolute/relative local activation time (AT) error; Bundle $\left({err}_{AT}>10\%\right)$ lists regions where the local AT exceeds 10% error; ${Vol}_{\left(>10\%\right)}$ is the volumetric error index indicating the percentage of atrial volume where the local AT exceeds 10% error.

Geometry	TAT_DTMRI_	TAT_LDRBM_	err_TAT_	max(err_AT_)	Bundle(err_AT_ > 10%)	Vol_>10%_
GEO 1	102 ms	103 ms	1 ms/1%	14 ms/14%	RPV/LPV/LAW	0.40%
GEO 2	122 ms	132 ms	10 ms/8%	8 ms/15%	SCV/LAA	0.24%
GEO 3	114 ms	115 ms	1 ms/1%	13 ms/12%	RPV/LPV/LAA/TV	0.30%
GEO 4	110 ms	113 ms	3 ms/6%	12 ms /11%	RAA	0.01%
GEO 5	122 ms	121 ms	1 ms/1%	13 ms/11%	RAA/LPV/MV	0.04%
GEO 6	144 ms	154 ms	10 ms/9%	21 ms/15%	LAW/RPV/CSM	0.82%
GEO 7	139 ms	145 ms	6 ms/4%	15 ms/11%	SCV/MV	0.02%
GEO 8	128 ms	122 ms	6 ms/5%	16 ms/13%	LAW	0.10%

**Table 3:** Differences in EP simulations produced by different atrial fiber models (LDRBM-PQ24, UAC [59], LDRBM-ZA21 [67] and LDRBM-PQ21 [20]) against ground truth DTMRI fiber data (Geo 7 analyzed). TAT is the total activation time; err_TAT_ denotes the TAT absolute (in ms) and relative (in %) error; max(err_AT_) signifies the maximal local activation time (AT) absolute/relative error; Bundle(err_AT_ > 10%) lists regions where the local AT exceeds 10% error; Vol_(*>*10%)_ is the volumetric error index, indicating the percentage of atrial volume where the local AT exceeds 10% error.

Type	TAT	err_TAT_	max(err_AT_)	Bundle(err_AT_ > 10%)	Vol_(>10%)_
DTMRI	139 ms	–	–	–	–
LDRBM-PQ24	145 ms	6 ms/4%	15 ms/11%	SCV/MV	0.02%
UAC	152 ms	13 ms/9%	24 ms/16%	RAA/LAR/TV/RAS/LAS/MV	3.45%
LDRBM-ZA21	175 ms	36 ms/26%	39 ms/28%	RAA/LAA/LPV/TV/RAS/LAS/MV	6.54%
LDRBM-PQ21	192 ms	53 ms/38%	78 ms/41%	almost half LA and RA	44.97%

**Fig. A.1.**
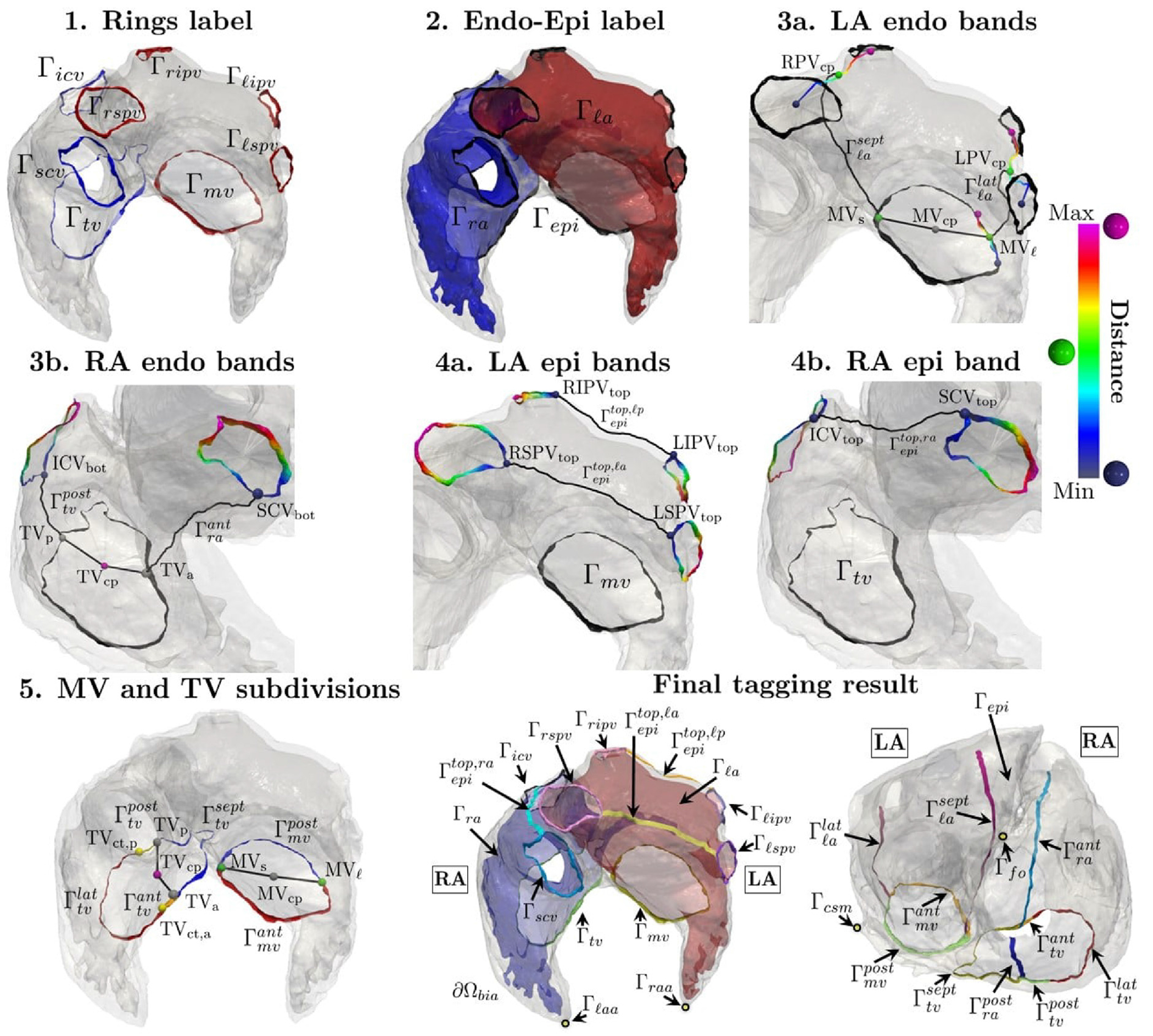
Atrial surface labeling procedure performed to impose the boundary conditions for the atrial LDRBM (presented in Section 2.1): (1.) crafting the LA (in red) and RA (in blue) rings; (2.) extracting LA (red), RA (blue) endocardium and atrial epicardium (gray); (3a., 3b.) LA and RA point selection procedures applied to compute shortest distance path employed to perform the endocardial bands labeling; (4a., 4b.) LA and RA point selection procedures applied to compute shortest distance path employed to perform the epicardial bands labeling; (5) annulus splitting (anterior, posterior, lateral and septal) for MV and TV. On the right, the final result of the labeling procedure applied to Geo 7 of the DTMRI dataset.

**Fig. C.3.**
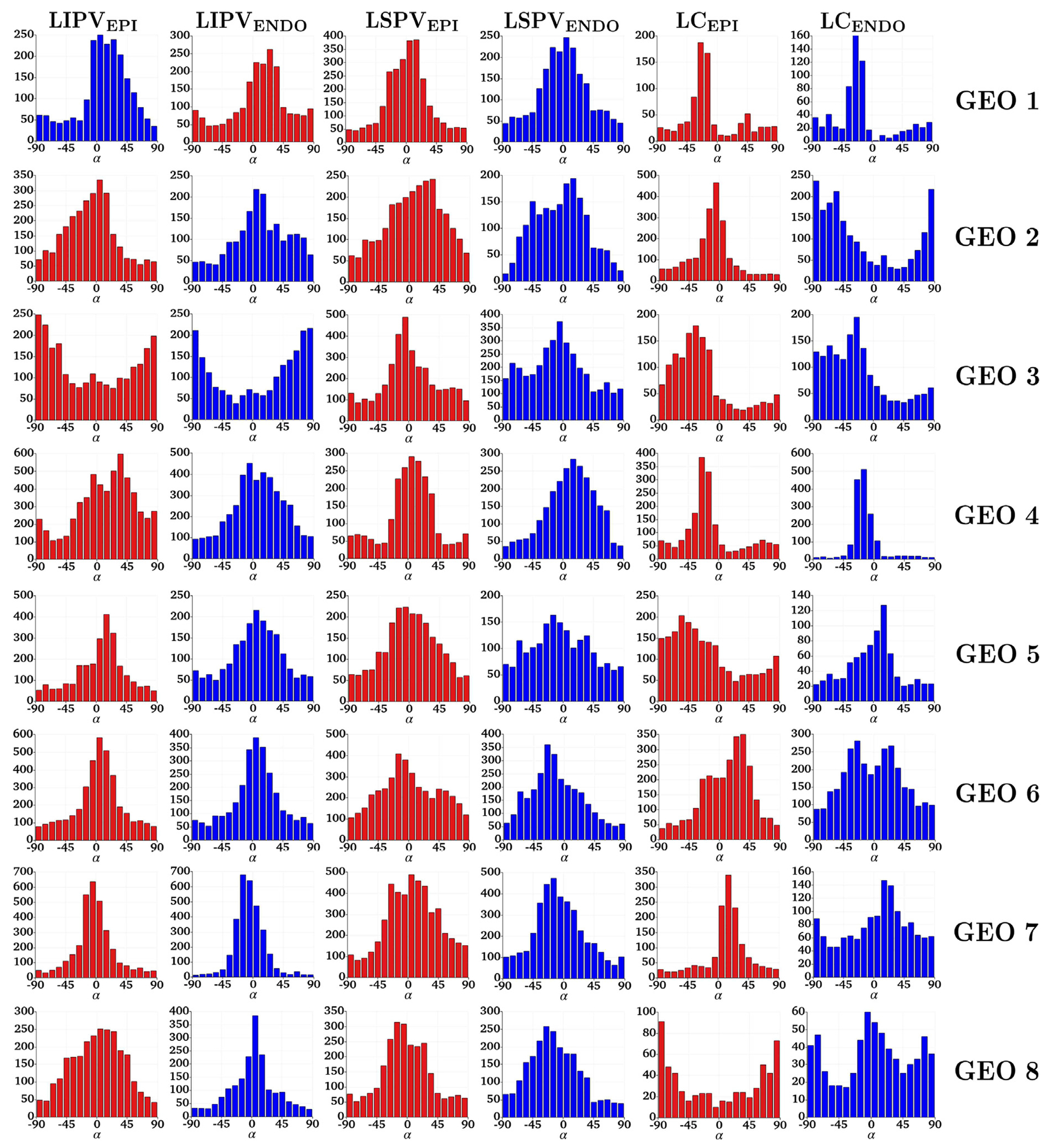
Histograms displaying the measured alpha angle (with respect to LDRBM coordinate axis system) across the eight DTMRI geometries in LA bundles (LIPV, LSPV, LC) in the sub-epicardial (red) and sub-endocardial (blue) layers.

**Fig. C.4.**
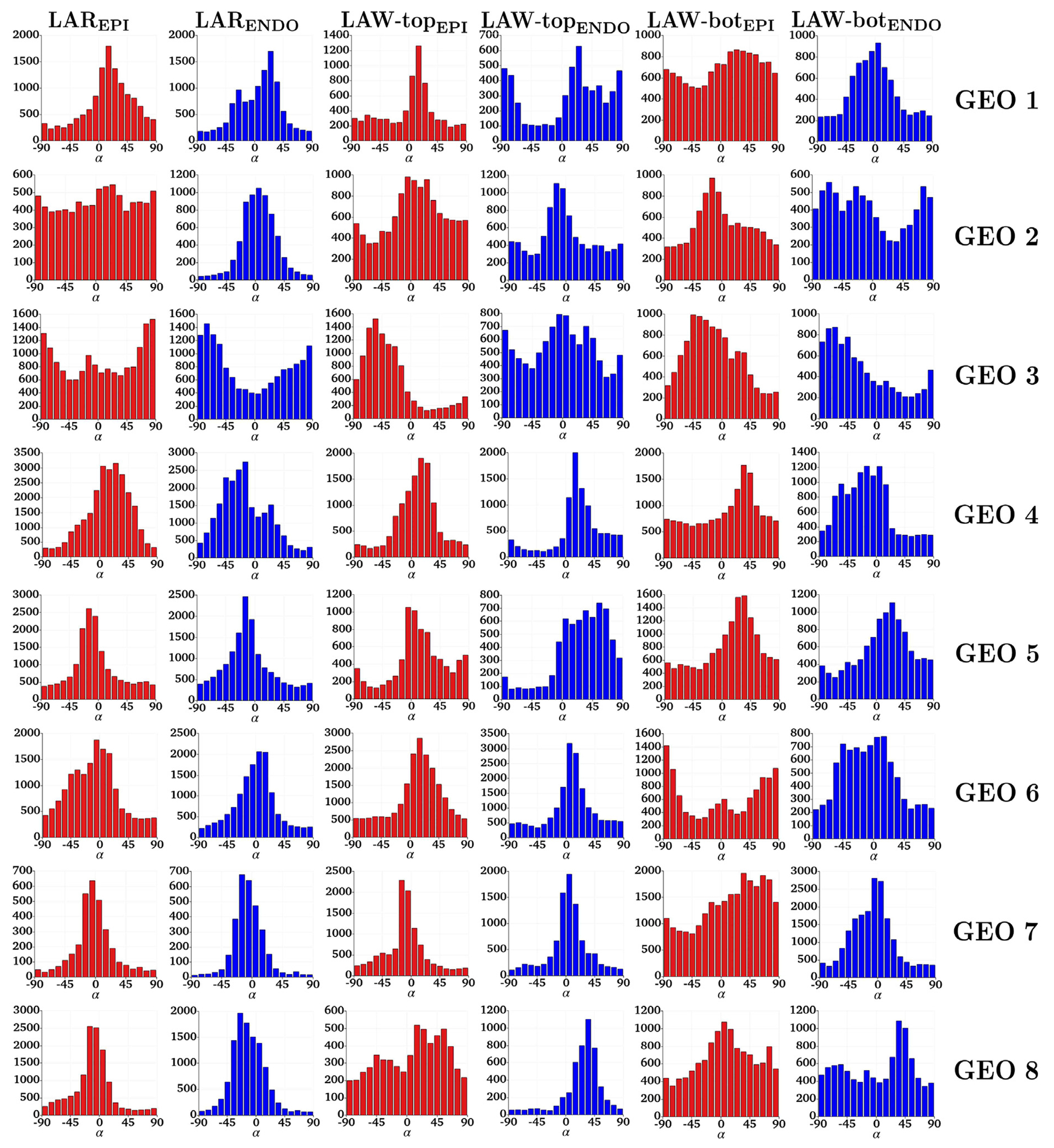
Histograms displaying the measured alpha angle (with respect to LDRBM coordinate axis system) across the eight DTMRI geometries in LA bundles (LAR, LAW-top, LAW-bot) in the sub-epicardial (red) and sub-endocardial (blue) layers.

**Fig. C.5.**
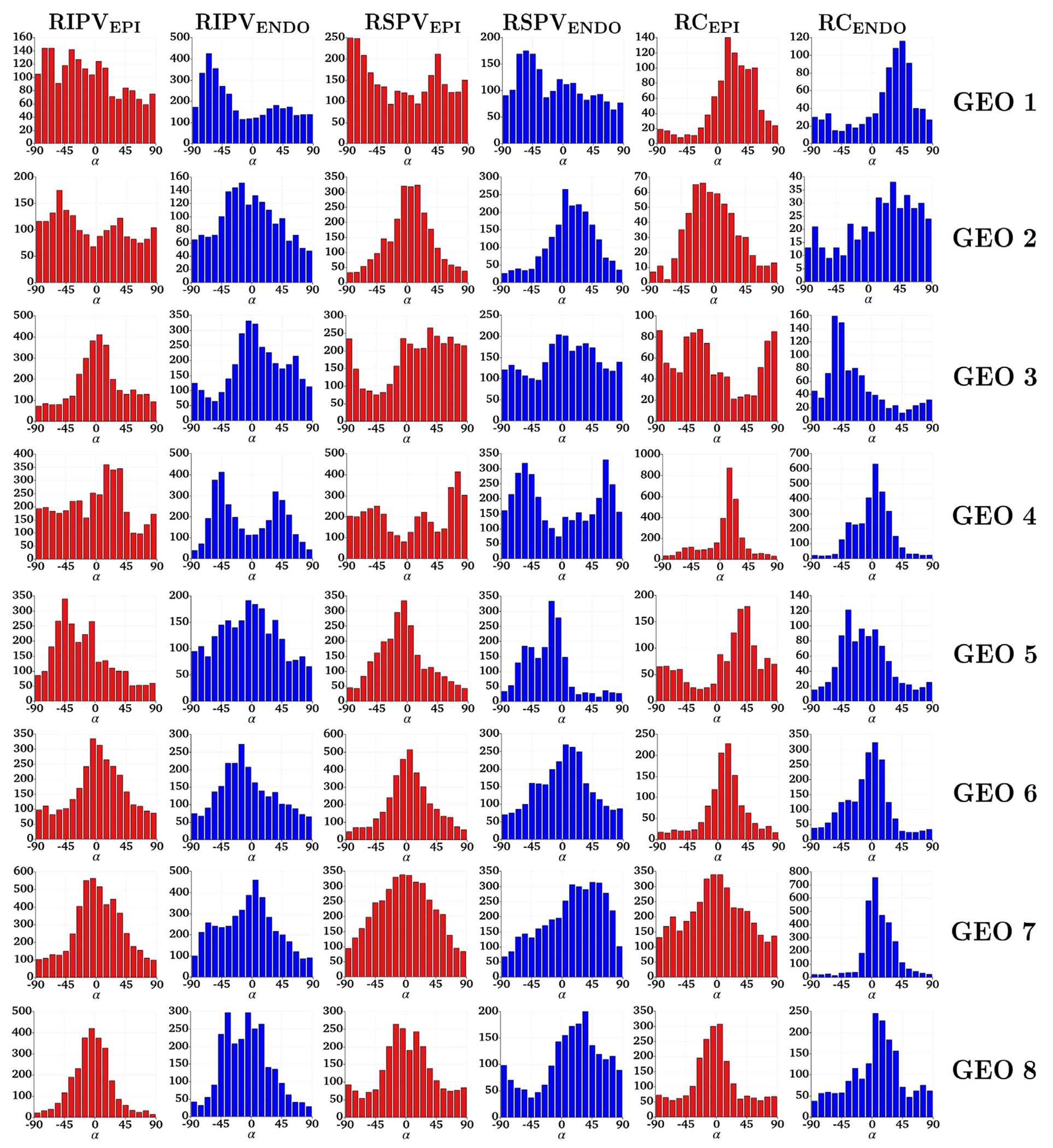
Histograms displaying the measured alpha angle (with respect to LDRBM coordinate axis system) across the eight DTMRI geometries in LA bundles (RIPV, RSPV, RC) in the sub-epicardial (red) and sub-endocardial (blue) layers.

**Fig. C.6.**
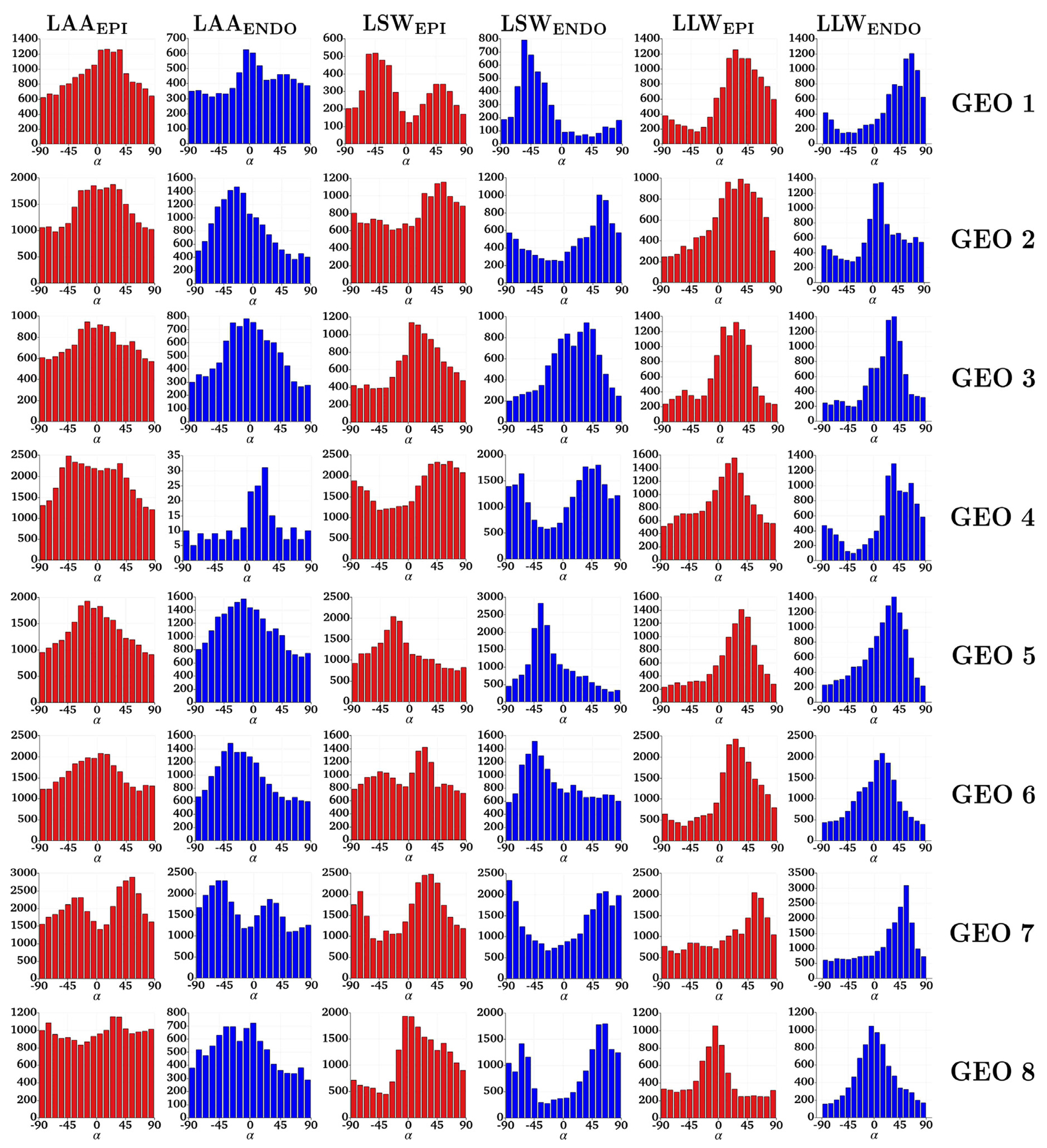
Histograms displaying the measured alpha angle (with respect to LDRBM coordinate axis system) across the eight DTMRI geometries in LA bundles (LAA, LSW, LLW) in the sub-epicardial (red) and sub-endocardial (blue) layers.

**Fig. C.8.**
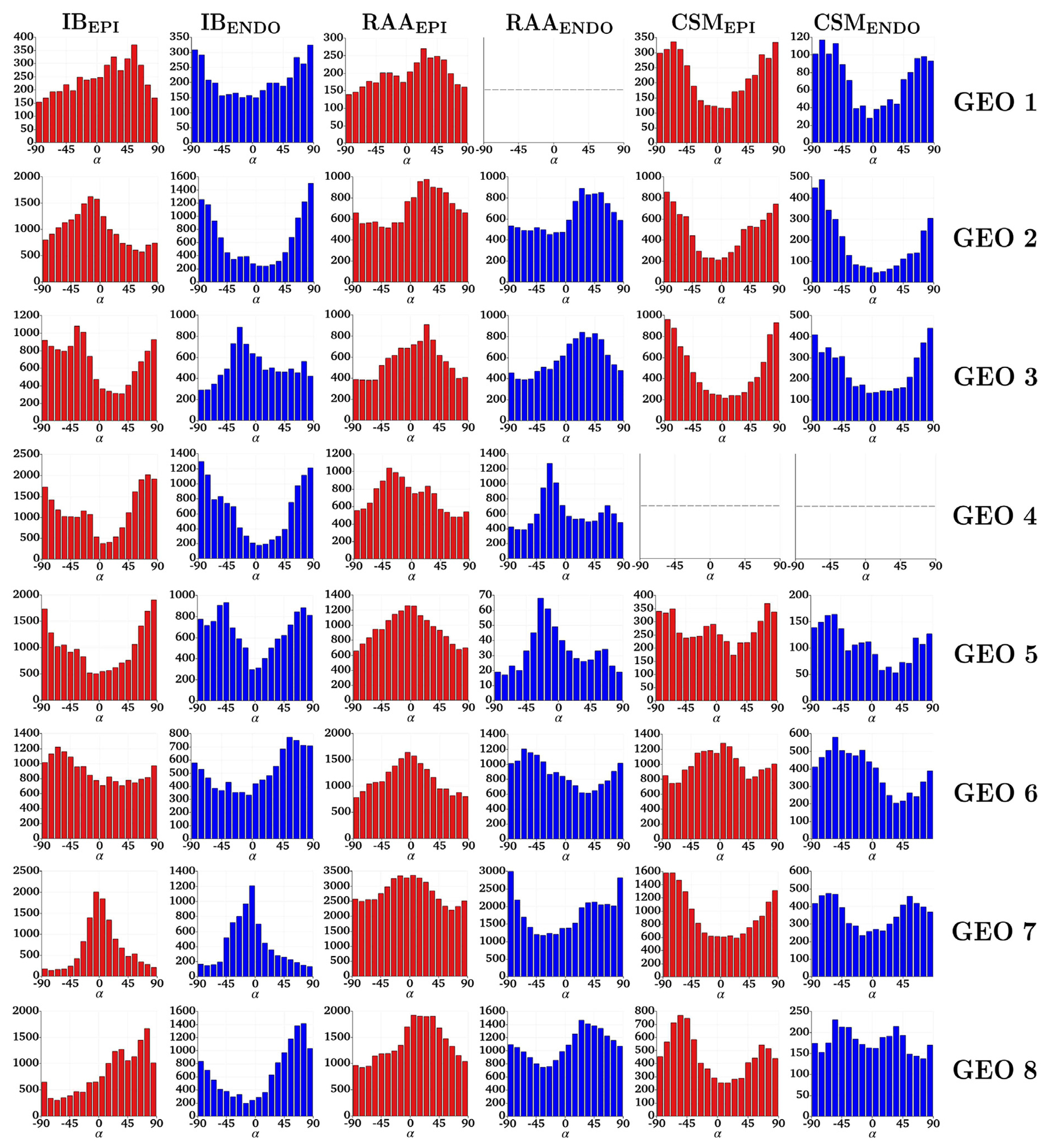
Histograms displaying the measured alpha angle (with respect to LDRBM coordinate axis system) across the eight DTMRI geometries in RA bundles (IB, RAA, CSM) in the sub-epicardial (red) and sub-endocardial (blue) layers.

**Fig. C.9.**
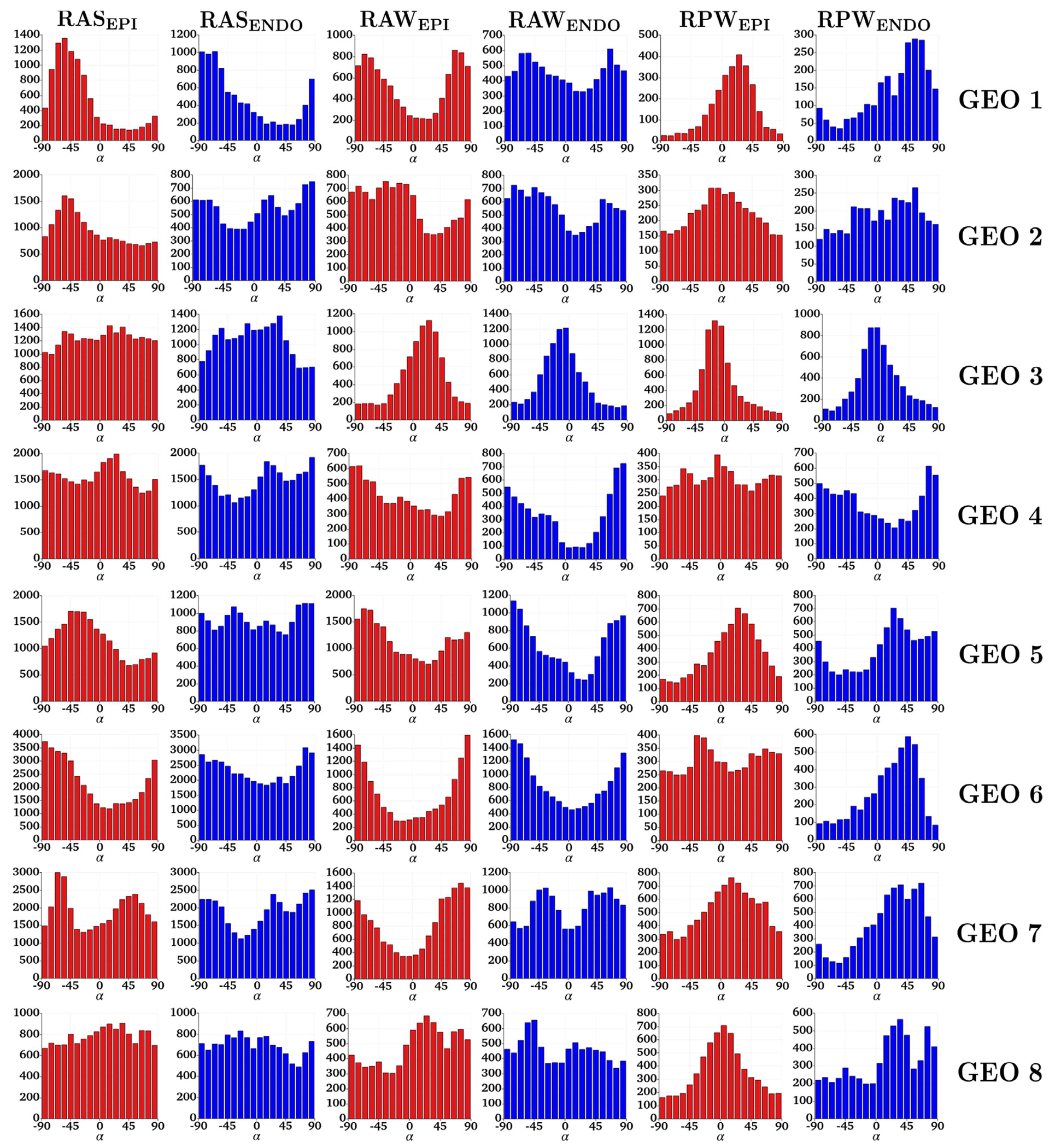
Histograms displaying the measured alpha angle (with respect to LDRBM coordinate axis system) across the eight DTMRI geometries in RA bundles (RAS, RAW, RPW) in the sub-epicardial (red) and sub-endocardial (blue) layers.

**Fig. C.10.**
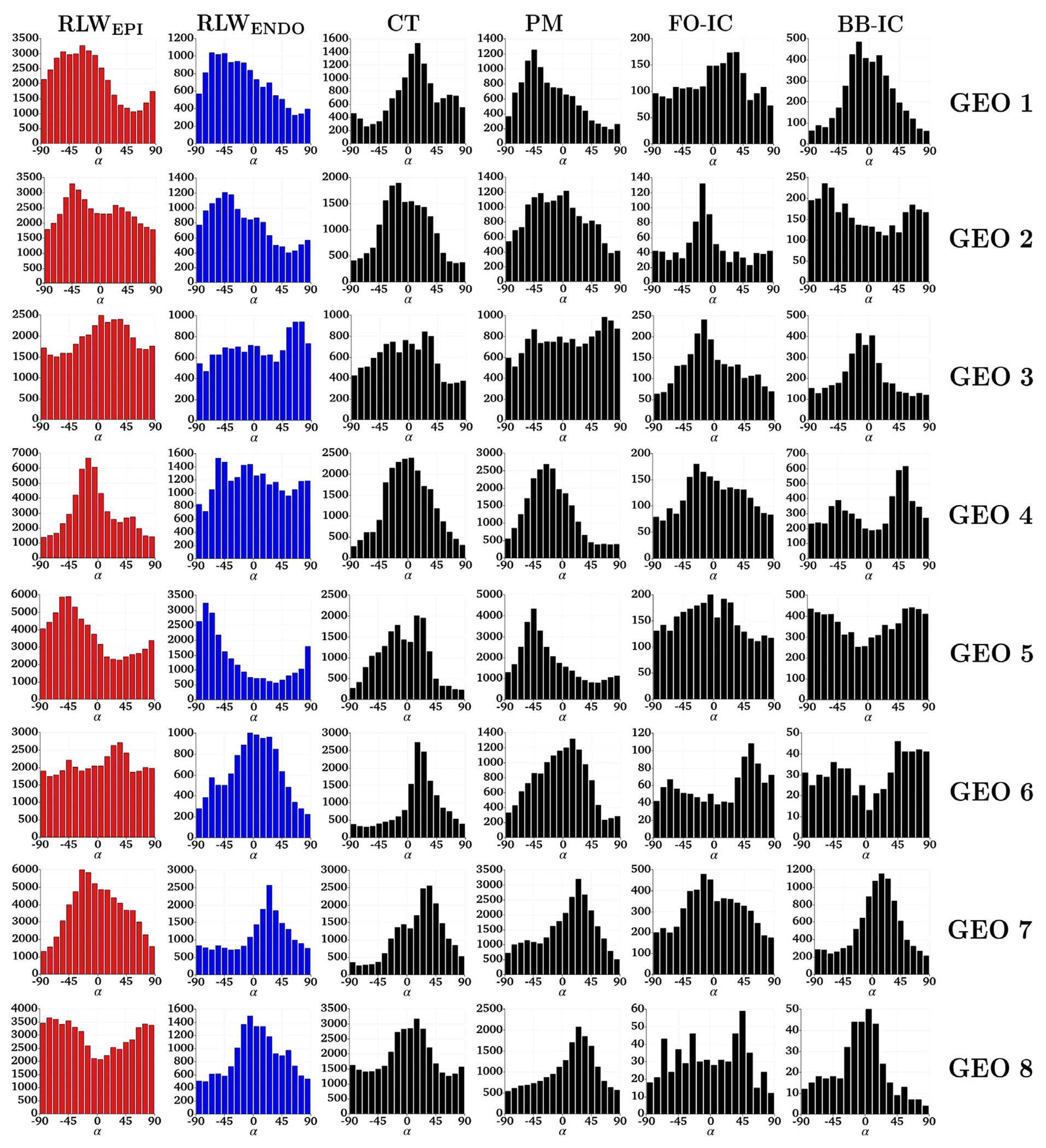
Histograms displaying the measured alpha angle (with respect to LDRBM coordinate axis system) across the eight DTMRI geometries in RLW bundle in the sub-epicardial (red) and sub-endocardial (blue) layers. CT, PM and inter-atrial connections (FO-IC, BB-IC) depicted in black.

**Fig. C.12.**
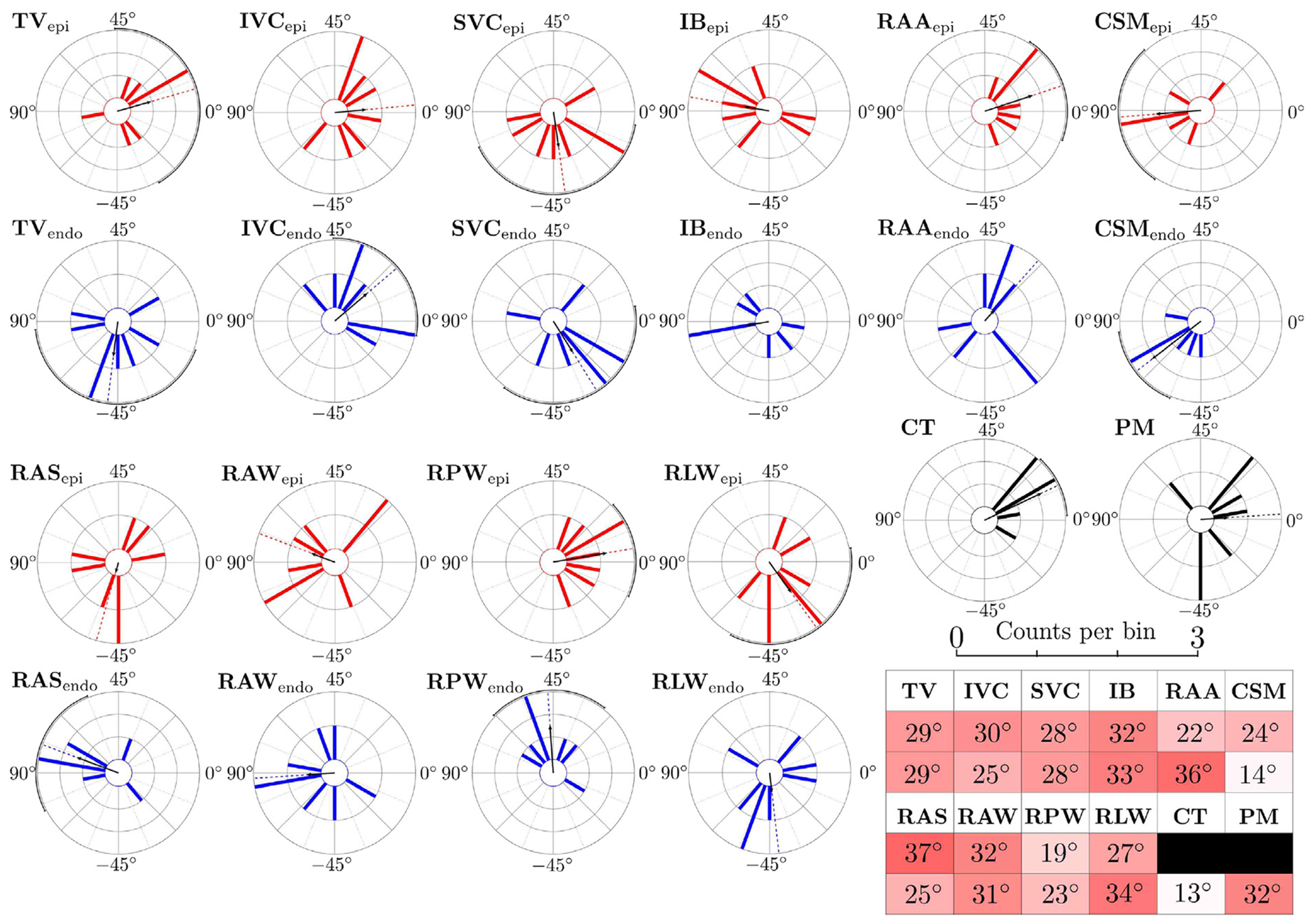
Circular histograms of dominant angle within RA bundles: sub-endocardium (blue), sub-epicardium (red) across eight DTMRI dataset geometries. Histograms of CT and PM bundles depicted in black. Mean angle represented by dotted line. Resultant mean vector depicted in black, with length indicative of angular SD. Corresponding SD values for each bundle shown in table at bottom right corner, with SD color-coded on a scale from minimal (12°) to maximal (37°).

**Fig. C.18.**
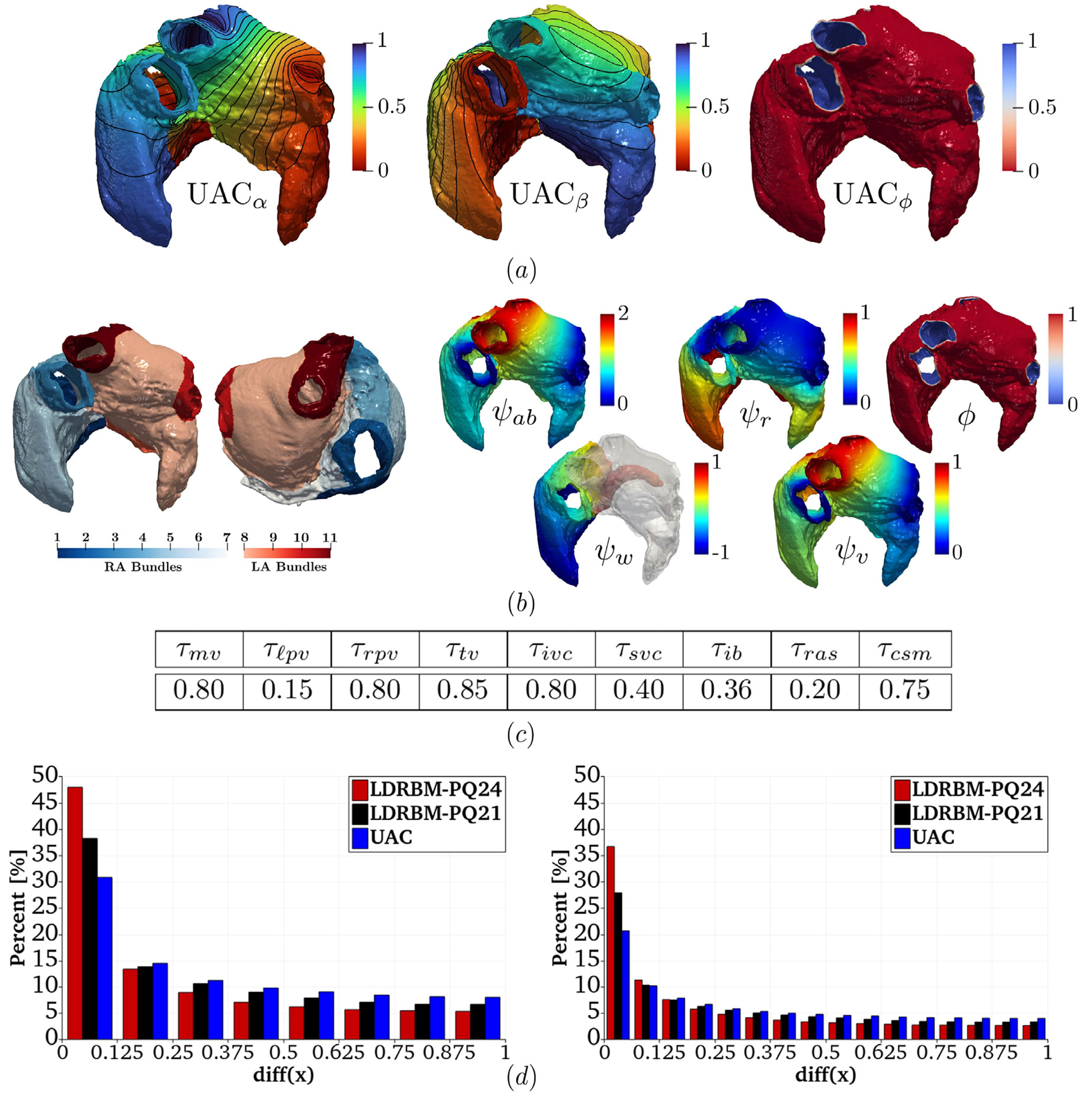
(a) Universal Atrial Coordinates (UAC) showing the three atrial coordinates. For each LA and RA surface, two atrial coordinates were defined: for RA a lateral-septal TV coordinate $\left({\text{UAC}}_{\alpha }\right)$ and an IVC-SVC coordinate $\left({\text{UAC}}_{\beta }\right)$; for the LA, a septal-lateral coordinate $\left({\text{UAC}}_{\alpha }\right)$ and a posterior–anterior coordinate $\left({\text{UAC}}_{\beta }\right);{\text{UAC}}_{\phi }$ represents the transmural coordinate from the endocardium to the epicardium. Refer to [51,57] for further details. (b) Definition of the transmural distance *ϕ* and several intra-atrial distances ϕ for LDRBM–PQ21. These are obtained by solving a Laplace–Dirichlet problems with proper Dirichlet boundary conditions: $\psi_{ab}$ with three different boundary data prescribed on the atrial appendage, the rings of the caval veins and TV for RA, the pulmonary veins and MV ring for LA; $\psi_{v}$ represents the distance between the caval veins for RA and among the pulmonary veins for LA; $\psi_{r}$ stands for the distance between TV and the top of RA, and between MV and the union of the pulmonary veins rings for LA. Moreover, for RA $\psi_{\omega }$ is the distance between the free and the septum walls. Refer to [20,73] for further details. (c) LDRBM-PQ21 bundle parameters for LA and RA used in the comparison presented in Section 3.5. (d) Fiber disparities of different fiber models (LDRBM-PQ24, UAC [59] and LDRBM-PQ21 [20]) relative to DTMRI data. The function diff, computed as $\text{diff}(x)=1-\left|f_{\text{DTMRI}}(x)\cdot f_{i}(x)\right|,$, with i = LDRBM-PQ24, UAC, LDRBM-PQ21. Histograms showing the distribution of the diff function with percentage values per histogram bin of 0.125 (left) and 0.0625 (right). The percentages close to diff ~ 0 indicate the amount of fibers in good agreement with respect to the DTMRI fiber orientations.

## Appendix A. Mesh labeling procedure

The labeling procedure carried out in this work, for the atrial LDRBM (detailed in Section 2.1) consists of the following steps (refer to Fig. A.1 for a graphical representation):

1. Draw the label rings for RSPV, RIPV, LSPV, LIPV, SCV, ICV, MV and TV, namely $\Gamma_{\mathrm{rspv}},\Gamma_{\mathrm{ripv}},\Gamma_{\ell spv},\Gamma_{\ell ipv},\Gamma_{\mathrm{scv}},\Gamma_{\mathrm{icv}},\Gamma_{\mathrm{mv}}$ and $\Gamma_{\mathrm{tv}}$ (see Step 1 in Fig. A.1). For the DTMRI geometries analyzed in this work, characterized by highly complex morphologies, we performed a manual tagging of the valve and vein orifices using the vmtk software and exploiting the vmtksurfacetagger with the drawing functionality [74]. Notably, our orifice labeling process is not a prerequisite for the applicability of the bi-atrial LDRBM. The proposed pipeline is fully compatible with alternative orifice tagging approaches available in the literature [20,67].
2. Identify the epicardium, LA and RA endocardia labels: $\Gamma_{\ell a},\Gamma_{\mathrm{ra}}$ and $\Gamma_{\text{epi}}$ (see Step 2 in Fig. A.1). This is performed by first removing the previously tagged valve and vein orifices from the complete bi-atrial surface. A connectivity-based tagging procedure is then applied to label the disconnected endocardial and epicardial regions, yielding the bi-atrial epicardium and the left and right endocardial surfaces. This procedure can be implemented using the vmtksurfacetagger with the connectivity option [74].
3. Prescribe the endocardial band labels for LA and RA: a lateral and a septal bands $\left(\Gamma_{\ell a}^{\mathrm{lat}},\Gamma_{\ell a}^{\mathrm{sept}}\right)$ for LA, and an anterior and posterior bands ($\Gamma_{\mathrm{ra}}^{\mathrm{ant}},\Gamma_{\mathrm{ra}}^{\mathrm{post}}$) for RA. These regions are identified within the LA and RA endocardia, extracted in the previous Step 2, using the following automated point-selection procedure. The barycenters of LPV and RPV, denoted as LPV_cp_ and RPV_cp_, are calculated as the mean shortest-path distance points between the annulus center points of the superior and inferior veins: LSPV and LIPV for LPV, while RSPV and RIPV for RPV (see Step 3a in Fig. A.1). The lateral and septal MV points, ${MV}_{\ell }$ and ${MV}_{s}$, are determined as follows: ${MV}_{\ell }$ is the mean shortest-path distance point between two points identified by the minimal Euclidean distance on MV from LSPV and LIPV; MV_s_ is defined on MV as the diametrically opposite point from ${MV}_{\ell }$, passing through the annulus center point ${MV}_{cp}$ (see Step 3a in Fig. A.1). The ICV and SCV bottom points, ${ICV}_{\text{bot}}$ and ${SCV}_{\text{bot}}$, are the points of minimal Euclidean distance on TV from the ICV and SCV, respectively (see Step 3b in Fig. A.1). The anterior and posterior TV points, ${TV}_{a}$ and ${TV}_{p}$, are the first intersection points on TV when computing the shortest-path distance between ${SCV}_{\text{bot}}$ (for $\left.{TV}_{a}\right)/{ICV}_{\text{bot}}$ (for ${TV}_{p}$) and the annulus center point ${TV}_{p}$ (see Step 3b in Fig. A.1). Finally, exploit a distance-tagging procedure (e.g., using vmtksurfacedistance and vmtksurfacetagger with the distance–array functionality [74]) in combination with the shortest-path distance evaluated between the selected points, to identify: on the LA endocardium, the lateral band $\Gamma_{\ell a}^{\text{lat}}$ between ${LPV}_{cp}$ and ${MV}_{\ell }$ and the septal band $\Gamma_{\ell a}^{\text{sept}}$ between ${RPV}_{cp}$ and ${MV}_{s}$ (see Step 3a in Fig. A.1); on the RA endocardium, the anterior band $\Gamma_{\mathrm{ra}}^{\text{ant}}$ between ${SCV}_{\text{bot}}$ and ${TV}_{a}$ and the posterior band $\Gamma_{\mathrm{ra}}^{\text{post}}$ between ${ICV}_{\text{bot}}$ and ${TV}_{p}$ (see Step 3b in Fig. A.1).
4. Prescribe the epicardial top-band labels for LA and RA: an anterior and posterior top-bands for LA and a single top-band for RA. The latter are identified on the bi-atrial epicardium, extracted in Step 2, using the following automated point-selection procedure. The LSPV and RSPV top points, namely LSPV_top_ and RSPV_top_, are pinpointed as the points of minimal Euclidean distance from RSPV on LSPV (for LSPV_top_) and from LSPV on RSPV (for RSPV_top_), with an additional penalty for proximity to MV. Similarly, LIPV and RIPV top points, LIPV_top_ and RIPV_top_, are the points of minimal Euclidean distance from RIPV on LIPV (for LIPV_top_) and from LIPV on RIPV (for RIPV_top_), with the same MV proximity penalty (see Step 4a in Fig. A.1). The SCV and ICV top points, SCV_top_ and ICV_top_, are the points of minimal Euclidean distance from ICV on SCV (for SCV_top_) and from SCV on ICV (for ICV_top_), with an additional penalty for proximity to TV (see Step 4b in Fig. A.1). Finally, using a distance-tagging procedure, as described in Step 3, the following bands are identified on the epicardium: the LA anterior top-band $\Gamma_{\mathrm{epi}}^{\text{top},\ell \text{a}}$ between ${LSPV}_{\text{top}}$ and ${RSPV}_{\text{top}}$, the LA posterior top-band $\Gamma_{\text{epi}}^{\text{top},\ell p}$ between ${LIPV}_{\text{top}}$ and ${RIPV}_{\text{top}}$ (see Step 4a in Fig. A.1) and the RA top-band $\Gamma_{\text{epi}}^{\text{top,ra}}$ between ${SCV}_{\text{top}}$ and ${ICV}_{\text{top}}$ (see Step 4b in Fig. A.1).
5. Divide the MV and TV annuli in an anterior $\Gamma_{\mathrm{mv}}^{\mathrm{ant}}$ and posterior $\Gamma_{\mathrm{mv}}^{\text{post}}$ for MV, and anterior $\Gamma_{\mathrm{tv}}^{\text{ant}}$, posterior $\Gamma_{\mathrm{tv}}^{\text{post}}$, septal $\Gamma_{\mathrm{tv}}^{\mathrm{sept}}$ and lateral $\Gamma_{\mathrm{tv}}^{\mathrm{lat}}$ for TV. These subdivisions are performed using as a reference points ${MV}_{\ell }$ and ${MV}_{s}$ for MV, and ${TV}_{a},{TV}_{p},{TV}_{\ell }$ and ${TV}_{s}$ for TV.

## Appendix B. Cardiac electrophysiology modeling

We provide a concise overview of the modeling approach used for representing EP activity in atrial tissue, known as the Eikonal-diffusion model [75,76]. This model, a steady advection-diffusion equation, is employed to reconstruct the macroscopic propagation of action potential excitation wavefronts during the depolarization phase, thereby enabling the recovery of activation time as a spatial function within the myocardium [98–100].

The cardiac muscle is an orthotropic material, arising from its cellular organization into fibers, laminar sheets, and sheet normals, which is mathematically described by the conductivity tensor

(8) $$D=\sigma_{f}f⊗f+\sigma_{s}s⊗s+\sigma_{n}n⊗n,$$

where $\sigma_{f},\sigma_{s}$ and $\sigma_{n}$ are the conductivities along fiber f, sheet s, and sheet-normal n directions.^1^

We model the electrical activity, within atrial domain $\Omega_{\text{bia}}$, with the following Eikonal-diffusion equation

(9) $$\left\{\begin{array}{ll} c_{f}\sqrt{\nabla u\cdot D\nabla u}-\nabla \cdot (D\nabla u)=1 & \text{in}\;\Omega_{\text{bia}}\\ (D\nabla u)\cdot n=0 & \text{on}\;\partial \Omega_{\text{bia}}/S_{\text{san}}\\ u_{A}=u_{0} & \text{on}\;S_{\text{san}} \end{array}\right.,$$

where the unknown $u=u(x):\Omega_{\mathrm{bia}}\to R$, termed activation time, represents the time at which the depolarization wavefront reaches the point $x;S_{\text{san}}$ denotes the portion of the physical boundary where the activation time $u_{0}$ originates, mimicking the onset of atrial activation near the sino-atrial node [35,79], and n is the outward directed unit vector normal to the boundary $\partial \Omega_{\mathrm{bia}}$ of the domain $\Omega_{\mathrm{bia}}$. The parameter $c_{f}$, uniform across the domain $\Omega_{\mathrm{bia}}$, is the velocity of the action potential depolarization planar wavefront along the fiber direction in an infinite cable, under the assumption of a unit surface-to-volume ratio, membrane capacitance, and conductivity^2^ [75,99]. From the Eikonal equation, the following formula for the conduction velocity along the fiber direction can be derived [101, 102]

(10) $$v_{f}=c_{f}\sqrt{\sigma_{f}}.$$

We numerically solve the Eikonal-diffusion problem (9), by recovering the steady-state solution of the following parabolic pseudo-time problem

(11) $$\frac{\partial u}{\partial t}+c_{f}\sqrt{\nabla u\cdot D\nabla u}-\nabla \cdot \left(D\nabla u\right)=1\;\mathrm{in}\;\Omega_{\mathrm{bia}},$$

with the same boundary and initial conditions as in (9), and t representing the pseudo-time [103].

The time discretization of the pseudo-time equation (11) is performed by means of the Backward Difference Formulae of order σ=1,2,… (BDFσ) [104] with an implicit treatment of the transport and diffusion terms

(12) $$\frac{\alpha_{\mathrm{BDF}}u^{n+1}-u_{\mathrm{BDF}\sigma }^{n}}{\Delta t}+c_{f}\sqrt{\nabla u^{n+1}\cdot D\nabla u^{n+1}}-\nabla \cdot \left(D\nabla u^{n+1}\right)=1\;\text{in}\;\Omega_{\mathrm{bia}},$$

where $u^{n}$ denotes the activation time at the n-th time step $t^{n}$, i.e. $u^{n}\approx$ $u\left(t^{n}\right),\Delta t=t^{n+1}-t^{n}$ is the time step size, $\alpha_{\mathrm{BDF}}$ and $u_{BDF\sigma }^{n}$ are the terms associated to the BDFσ time discretization of the time derivative $\frac{\partial u}{\partial t}$. Pseudo-time iterations stop when either $t^{n}\geq T$ for a defined final pseudo-time T, or ${\left\|u^{n+1}-u_{\mathrm{EXT}\sigma }^{n}\right\|}_{L^{2}\left(\Omega_{\text{bia}}\right)}<\epsilon$, with $u_{EXT\sigma }^{n}$ denoting the extrapolation of the solution at time step n as a linear combination of the previous time steps and ϵ a specified tolerance.

We address the non-linear problem arising from the time discretization (12) with the Newton method [103]. Let us define the residual of the problem (12) as

(13) $$L(u)=\frac{\alpha_{\mathrm{BDF}}u-u_{\mathrm{BDF}\sigma }^{n}}{\Delta t}+c_{f}\sqrt{\nabla u\cdot D\nabla u}-\nabla \cdot (D\nabla u)-1.$$

Then, the Newton algorithm amounts to solving for $k=0,\ldots ,k_{max}$

$$\left\{\begin{array}{r} J\left(u_{\left(k\right)}\right)\delta u=L\left(u_{\left(k\right)}\right)\\ u_{\left(k+1\right)}=u_{\left(k\right)}-\delta u \end{array}\right.,$$

where J is the Jacobian operator of L, that is

$$J(u)\delta u=\frac{\alpha_{\text{BDF}}}{\text{Δ}t}\delta u+c_{f}\frac{\left(D+D^{T}\right)\nabla u}{2\sqrt{\nabla u\cdot D\nabla u}}\nabla \delta u-\nabla \cdot (D\nabla \delta u).$$

For the space discretization, we used continuous FE on tetrahedral meshes [76]. The resulting linear systems arising at each pseudo-time step were solved by the GMRES method preconditioned with the AMG preconditioner [105,106].

It is worth emphasizing that the Eikonal-diffusion problem (9) offers superior accuracy compared to the pure Eikonal model (i.e., without considering any diffusive term in (9)), as it takes into account the effects of wavefront curvature or the interaction between a wavefront with either the domain boundaries or with other fronts [75,107]. For more comprehensive insights into the Eikonal-diffusion model, we refer the reader to [75,76,108].

## Appendix C. DTMRI atrial fiber measurements

### C.1. Circular statistics for directional data

All the statistical data analysis performed to estimate the variability of both the regional bundle-dimension parameters and the atrial fiber angles inside each bundle were performed in Matlab (https://www.mathworks.com). In particular, we employed the CircStat toolbox [85] for circular data analysis conducted for the fiber angle statistics, and the CircHist toolbox^3^ to perform the polar angle histograms.

We briefly summarize hereafter the main steps involved in evaluating the mean and standard deviation (SD) values employing the circular statistics. Moreover, we showcase a trivial example to highlight the importance of using such methodology when dealing with directional (angle) data. Due to its circular nature, directional data, like the fiber angles, cannot be analyzed with commonly used statistical techniques, that fail when applied to analyze circular/directional data [85–87]. For further details regarding circular statistics, we refer to [85–87].

Considering N directional observations (angles) $\alpha_{i}\in (0,2\pi ]$ as $\left(\alpha_{1},\ldots ,\alpha_{N}\right)$, the steps to evaluate circular mean and SD, using circular statistics, are resumed hereafter:

1. Angles $\alpha_{i}$ are transformed to unit vectors ri=sin⁡αicos⁡αi;
2. Vectors are vectorial averaged $\bar{r}=\frac{1}{N}\sum_{i=1}^{N}\;r_{i}$, where $\bar{r}$ represent the mean resultant vector;
3. The mean angular direction is recovered using the four quadrant inverse tangent function $\overset{‾}{\alpha }=\mathrm{atan}2\left(x_{\overset{¯}{r}},y_{\bar{r}}\right)$, where $x_{\bar{r}}$ and $y_{\bar{r}}$ are the x and y components of the mean resultant vector $\bar{r}$;
4. The variance, defined as σ=1-R, with $R=\|\bar{r}\|$, is linked to the length of the mean resultant vector R. Hence, the closer σ is to one, the more concentrated the data sample is around the mean direction. Finally, SD is evaluated using the formula $SD=\sqrt{2\sigma }$.

Following the above procedure, we evaluated the mean and SD of the dominant fiber angle measurements for each bundle across the DTMRI dataset (see Section 3.2). The results are illustrated in Figs. C.11 and C.12 and reported in Tables 7 and 8. Notice that to account for the variability range of the fiber angle data, i.e. α∈(-π/2,π/2], we first mapped the angular values into β∈(0,2π], by means of β=2α+π, then we recovered the mean angular vaule $\overset{‾}{\beta }$ and the related ${SD}_{\beta }$. Finally, we went back to the mean and SD angular values using $\overset{‾}{\alpha }=(\overset{‾}{\beta }-\pi )/2$ and $SD={SD}_{\beta }/2$.

To highlight that usual summary statistics, such as the sample mean and linear SD, cannot be used with angular values, let consider three angles $\beta =\left(10^{\circ },30^{\circ },350^{\circ }\right)$. Classical statistical tools, for computing the mean value ${\overset{‾}{\beta }}_{l}$ and the ${SD}_{\beta ,l}$ result in $130^{\circ }$ and $191^{\circ }$, respectively. However, all the data are roughly pointing towards $0^{\circ }$. Conversely, if we use circular statistics, we retrieve the correct value of ${\overset{‾}{\beta }}_{c}=10^{\circ }$ and ${SD}_{\beta ,c}=16^{\circ }$.

**Table C.4:** Bundle parameter values selected for the inter-atrial connections (BIA) across the eight DTMRI geometries. Mean and Standard Deviation (SD) values are provided, with SD color-coded on a scale from minimal (0) to maximal (0.32).

Type	τ	Geo 1	Geo 2	Geo 3	Geo 4	Geo 5	Geo 6	Geo 7	Geo 8	Mean	SD
IC	$\tau_{\mathrm{bb}}^{ic,r}$	−0.80	−0.50	−0.70	−0.50	−0.50	−0.50	−0.75	−0.50	−**0.59**	0.13
	$\tau_{\mathrm{bb}}^{ic,\ell }$	0.80	0.95	0.80	0.90	0.80	0.99	0.85	0.95	**0.88**	0.08
	$\tau_{bb}^{ic}$	0.33	0.52	0.61	0.55	0.21	0.71	0.54	0.57	**0.50**	0.16
	$\tau_{\mathrm{fo}}^{ic,r}$	−0.80	−0.80	−0.80	−0.80	−0.80	−0.80	−0.80	−0.80	−**0.80**	0.00
	$\tau_{\mathrm{fo}}^{ic,\ell }$	0.80	0.80	0.80	0.80	0.80	0.80	0.80	0.80	**0.80**	0.00
	$\tau_{fo}^{ic}$	0.41	0.11	0.66	0.54	0.54	0.38	0.29	−0.03	**0.36**	0.23
	$\tau_{\mathrm{fo}}^{ic,in}$	0.44	0.17	0.68	0.55	0.56	0.42	0.32	0.03	**0.40**	0.21
	$\tau_{cs}^{ic,r}$	–	−0.45	–	–	–	–	–	–	−**0.45**	0.00
	$\tau_{cs}^{ic,\ell }$	–	0.45	–	–	–	–	–	–	**0.45**	0.00
	$\tau_{cs}^{ic}$	–	0.40	–	–	–	–	–	–	**0.40**	0.00

**Table C.5:** Bundle parameter values selected for LA bundles across the eight DTMRI geometries. Mean and Standard Deviation (SD) values are provided, with SD color-coded on a scale from minimal (0) to maximal (0.32).

Type	τ	Geo 1	Geo 2	Geo 3	Geo 4	Geo 5	Geo 6	Geo 7	Geo 8	Mean	SD
LA	$\tau_{\mathrm{mv}}$	0.80	0.85	0.83	0.80	0.85	0.80	0.80	0.85	**0.82**	0.02
	$\tau_{\ell pv}$	0.20	0.15	0.18	0.20	0.20	0.20	0.15	0.15	**0.18**	0.02
	$\tau_{\ell pv}^{up}$	0.25	0.15	0.25	0.25	0.25	0.25	0.15	0.25	**0.22**	0.04
	$\tau_{\ell ipv}$	−0.40	−0.70	−0.40	−0.40	−0.40	−0.50	−0.40	−0.40	−**0.45**	0.10
	$\tau_{\ell spv}$	0.20	0.20	0.20	0.10	0.10	0.40	0.30	0.20	**0.21**	0.10
	$\tau_{\mathrm{rpv}}$	0.80	0.80	0.85	0.85	0.85	0.86	0.80	0.84	**0.83**	0.03
	$\tau_{\mathrm{rpv}}^{up}$	0.13	0.13	0.17	0.15	0.12	0.13	0.15	0.15	**0.14**	0.02
	$\tau_{\text{ripu}}$	−0.30	−0.10	−0.15	−0.60	−0.20	−0.30	−0.40	−0.40	−**0.31**	0.16
	$\tau_{\mathrm{rspv}}$	0.10	0.20	0.35	0.40	0.30	0.30	0.40	0.40	**0.31**	0.11
	$\tau_{\ell aa}$	0.18	−0.16	0.53	0.34	0.35	0.15	0.12	−0.25	**0.16**	0.26
	$\tau_{\ell as}$	0.015	−0.030	−0.010	−0.015	−0.010	−0.040	0.025	−0.030	−**0.012**	0.02
	$\tau_{p\ell w}^{\ell a}$	0.35	0.38	0.35	0.20	0.35	0.52	0.36	0.32	**0.35**	0.09
	$\tau_{\text{pℓw}}^{\text{ℓa,up}}$	0.25	0.25	0.11	0.25	0.15	0.12	0.25	0.25	**0.19**	0.06
	$\tau_{a\ell w}^{\ell a}$	0.26	0.24	0.20	0.25	0.37	0.22	0.28	0.25	**0.26**	0.05
	$\tau_{\mathrm{psw}}^{\ell a}$	0.88	0.83	0.89	0.75	0.78	0.93	0.80	0.78	**0.83**	0.06
	$\tau_{\mathrm{psw}}^{\ell a,up}$	0.25	0.25	0.10	0.25	0.15	0.25	0.25	0.25	**0.22**	0.06
	$\tau_{asw}^{\ell a}$	0.93	0.85	0.85	0.82	0.90	0.80	0.85	0.80	**0.85**	0.04
	$\tau_{\ell ar}$	0.28	0.10	0.18	0.38	0.20	0.10	0.33	0.15	**0.21**	0.10
	$\tau_{\ell aw}$	0.42	0.54	0.46	0.50	0.50	0.56	0.45	0.30	**0.47**	0.08
	$\tau_{\mathrm{bb}}$	0.45	0.41	0.35	0.25	0.28	0.25	0.38	0.42	**0.35**	0.08

**Table C.6:** Bundle parameter values selected for RA bundles across the eight DTMRI geometries. Mean and Standard Deviation (SD) values are provided, with SD color-coded on a scale from minimal (0) to maximal (0.32).

Type	τ	Geo 1	Geo 2	Geo 3	Geo 4	Geo 5	Geo 6	Geo 7	Geo 8	Mean	SD
RA	$\tau_{\mathrm{tv}}$	0.80	0.85	0.80	0.85	0.85	0.85	0.85	0.85	**0.84**	0.02
	$\tau_{\mathrm{tv}}^{aa}$	0.60	0.60	0.60	0.60	0.60	0.60	0.60	0.60	**0.60**	0.00
	$\tau_{\mathrm{icv}}$	0.85	0.80	0.90	0.90	0.85	0.90	0.80	0.85	**0.86**	0.04
	$\tau_{\mathrm{icv}}^{up}$	0.25	0.25	0.25	0.25	0.15	0.25	0.30	0.25	**0.24**	0.04
	$\tau_{\mathrm{scv}}$	0.20	0.30	0.35	0.40	0.20	0.50	0.40	0.25	**0.32**	0.11
	$\tau_{\mathrm{scv}}^{up}$	0.25	0.30	0.30	0.20	0.16	0.30	0.40	0.28	**0.27**	0.07
	$\tau_{\text{raa}}$	0.25	0.33	0.38	0.34	0.17	0.40	0.39	0.15	**0.30**	0.10
	$\tau_{\mathrm{raa}}^{w}$	−0.25	−0.32	−0.05	−0.10	−0.22	−0.12	−0.10	−0.33	−**0.18**	0.11
	$\tau_{\mathrm{raa}}^{up}$	0.64	0.50	0.42	0.20	0.54	0.42	0.63	0.60	**0.49**	0.15
	$\tau_{\text{csm}}$	0.30	0.65	0.50	–	−0.30	0.25	0.75	0.45	**0.37**	0.32
	$\tau_{\mathrm{csm}}^{v}$	0.40	0.55	0.72	–	0.30	0.50	0.60	0.40	**0.51**	0.13
	$\tau_{\text{ras}}$	−0.05	−0.15	−0.02	−0.16	−0.15	−0.02	−0.01	−0.10	−**0.08**	0.06
	$\tau_{asw}^{\mathrm{ra}}$	0.85	0.65	0.60	0.65	0.75	0.75	0.80	0.60	**0.71**	0.09
	$\tau_{asw}^{\text{ra,up}}$	0.50	0.20	0.25	0.25	0.25	0.15	0.25	0.25	**0.26**	0.10
	$\tau_{a\ell w}^{\mathrm{ra}}$	0.70	0.65	0.80	0.65	0.84	0.80	0.66	0.78	**0.73**	0.08
	$\tau_{\mathrm{psw}}^{\mathrm{ra}}$	−0.55	−0.70	−0.70	−0.70	−0.75	−0.55	−0.40	−0.67	−**0.63**	0.12
	$\tau_{\mathrm{psw}}^{\text{ra,up}}$	0.75	0.40	0.50	0.40	0.50	0.50	0.50	0.50	**0.51**	0.11
	$\tau_{p\ell w}^{\mathrm{ra}}$	−0.55	−0.60	−0.70	−0.75	−0.70	−0.60	−0.46	−0.70	−**0.63**	0.10
	$\tau_{p\ell w}^{\text{ra,up}}$	0.70	0.40	0.50	0.25	0.50	0.50	0.50	0.50	**0.48**	0.12
	$\tau_{ib}^{\ell }$	0.10	0.34	0.30	0.20	0.33	0.35	0.36	0.53	**0.31**	0.12
	$\tau_{ib}^{s}$	0.58	0.25	0.10	0.22	0.35	0.10	0.20	0.22	**0.25**	0.15
	$\tau_{ct}^{+}$	−0.45	0.01	0.10	0.00	−0.23	−0.44	−0.20	−0.25	−**0.18**	0.20
	$\tau_{ct}^{-}$	−0.51	−0.12	0.00	−0.33	−0.43	−0.54	−0.33	−0.40	−**0.33**	0.19
	$\tau_{ct}^{\phi }$	0.60	0.60	0.60	0.60	0.60	0.60	0.60	0.60	**0.60**	0.00
	pm_*end*_	0.86	0.83	0.92	0.91	0.89	0.86	0.80	0.86	**0.87**	0.04
	pm_*tk*_	0.015	0.015	0.015	0.015	0.015	0.015	0.015	0.015	**0.015**	0.00
	pm_*rg*_	0.015	0.015	0.015	0.015	0.015	0.015	0.015	0.015	**0.015**	0.00

	$N_{\text{pm}}$	17	14	16	17	19	16	13	23	**17**	3

**Table 7:** Dominant angle *α* values measured for right atrial bundles (RA) in the sub-epicardium (*epi*) and sub-endocardium (*endo*) across the eight DTMRI geometries. Circular Mean and Standard Deviation (SD) values are provided, with SD color-coded on a scale from minimal $\left(12^{\circ }\right)$ to maximal $\left(37^{\circ }\right)$. All values are expressed in degrees.

Type	α	Geo 1	Geo 2	Geo 3	Geo 4	Geo 5	Geo 6	Geo 7	Geo 8	Mean	SD
RA	$\alpha_{\mathrm{epi}}^{\mathrm{tv}}$	90	15	25	30	10	−35	−25	10	**8**	29
	$\alpha_{\text{endo}}^{\text{tv}}$	85	−85	−15	−35	10	−55	−55	−45	−**49**	29
	$\alpha_{\mathrm{epi}}^{\text{ivc}}$	35	−70	−25	−5	35	10	20	−35	**3**	30
	$\alpha_{\text{endo}}^{\text{ivc}}$	40	65	25	−20	−10	30	35	−5	**20**	25
	$\alpha_{\mathrm{epi}}^{svc}$	−35	−50	−15	−55	−85	−75	−15	15	−**41**	28
	$\alpha_{\text{endo}}^{\text{svc}}$	−15	−60	−25	−40	−15	85	−30	25	−**29**	28
	$\alpha_{\mathrm{epi}}^{\text{raa}}$	25	25	25	−35	−15	−5	5	35	**9**	22
	$\alpha_{\text{endo}}^{\text{raa}}$	25	35	45	−25	−25	−65	90	30	**24**	36
	$\alpha_{\mathrm{epi}}^{\mathrm{csm}}$	90	−85	−80	–	75	25	−85	−55	−**87**	24
	$\alpha_{\text{endo}}^{\text{csm}}$	−75	−75	80	–	−60	−65	−75	−50	−**71**	14
	$\alpha_{\mathrm{epi}}^{\text{ras}}$	−55	−50	80	20	−45	−85	0	35	−**53**	37
	$\alpha_{\text{endo}}^{\text{ras}}$	−85	80	35	85	75	75	80	−30	**80**	25
	$\alpha_{\mathrm{epi}}^{\text{rpw}}$	25	10	−15	−5	30	−35	15	5	**5**	19
	$\alpha_{\text{endo}}^{\text{rpw}}$	55	55	−15	75	25	50	60	35	**47**	23
	$\alpha_{\mathrm{epi}}^{\text{raw}}$	65	−35	25	−80	−75	90	75	25	**80**	32
	$\alpha_{\text{endo}}^{\text{raw}}$	45	−70	−15	80	−85	−85	55	−45	−**88**	31
	$\alpha_{\text{epi}}^{\text{ib}}$	55	−15	−85	75	85	−65	−5	75	**85**	32
	$\alpha_{\text{endo}}^{\text{ib}}$	90	90	−25	90	−45	60	−5	70	−**85**	33
	$\alpha_{\mathrm{epi}}^{r\ell w}$	−25	−45	10	−15	−45	35	−30	−70	−**27**	27
	$\alpha_{\text{endo}}^{\text{rℓw}}$	−60	−45	70	−55	−70	0	25	−5	−**42**	34
	$\alpha_{\text{endo}}^{ct}$	15	−20	25	5	20	15	20	15	**13**	13
	$\alpha_{\text{endo}}^{\text{pm}}$	−45	5	65	−25	−45	15	25	25	**2**	32

**Table 8:** Dominant angle *a* values measured for inter-atrial connections (BIA) and left atrial bundles (LA) in the sub-epicardium (*epi*) and sub-endocardium (*endo*) across the eight DTMRI geometries. Circular Mean and Standard Deviation (SD) values are provided, with SD color-coded on a scale from minimal (12°) to maximal (37°). All values are expressed in degrees.

Type	α	Geo 1	Geo 2	Geo 3	Geo 4	Geo 5	Geo 6	Geo 7	Geo 8	Mean	SD
BIA	$\alpha_{\mathrm{epi}}^{\text{bb,ic}}$	−15	−65	−15	55	65	45	15	5	**20**	33
	$\alpha_{\mathrm{epi}}^{fo,ic}$	30	−15	−15	−25	−5	55	−10	45	**3**	27

LA	$\alpha_{\mathrm{epi}}^{mv}$	15	15	−5	5	15	40	20	5	**13**	12
	$\alpha_{\text{endo}}^{\text{mv}}$	65	20	5	45	15	45	35	10	**29**	19
	$\alpha_{\mathrm{epi}}^{\ell ipv}$	30	5	−85	5	15	10	−5	20	**12**	22
	$\alpha_{\text{endo}}^{\text{ℓipv}}$	20	10	90	15	5	15	−15	5	**10**	22
	$\alpha_{\text{epi}}^{\ell spv}$	25	30	−5	35	−10	−15	−10	−10	**4**	19
	$\alpha_{\text{endo}}^{\text{ℓspv}}$	15	15	−5	−5	−15	−25	−15	−25	−**8**	14
	$\alpha_{\mathrm{epi}}^{\ell c}$	−20	−5	−45	−25	−55	35	15	−85	−**22**	31
	$\alpha_{\text{endo}}^{\ell c}$	−20	90	−25	−15	15	20	15	−5	−**3**	26
	$\alpha_{\mathrm{epi}}^{\mathrm{ripv}}$	−70	−55	5	20	−45	−5	−5	−5	−**16**	28
	$\alpha_{\text{endo}}^{\text{ripv}}$	−65	−15	−5	−45	5	−15	5	−35	−**19**	22
	$\alpha_{\mathrm{epi}}^{\text{rspv}}$	−80	5	35	75	−5	5	−5	−15	**5**	31
	$\alpha_{\text{endo}}^{\text{rspv}}$	−55	10	−5	−65	−15	10	40	35	**2**	31
	$\alpha_{\text{epi}}^{\text{rc}}$	20	−20	−35	15	40	15	−10	0	**3**	21
	$\alpha_{\text{endo}}^{\text{rc}}$	45	35	−55	5	−35	0	5	10	**5**	27
	$\alpha_{\mathrm{epi}}^{\ell aa}$	30	40	−15	−45	−15	20	55	−70	**10**	34
	$\alpha_{\text{endo}}^{\text{ℓaa}}$	0	−25	−5	25	−55	−35	−50	5	−**17**	25
	$\alpha_{\text{epi}}^{\text{ℓas}}$	−75	60	65	65	35	25	50	15	**50**	24
	$\alpha_{\text{endo}}^{\text{ℓas}}$	−35	−20	45	−25	15	−55	5	−45	−**19**	28
	$\alpha_{\mathrm{epi}}^{\ell \ell w}$	25	35	25	25	35	25	55	−5	**28**	15
	$\alpha_{\text{endo}}^{\text{ℓℓw}}$	60	10	35	35	35	15	50	−5	**30**	19
	$\alpha_{\mathrm{epi}}^{\ell sw}$	−25	50	30	65	−25	30	30	0	**22**	28
	$\alpha_{\text{endo}}^{\ell \text{sw}}$	−55	60	45	50	−35	−45	70	65	**73**	34
	$\alpha_{\mathrm{epi}}^{\ell ar}$	25	90	85	30	−15	0	0	0	**12**	31
	$\alpha_{\text{endo}}^{\text{ℓar}}$	35	5	−75	−45	−15	15	5	−30	−**8**	29
	$\alpha_{\mathrm{epi}}^{\ell aw,t}$	20	0	−50	20	−5	25	−15	55	**8**	26
	$\alpha_{\text{endo}}^{\ell aw,t}$	25	−15	0	15	55	20	5	45	**18**	21
	$\alpha_{\mathrm{epi}}^{\ell aw,b}$	20	−15	−30	40	35	−85	5	5	**10**	29
	$\alpha_{\text{endo}}^{\ell aw,b}$	5	−60	−70	5	25	−40	−5	35	−**6**	32
	$\alpha_{\mathrm{epi}}^{\mathrm{bb}}$	65	55	5	45	35	10	−25	65	**36**	27
	$\alpha_{\text{endo}}^{\text{bb}}$	50	−35	−80	35	45	−10	45	85	**50**	33
